# A Cell Cycle Progression-Derived Gene Signature to Predict Prognosis and Therapeutic Response in Hepatocellular Carcinoma

**DOI:** 10.1155/2021/1986159

**Published:** 2021-10-21

**Authors:** Yongfeng Hui, Junzhi Leng, Dong Jin, Di Liu, Genwang Wang, Qi Wang, Yanyang Wang

**Affiliations:** ^1^Department of Hepatobiliary Surgery, General Hospital of Ningxia Medical University, Yinchuan, 750004 Ningxia, China; ^2^Department of Radiation Oncology, General Hospital of Ningxia Medical University, Yinchuan, 750004 Ningxia, China

## Abstract

**Objective:**

Dysregulation of cell cycle progression (CCP) is one of the hallmarks of cancer. Here, our study is aimed at developing a CCP-derived gene signature for predicting high-risk population of hepatocellular carcinoma (HCC).

**Methods:**

Our study retrospectively analyzed the transcriptome profiling and clinical information of HCC patients from The Cancer Genome Atlas (TCGA) and International Cancer Genome Consortium (ICGC) projects. Uni- and multivariate cox regression models were conducted for identifying which hallmarks of cancer were risk factors of HCC. CCP-derived gene signature was developed with LASSO method. The predictive efficacy was verified by ROC curves and subgroup analyses. A nomogram was then generated and validated by ROC, calibration, and decisive curves. Immune cell infiltration was estimated with ssGSEA method. Potential small molecular compounds were predicted via CTRP and CMap analyses. The response to chemotherapeutic agents was evaluated based on the GDSC project.

**Results:**

Among hallmarks of cancer, CCP was identified as a dominant risk factor for HCC prognosis. CCP-derived gene signature displayed the favorable predictive efficacy in HCC prognosis independent of other clinicopathological parameters. A nomogram was generated for optimizing risk stratification and quantifying risk evaluation. CCP-derived signature was in relation to immune cell infiltration, HLA, and immune checkpoint expression. Combining CTRP and CMap analyses, fluvastatin was identified as a promising therapeutic agent against HCC. Furthermore, CCP-derived signature might be applied for predicting the response to doxorubicin and gemcitabine.

**Conclusion:**

Collectively, CCP-derived gene signature was a promising marker in prediction of survival outcomes and therapeutic responses for HCC patients.

## 1. Introduction

Hepatocellular carcinoma (HCC), an aggressive malignancy with undesirable prognosis, occupies 85% of liver cancer cases, which usually develops in the context of chronic liver diseases [1]. The complex etiology and highly intratumoral and intertumoral heterogeneity make prognosis prediction more challenging [2]. The current TNM staging system is not sufficient for precise therapy decision-making and prognostic prediction for HCC patients. Emerging evidence demonstrates that genomic signatures can be applied for risk stratification and prognostic prediction in HCC [3–5]. Nevertheless, due to insufficient sample size, interpatient, intertumoral, and intratumoral heterogeneity, and technical bias, most of prognostic models possess low reproducibility, which cannot be utilized for clinical routine practice [6]. Hence, new methods of identifying high-risk subgroups of HCC patients will bring great implications to personalized cancer care.

The hallmarks of cancer include six biological abilities acquired during the multistep progression of cancers, including sustained proliferation, evasion of growth inhibitors, cell death resistance, replicative immortality, angiogenesis, invasion activation, metastases, metabolic reprogramming, and immune escape [7]. Among them, cell cycle progression (CCP) is a key biological event that has a controlled regulation in normal cells, but it nearly generally becomes abnormal or dysregulated in tumor cells [8]. Evidence suggests that CCP could be a useful biomarker in prediction of recurrence and metastasis following surgical resection in several cancer types such as breast cancer and prostate cancer [9–11]. Aberrations of CCP-related genes are frequently found across diverse human neoplastic processes, and their expression profiling possesses wide ranging prognostic significance [12]. In our research, CCP was identified as a dominant risk factor of HCC survival outcomes. Accurate bioinformatic and machine learning methods were adopted for screening reliable candidate variables as well as building a personalized CCP-derived gene signature in prediction of prognosis and the response to immune- and chemotherapies for HCC patients.

## 2. Materials and Methods

### 2.1. Data Acquisition

This study gathered the mRNA expression profiling and clinical features of HCC patients from the Cancer Genome Atlas (TCGA; https://cancergenome.nih.gov/) [13] and International Cancer Genome Consortium (ICGC; https://dcc.icgc.org/) [14]. Only patients with complete follow-up time and vital status were included in this study. In total, we finally retrieved 368 HCC patients from TCGA-LIHC cohort (Supplementary table 1) and 260 HCC patients from ICGC (LIRI-JP) cohort for further analysis. Then, FPKM value was converted to TPM value. The somatic mutation data in Mutation Annotation Format (MAF) of HCC were downloaded from TCGA database, which were analyzed through maftools package [15].

### 2.2. Quantification of Hallmarks of Cancer

The gene sets of hallmarks of cancer raised by Hanahan and Weinberg [7] including glycolysis, CCP, angiogenesis, apoptosis, DNA repair, epithelial-mesenchymal transition (EMT), hypoxia, inflammation, and stemness were acquired from the MSigDB database (http://software.broadinstitute.org/gsea/index.jsp) [16]. Single sample gene set enrichment analysis (ssGSEA) was adopted to quantify the activity of above hallmarks of cancer with gene set variation analysis (GSVA) [17]. Immune infiltration was quantified using Cell type Identification By Estimating Relative Subsets Of RNA Transcripts (CIBERSORT) computational method [18]. The ssGSEA and immune infiltration scores were scaled with *Z*-score. Uni- and multivariate cox regression models were conducted to evaluate the prognostic implications of above hallmarks of cancer in TCGA-LIHC cohort. Supplementary table 2 listed the gene sets of hallmarks of cancer.

### 2.3. Differential Expression Analysis

CCP-relevant genes were identified through presenting differential expression analysis between high and low CCP score groups via limma package. The criteria were set as ∣fold − change | >2 and adjusted *p* < 0.05.

### 2.4. Functional Enrichment Analysis

Gene Ontology (GO) and Kyoto Encyclopedia of Genes and Genomes (KEGG) enrichment analyses of CCP-relevant genes were conducted through adopting clusterProfiler package [19]. GO covered three categories: biological process, cellular component, and molecular function.

### 2.5. Establishment and Verification of a Prognostic Gene Signature

Univariate Cox regression models were conducted to assess the association between CCP-relevant genes and HCC prognosis in TCGA-LIHC cohort. CCP-relevant genes with *p* < 0.05 were included for least absolute shrinkage and selection operator- (LASSO-) penalized Cox regression analysis utilizing glmnet package [20]. Variable selection and shrinkage were carried out. Penalty parameter (*λ*) of this model was determined through tenfold crossverification in line with the *λ* value that corresponded to the lowest partial likelihood deviance. The risk score (RS) was calculated following the normalized expression of each candidate variable and its matched regression coefficient. The formula was constructed as follows: RS = *e*^sum (each variable's expression × corresponding regression coefficient)^. HCC patients were stratified into high- and low-risk subgroups in line with the median value of the RS. Overall survival between two subgroups was compared with survminer package. Uni- and multivariate cox regression models were established for assessing the predictive independency of the gene signature through adjustment of clinicopathological parameters (age, gender, grade, and stage). Time-dependent receiver-operating characteristic (ROC) curve analyses were conducted for evaluating the predictive power of the gene signature with survivalROC package. The CCP-derived gene signature was externally verified in the LIRI-JP cohort.

### 2.6. Establishment of a Prognostic Nomogram

Nomogram was established in TCGA-LIHC cohort with rms package. The 1-, 3-, and 5-year overall survival was assessed through total points, sum points of stage, and RS. The predictive performance of this nomogram was verified by ROC, calibration, and decisive curves.

### 2.7. Assessment of Tumor Microenvironment (TME)

Estimation of STromal and Immune cells in MAlignant Tumours using Expression data (ESTIMATE; https://sourceforge.net/projects/estimateproject/) method [21] was adopted for estimating the fractions of stromal and immune cells in TCGA-LIHC cohort. Through combination of stromal and immune scores, tumor purity was inferred. The ssGSEA algorithm was utilized for quantifying the relative abundance of 28 tumor-infiltrating immune cells in TCGA-LIHC cohort. The marker genes of immune cells were obtained from Charoentong et al. [22, 23]. The enrichment score was determined, which represented the infiltration of immune cells in HCC specimens. The mRNA expression of human leukocyte antigen (HLA) genes and immune checkpoints was analyzed in HCC samples.

### 2.8. Quantifying the Activity of Known Signaling Pathways

We collected the gene sets of IFN-*γ* signature, APM signal, base excision repair, cell cycle, DNA replication, Fanconi anemia pathway, homologous recombination, microRNAs in cancer, mismatch repair, nucleotide excision repair, oocyte meiosis, p53 signaling pathway, progesterone-mediated oocyte maturation, proteasome, pyrimidine metabolism, spliceosome, systemic lupus erythematosus, and viral carcinogenesis from published literature [24–26]. Spearman correlation test was presented between RS and above biological pathways.

### 2.9. Prediction of Potential Therapeutic Agents

This study gathered drug sensitivity profiling of human cancer cell lines from the CTRP (https://portals.broadinstitute.org/ctrp) projects. The area under the curve (AUC) value was utilized for inferring drug sensitivity. Moreover, transcriptome data in Cancer Cell Line Encyclopedia (CCLE) database (https://portals.broadinstitute.org/ccle/) were applied for CTRP analysis [27].

### 2.10. Screening Small Molecule Compounds

RS-relevant genes with ∣fold − change | >1.5 and adjusted *p* < 0.05 were screened between high- and low-risk HCC specimens. The up- and downregulated RS-relevant genes were separately uploaded into the Connectivity map (CMap; http://portals.broadinstitute.org/cmap/) project [28]. Potential small molecular compounds with ∣enrichment score | >0.8 and *p* < 0.05 were discovered. Mechanism of action of these compounds were evaluated with mode-of-action (MoA) analyses.

### 2.11. Analysis of Chemotherapy Response

The response to chemotherapeutic agents was evaluated based on the Genomics of Drug Sensitivity in Cancer (GDSC, https://www.cancerrxgene.org/) project [29]. Through applying pRRophetic package, the half-maximal inhibitory concentration (IC50) values of doxorubicin, cisplatin, gemcitabine, and sorafenib were calculated in each HCC specimen [30].

### 2.12. Statistical Analysis

Statistical analysis was conducted with R language (version 4.0.2). Comparisons between two groups were carried out via Student's *t*-test or Wilcoxon test. Spearman correlation test was utilized for assessment of the correlation between parameters. Two-sided *p* < 0.05 indicated statistical significance.

## 3. Results

### 3.1. CCP Acts as a Dominant Risk Factor for HCC Prognosis among Cancer Hallmarks

**Figure 1:**
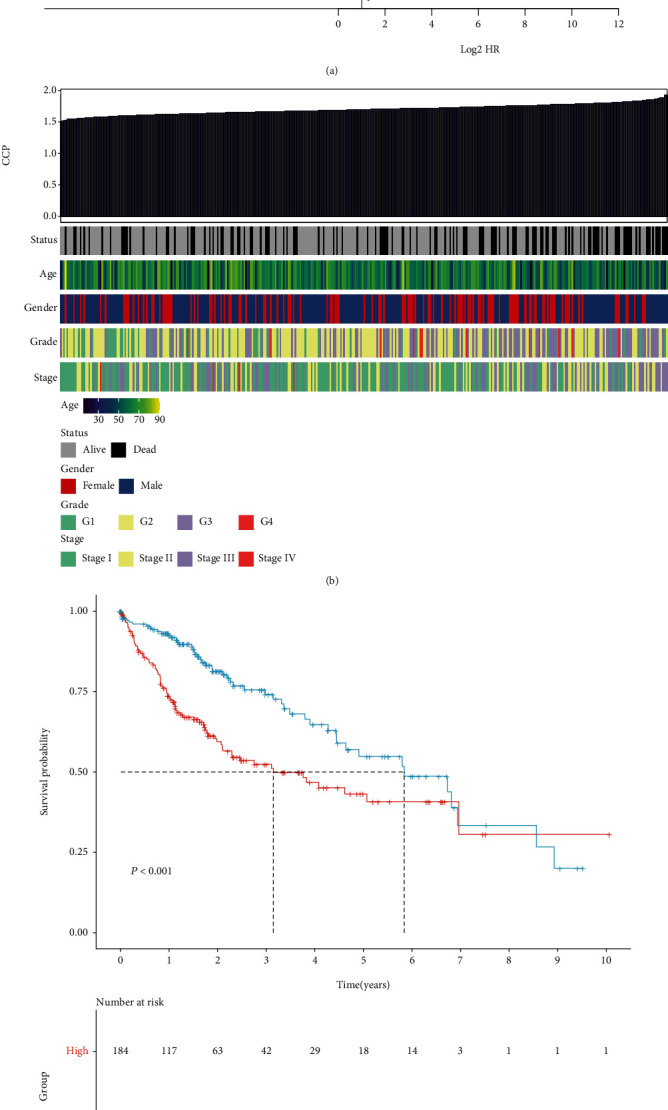
CCP acts as the most significant risk factor of HCC prognosis in TCGA-LIHC cohort. (a) Multivariate cox regression models that included glycolysis, CCP, age, gender, grade, and stage. (b) Heat map showing the associations between CCP score and clinicopathological parameters. (c) Prognostic analysis of HCC patients with high and low CCP score.

This study evaluated the prognostic significance of hallmarks of cancer defined by Hanahan and Weinberg. As shown in univariate cox regression models, angiogenesis (HR: 1.55 (0.45-5.29), *p* = 0.48), apoptosis (hazard ratio (HR): 0.64 (0.04-10.96), *p* = 0.76), EMT (HR: 2.01 (0.63-6.40), *p* = 0.24), hypoxia (HR: 13.17 (0.86-200.85), *p* = 0.06), inflammation (HR: 1.04 (0.26-4.15), *p* = 0.96), and immune infiltration (HR: 0.9997 (0.9996-1), *p* = 0.29) did not significantly affect the survival outcomes of HCC patients in TCGA-LIHC cohort. Meanwhile, DNA repair (HR: 1524850.08 (953.23-2439263968.30), *p* = 0.0002), glycolysis (HR: 3325.14 (67.71-163303.93), *p* < 0.0001), stemness (HR: 705.26 (35.39-14055.11), *p* < 0.0001), and CCP (HR: 439.59 (44.74-4319.26), *p* < 0.0001) were significant risk factors of HCC prognosis. Further multivariate cox regression models showed that glycolysis (HR: 447.32 (4.76-42064.25), *p* = 0.008) and CCP (HR: 63.19 (1.33-3001.70), *p* = 0.04) not DNA repair (HR: 993.12 (0.08-13051084.68), *p* = 0.15) and stemness (HR: 0.30 (0.002-41.35), *p* = 0.63) were independent risk factors for HCC. By adjusting clinicopathological parameters, we observed that, in addition to stage (HR: 1.44 (1.17-1.78), *p* = 0.0006) and age (HR: 1.01 (1.002-1.03), *p* = 0.03), CCP (HR: 98.54 (7.10-1366.81), *p* = 0.0006) was the only significant risk factor of HCC prognosis among hallmarks of cancer according to multivariate cox regression models (Figure 1(a)). The ssGSEA score was used to quantify CCP level in each HCC specimen.  Figure 1(b) visualized the associations of CCP score and common clinicopathological parameters. Survival analysis uncovered that high CCP score was in relation to unfavorable clinical outcomes of HCC patients (Figure 1(c)). Above findings suggested that CCP acted as a dominant risk factor for HCC prognosis among cancer hallmarks.

### 3.2. Identification of CCP-Relevant Genes in HCC

**Figure 2:**
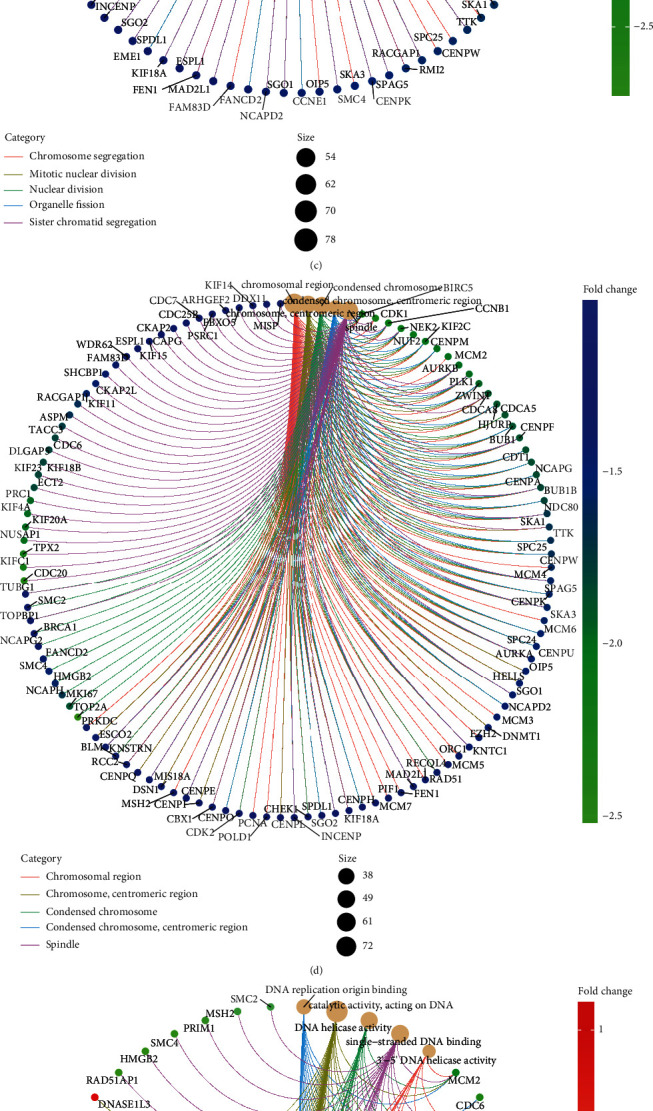
Identification of CCP-relevant genes in HCC and their biological functions. (a) Volcano plots showing CCP-relevant genes between high and low CCP score groups in HCC specimens from TCGA-LIHC cohort. (b) Heat map visualizing the mRNA expression of CCP-relevant genes in HCC patients with high and low CCP score. (c–e) The networks showing the significant biological process, cellular component, and molecular function enriched by CCP-relevant genes. (f) The gene-pathway network visualizing the KEGG pathways involved in CCP-relevant genes.

In total, 549 CCP-relevant genes were identified across HCC specimens in TCGA-LIHC cohort (Figures 2(a) and 2(b)). The detailed information was listed in Supplementary table 3. Biological functions of these 549 CCP-relevant genes were explored in depth. In Figure 2(c), biological processes of chromosome segregation, mitotic nuclear division, nuclear division, organelle fission, and sister chromatid segregation were distinctly regulated by the 549 CCP-relevant genes. Furthermore, the genes mainly participated in modulating the cellular components of chromosomal region, chromosome, centromeric region, condensed chromosome, condensed chromosome, centromeric region, and spindle (Figure 2(d)). They had the molecular functions of 3′-5′ DNA helicase activity, catalytic activity, acting on DNA, DNA helicase activity, DNA replication origin binding, and single-stranded DNA binding (Figure 2(e)). In Figure 2(f), cell cycle, DNA replication, drug metabolism-cytochrome P450, metabolism of xenobiotics by cytochrome P450, and retinol metabolism were distinctly modulated by the CCP-relevant genes.

### 3.3. Construction of a CCP-Derived Prognostic Gene Signature in HCC

**Figure 3:**
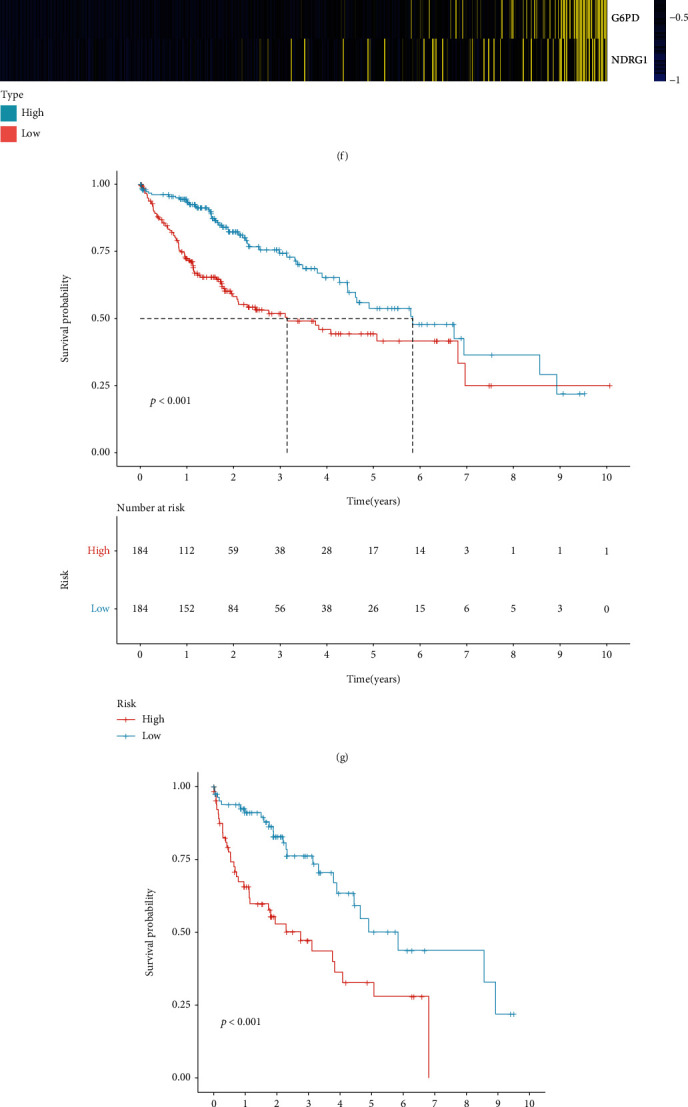
Construction of a CCP-derived prognostic gene signature for HCC in TCGA-LIHC cohort. (a) The change in trajectory of candidate variables. The *x*-axis represented the log (lambda) of variables, and the *y*-axis represented the coefficient of variables. (b) Partial likelihood deviance corresponding to each lambda. (c) The interactions between six candidate genes in the LASSO model. (d) Distribution of CCP-derived RS in each HCC patient. The high- and low-risk groups were separated in line with the median of RS (dotted line). (e) Distribution of survival status in high- and low-risk groups. (f) Heat map visualizing the mRNA expression of six candidate variables in high- and low-risk groups. Yellow, upregulation and blue, downregulation. (g) Prognostic analyses of HCC patients with high and low RS in TCGA-LIHC cohort. (h–o) Comparison of survival outcomes of HCC patients with high and low RS in each subgroup of age ≥ 65 and age < 65, female and male, G1-2 and G3-4, and stages I-II and stages III-IV.

Through univariate cox regression models, we identified 374 prognostic CCP-relevant genes for HCC in TCGA-LIHC cohort (Supplementary table 4). We adopted glmnet package to establish LASSO Cox regression model.  Figure 3(a) depicted the change in trajectory of above prognostic CCP-relevant genes, suggesting that more independent variables possessed regression coefficients approaching zero as *λ* gradually increased. Furthermore, tenfold crossverification was presented to establish the prognostic model. The confidence interval corresponding to each was shown in Figure 3(b). Finally, six candidate genes were included in the model, and the formula was as follows: RS = KIF20A expression∗0.0472314086601818 + CDCA8 expression∗0.063151035472353 + KPNA2 expression∗0.0550979279942481 + G6PD expression∗0.0909997485900923 + NDRG1 expression∗0.0336641751731575 + EPS8L3 expression∗0.00659414276835094. As depicted in Figure 3(c), there were significantly positive interactions between six candidate genes. In line with the median value of RS, HCC patients were stratified into high- and low-risk groups (Figure 3(d)). More dead patients were found in high-risk group (Figure 3(e)). With the increase in RS, KIF20A, CDCA8, KPNA2, G6PD, NDRG1, and EPS8L3 expression was gradually increased (Figure 3(f)). Survival difference was evaluated between two groups. In Figure 3(g), we observed that low-risk patients possessed the distinct survival advantage. The prognostic significance of RS was further investigated by subgroup analyses. We stratified HCC patients into different subgroups in line with clinicopathological parameters. Our data suggested that patients with high RS had poorer clinical outcomes in comparison to those with low RS in each subgroup of age ≥ 65 and age < 65 (Figures 3(h) and 3(i)), female and male (Figures 3(j) and 3(k)), G1-2 and G3-4 (Figures 3(l) and 3(m)), and stages I-II and stages III-IV (Figures 3(n) and 3(o)).

### 3.4. The CCP-Derived Gene Signature is a Robust Prognostic Predictor of HCC

**Figure 4:**
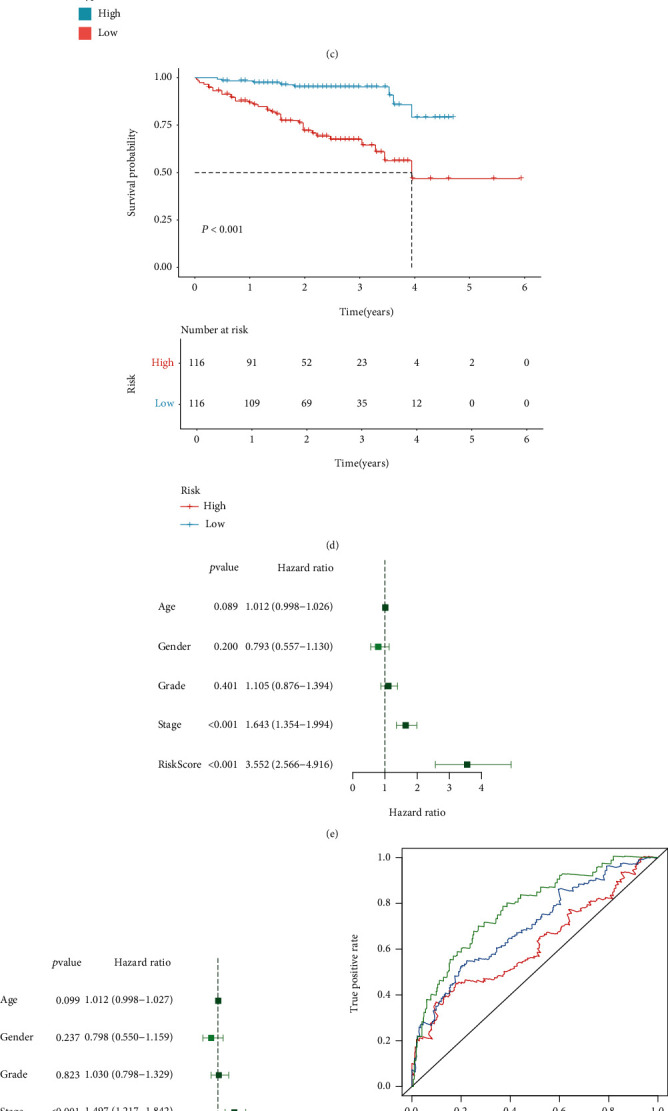
The CCP-derived gene signature is a robust prognostic predictor of HCC. (a) Distribution of CCP-derived RS across HCC patients in the LIRI-JP cohort. The high- and low-risk groups were separated in line with the median of RS (dotted line). (b) Distribution of survival status in high- and low-risk groups in the LIRI-JP cohort. (c) Heat map visualizing the mRNA expression of six candidate variables in high- and low-risk groups the LIRI-JP cohort. Yellow, upregulation and blue, downregulation. (d) Prognostic analyses of HCC patients with high and low RS in the LIRI-JP cohort. (e, f) Uni- and multivariate cox regression models of CCP-derived RS and clinicopathological parameters in TCGA-LIHC cohort. (g) ROC curves at 1-, 3-, and 5-year survival in TCGA-LIHC cohort. (h) ROC curves at 1-, 3-, and 5-year survival in the LIRI-JP cohort.

The predictive efficacy of the CCP-derived gene signature was externally verified in the LIRI-JP cohort. With the median of RS, HCC patients were stratified into two groups (Figure 4(a)). There were more dead cases in high-risk group (Figure 4(b)). Consistently, increased expression of KIF20A, CDCA8, KPNA2, G6PD, NDRG1, and EPS8L3 was found in high-risk patients (Figure 4(c)). Prognostic analyses uncovered the prominent survival advantage of high-risk patients (Figure 4(d)). Univariate cox regression models showed that stage and CCP-derived RS were in relation to HCC prognosis in TCGA-LIHC cohort (Figure 4(e)). As shown in multivariate cox regression models, stage and CCP-derived RS were both independent prognostic indicators of HCC (Figure 4(f)). The AUC values at 1-, 3-, and 5-year survival were 0.776, 0.697, and 0.619 in TCGA-LIHC cohort (Figure 4(g)). Also, in the LIRI-JP cohort, the AUC values at 1-, 3-, and 5-year survival were 0.779, 0.803, and 0.762 (Figure 4(h)). Above findings were indicative of the well-predictive performance of the CCP-derived RS in HCC prognosis.

### 3.5. Establishment of a Reliable Nomogram for HCC Prognosis

**Figure 5:**
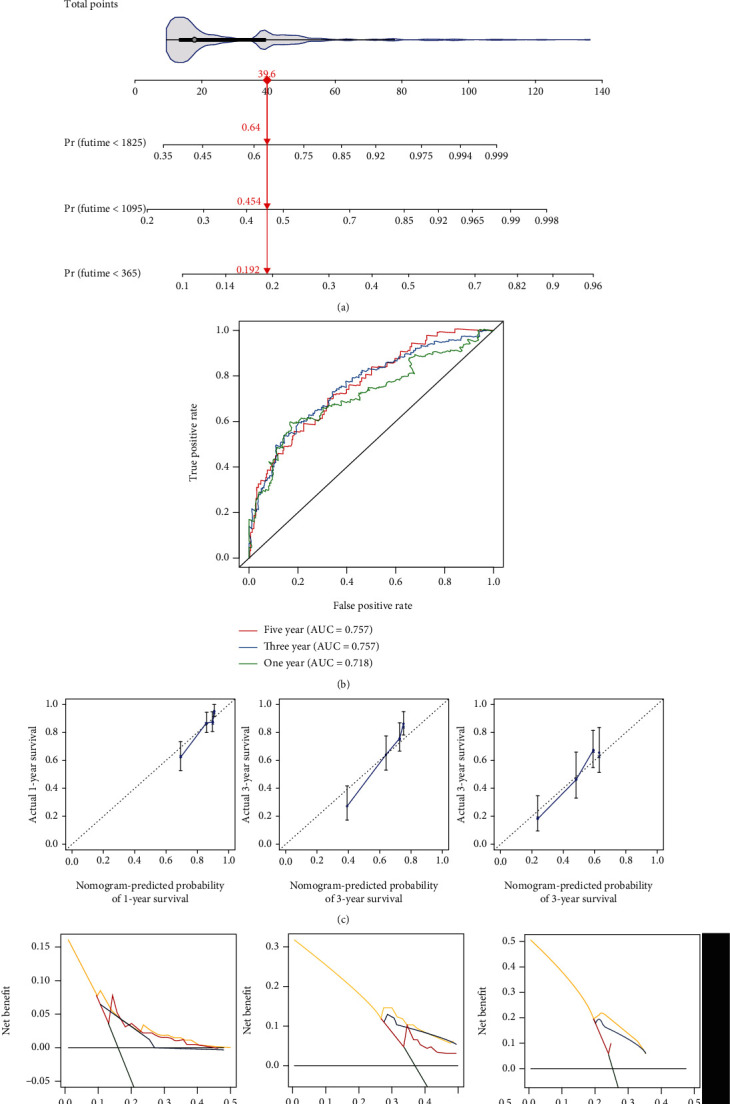
Establishment of a reliable nomogram for HCC prognosis in TCGA-LIHC cohort. (a) The nomogram that contained CCP-derived RS and stage for prediction of 1-, 3-, and 5-year survival probabilities of HCC. (b) ROC curves for verifying the predictive performance of this nomogram. (c) Comparison of nomogram-estimated and actual 1-, 3-, and 5-year survival probabilities of HCC. (d) Comparison of net benefit among none, all, stage, CCP-derived RS, and nomogram.

Two independent prognostic factors (CCP-derived RS and stage) were included for construction of a nomogram in TCGA-LIHC cohort (Figure 5(a)). Each level of RS or stage was assigned one score, and the total score was counted through summing up the scores in each patient. The 1-, 3-, and 5-year survival probabilities were determined by the function conversion relationships of total scores. The AUCs of this nomogram at 1-, 3-, and 5-year survival were 0.718, 0.757, and 0.757, indicative of the excellent predictive performance (Figure 5(b)). The calibration curves showed the high consistency between the nomogram-estimated 1-, 3-, and 5-year clinical outcomes and actual observations (Figure 5(c)). Decisive curves were conducted for evaluation of the guiding implications of the nomogram for clinical application. Our data demonstrated that the nomogram was the best for prediction of clinical outcomes of HCC (Figure 5(d)).

### 3.6. CCP-Derived Prognostic Gene Signature is in Relation to TME and Carcinogenic Pathway of HCC

**Figure 6:**
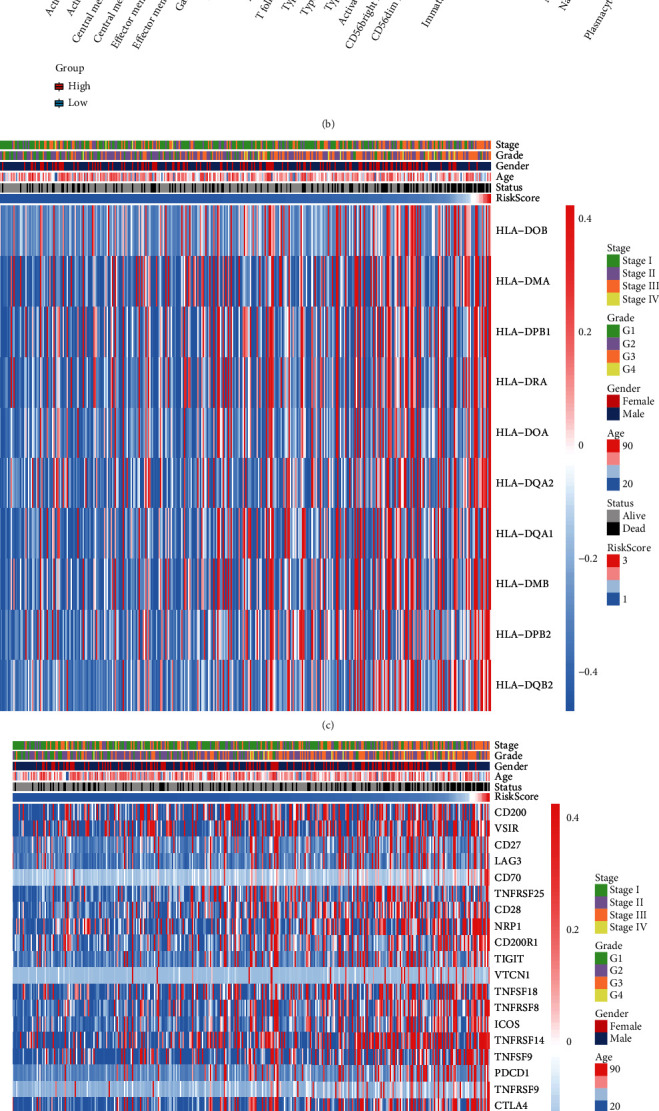
CCP-derived prognostic gene signature is in relation to TME and carcinogenic pathway of HCC in TCGA-LIHC cohort. (a) Comparison of immune score, stromal score, and tumor purity in high- and low-risk HCC patients with ESTIMATE algorithm. (b) Comparison of the infiltration levels of tumor-infiltrating immune cells in high- and low-risk HCC patients via ssGSEA method. ^∗^*p* < 0.05; ^∗∗^*p* < 0.01; ^∗∗∗^*p* < 0.001. (c) Heat map visualizing the mRNA expression of HLA genes in each HCC specimen and their relationships to CCP-derived RS. (d) Heat map showing the mRNA expression of immune checkpoints in each HCC sample as well as their relationships to CCP-derived RS. (e) Association between CCP-derived RS and known biological pathways.

ESTIMATE computational method was utilized for inferring the overall infiltration levels of immune and stromal cells. We observed no significant difference in immune score or tumor purity between high- and low-risk HCC patients (Figure 6(a)). Low-risk patients displayed significantly increased stromal score in comparison to high-risk patients. In Figure 6(b), activated CD4+ T cell, central memory CD4+ T cell, memory B cell, type 17 helper cell, type 2 helper cell, and activated dendritic cell had increased infiltration levels in high-risk than low-risk HCC specimens. Reduced infiltration levels of effector memory CD8+ T cell, CD56 bright natural killer cell, eosinophil, and mast cell were found in high-risk compared to low-risk HCC samples. Furthermore, high RS was in relation to increased mRNA expression of HLA family, including HLA-DOB, HLA-DMA, HLA-DPB1, HLA-DRA, HLA-DOA, HLA-DQA2, HLA-DQA1, HLA-DMB, HLA-DPB2, and HLA-DQB2 (Figure 6(c)). We also compared the mRNA expression of immune checkpoints between high- and low-risk HCC specimens. We observed that CD200, VSIR, CD27, LAG3, CD70, TNFRSF25, CD28, NRP1, CD200R1, TIGIT, VTCN1, TNFSF18, TNFRSF18, ICOS, TNFRSF14, TNFSF9, PDCD1, TNFRSF9, CTLA4, CD86, LGALS9, TNFRSF18, HAVCR2, LAIR1, TNFRSF4, TNFSF15, HHLA2, CD80, TNFSF4, and CD276 expression was gradually increased with the increase of RS (Figure 6(d)). Association between RS and known biological pathways was evaluated across HCC samples. In Figure 6(e), RS displayed positive correlation to carcinogenic pathway activation including IFN-*γ* signature, APM signal, base excision repair, cell cycle, DNA replication, Fanconi anemia pathway, homologous recombination, microRNAs in cancer, mismatch repair, nucleotide excision repair, oocyte meiosis, p53 signaling pathway, progesterone-mediated oocyte maturation, proteasome, pyrimidine metabolism, spliceosome, systemic lupus erythematosus, and viral carcinogenesis.

### 3.7. Prediction of Potential Therapeutic Compounds against HCC Based on CCP-Derived Gene Signature

**Figure 7:**
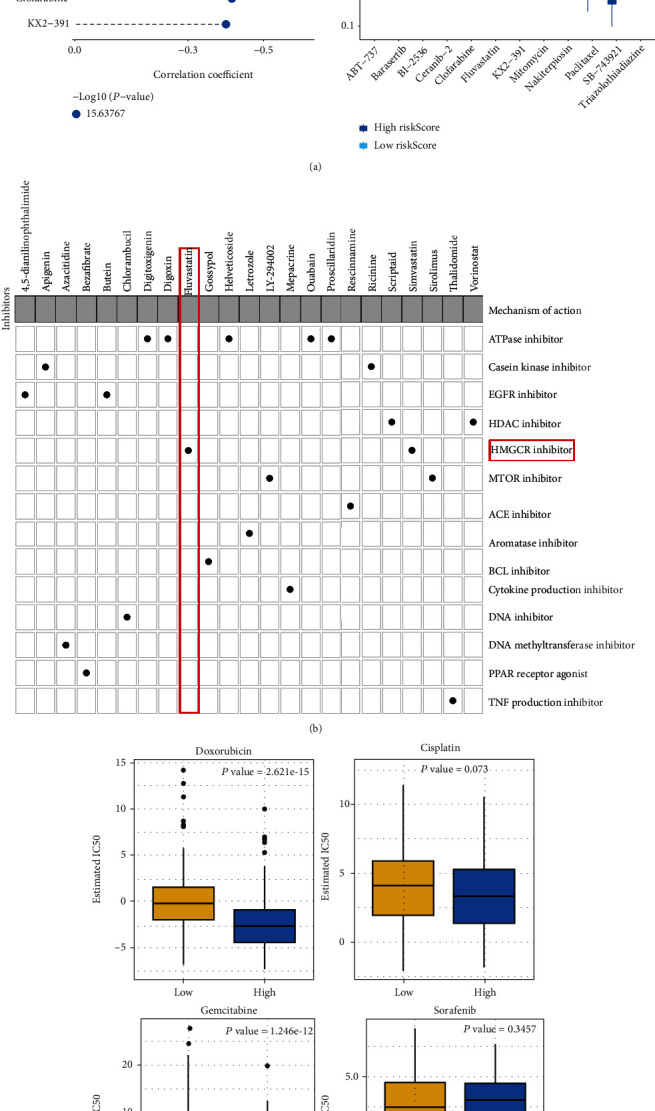
Prediction of potential therapeutic compounds as well as assessment of chemosensitivity based on CCP-derived gene signature in TCGA-LIHC cohort. (a) Spearman correlation between CTRP-derived compounds and CCP-derived RS as well as comparison of estimated AUC value between high- and low-risk HCC samples. ^∗∗∗^*p* < 0.001. (b) Shared mechanisms of action of small molecular compounds derived from CMap database. (c) Comparison of the estimated IC50 value of doxorubicin, cisplatin, gemcitabine, and sorafenib in high- and low-risk HCC samples.

This study further discovered promising therapeutic agents against HCC based on the CCP-derived gene signature in TCGA-LIHC cohort. In Figure 7(a), 12 CTRP-derived compounds were identified, including paclitaxel (*r* = −0.48), BI-2536 (*r* = −0.45), triazolothiadiazine (*r* = −0.48), ABT-737 (*r* = −0.44), nakiterpiosin (*r* = −0.51), mitomycin (*r* = −0.50), barasertib (*r* = −0.43), ceranib-2 (*r* = −0.54), SB-743921 (*r* = −0.52), clofarabine (*r* = −0.42), fluvastatin (*r* = −0.62), and KX2-391 (*r* = −0.40). AUC values of above compounds were compared between high- and low-risk HCC patients. We observed that high-risk patients had prominently reduced AUC values of each compound. This indicated that high-risk patients were more likely to respond to above compounds. Small molecular compounds were also predicted by CMap project. In total, 33 small molecular compounds with ∣enrichment score | >0.8 and *p* < 0.05 were identified (Table 1). Shared mechanisms of action of above compounds were shown in Figure 7(b). We observed that digitoxigenin, digoxin, helveticoside, ouabain, and proscillaridin shared the ATPase inhibitor; apigenin and ricinine shared casein kinase inhibitor; 4, 5-dianilinophthalimide and butein shared EGFR inhibitor; scriptaid and vorinostat shared HDAC inhibitor; fluvastatin and simvastatin shared HMGCR inhibitor; and LY-294002 and sirolimus shared mTOR inhibitor. Fluvastatin was predicted both in CTRP and CMap projects, indicative of the potential as a therapeutic agent against HCC.

### 3.8. CCP-Derived Gene Signature Predicts Chemotherapeutic Response

The response to chemotherapeutic agents including doxorubicin, cisplatin, gemcitabine, and sorafenib in each HCC sample was evaluated in TCGA-LIHC cohort. We compared the IC50 value of above agents in high- and low-risk HCC patients. As a result, high-risk patients had markedly reduced IC50 values of doxorubicin and gemcitabine than those with low-risk, suggesting that high RS was indicative of increased sensitivity to doxorubicin and gemcitabine (Figure 7(c)). However, no significant difference was investigated between high- and low-risk HCC patients. Thus, CCP-derived prognostic gene signature might be applied for predicting the response to doxorubicin and gemcitabine.

### 3.9. CCP-Derived Gene Signature Acts as a Reliable Prognostic Indicator Independent of Genetic Mutation

**Figure 8:**
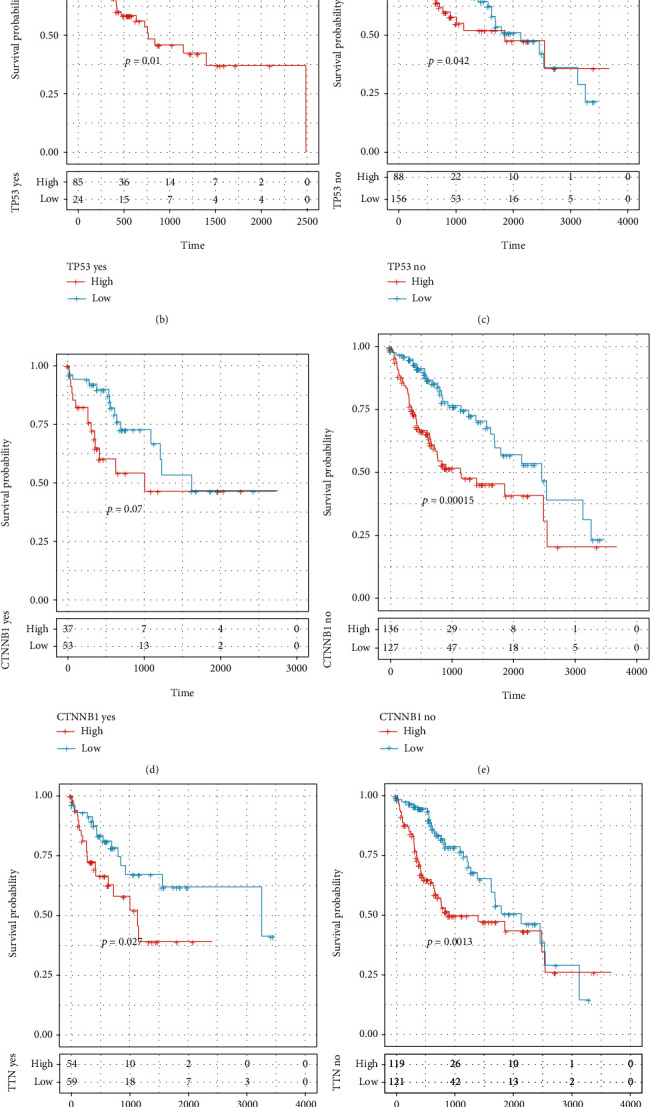
CCP-derived gene signature acts as a reliable prognostic indicator independent of genetic mutation. (a) Landscape of somatic mutation of HCC specimens. (b–i) Survival analysis of high- and low-risk HCC patients in each subgroup (TP53 mutation and nonmutation subgroups; CTNNB1 mutation and nonmutation subgroups, TTN mutation and nonmutation subgroups, and MUC16 mutation and nonmutation subgroups).

Further analysis revealed that 84.62% occurred somatic mutation across 364 HCC samples (Figure 8(a)). TP53 was the most frequent mutation (30%), followed by CTNNB1 (25%), TTN (24%), and MUC16 (14%). Missense mutation was the main type of mutation. HCC samples were stratified into different subgroups according to whether above genes occurred mutation. We observed that high-RS patients displayed poorer clinical outcomes in comparison to low-RS patients regardless of whether TP53, CTNNB1, TTN, and MUC16 were mutated (Figures 8(b)–8(i)). This indicated that CCP-derived gene signature acted as a reliable prognostic indicator independent of genetic mutation.

## 4. Discussion

Nowadays, bioinformatics analysis has become an important tool for cancer research. Herein, our study suggested that CCP acted as a dominant risk factor for HCC prognosis among hallmarks of cancer. The LASSO computational method is a shrinkage estimate, which may be utilized for constructing a penalty function as well as obtaining a relatively refined model, where several coefficients may be shrunk, and several are set to zero. Hence, this model possesses the advantage of subset shrinkage and represents a biased estimate, which may process multiple collinear data. It is capable of estimating parameters and selecting variables, thereby solving the problem of multiple collinearities in regression analyses. Thus, we adopted LASSO method to construct a robust CCP-derived gene signature for HCC prognosis.

This signature contained KIF20A, CDCA8, KPNA2, G6PD, NDRG1, and EPS8L3. KIF20A dysregulation may be independently predictive of unfavorable clinical outcomes and recurrence of HCC patients [31]. KIF20A downregulation may decrease the proliferation and induce the G1 arrest of HCC cells [32]. CDCA8 is in relation to unfavorable stage and survival outcomes of HCC [33]. CDCA8 inhibition weakens HCC growth and stemness through restoring ATF3 and inactivating AKT/*β*-catenin axis [34]. KPNA2 upregulation accelerates HCC progression via enhancement of migration [35] and proliferation and is indicative of undesirable prognosis [36]. KPNA2 dysregulation is also in relation to early recurrence for patients with small HCC following hepatectomy [37]. G6PD upregulation leads to migration and invasion of HCC cells through enhancing EMT [38]. It can inhibit ferroptosis in HCC through reducing cytochrome P450 oxidoreductase [39]. Hypoxia-mediated NDRG1 upregulation modulates apoptotic levels via driving mitochondrial fission in HCC [40]. NDRG1 acts as a predictor of metastases, relapse, and undesirable prognosis in HCC [41]. EPS8L3 upregulation enhances HCC proliferation through inhibition of the transactivity of FOXO1 [42]. It can also induce HCC proliferation and metastases through modulation of EGFR dimerization and internalization [43]. Also, we observed that the CCP-derived gene signature exhibited positive correlation to carcinogenic pathway activation. Hence, each variable in the CCP-derived gene signature acts as an oncogene during HCC progression.

Our prognostic analysis uncovered that the CCP-derived gene signature can be independently predictive of HCC patients' clinical outcomes. Patients with high RS indicated unfavorable prognosis. ROC curves at 1-, 3-, and 5-year survival confirmed the well predictive efficacy. The external verification demonstrated the clinical applicability of this CCP-derived gene signature. A recent study has proposed a novel cell cycle-relevant prognostic model for endometrial cancer [44]. This study is the first to construct the CCP-derived gene signature in HCC prognosis. Through integration of stage and CCP-derived signature, we developed a nomogram. Following verification of ROC, calibration, and decisive curves, this nomogram possessed the potential as a clinical tool for predicting HCC prognosis.

Recently, immune checkpoint inhibitors like PD-1, PD-L1, and CTLA-4 antibodies display favorable therapeutic effects against advanced HCC [45]. But current immune-based therapies induce durable response in a minority of HCC subjects. Due to the indispensable role of TME in HCC pathogenesis, it is of significance to uncover the underlying mechanism shaping the unique TME of HCC [46]. Our findings suggested that high-risk HCC possessed the increased infiltration levels of activated CD4+ T cell, central memory CD4+ T cell, memory B cell, type 17 helper cell, type 2 helper cell, and activated dendritic cell as well as had the reduced infiltration levels of effector memory CD8+ T cell, CD56 bright natural killer cell, eosinophil, and mast cell. Furthermore, most of HLA genes and immune checkpoints displayed the significant upregulation in high-risk patients. Hence, the CCP-derived gene signature was in relation to TME reshaping.

Combining CTRP and CMap analyses, we finally identified fluvastatin as a promising therapeutic agent. Evidence suggests that fluvastatin suppresses apoptosis of HCC cells [47]. High RS had increased sensitivity to doxorubicin and gemcitabine for HCC patients. This indicated that CCP-derived gene signature might be utilized for prediction of response to doxorubicin and gemcitabine. Furthermore, we conducted the genetic mutation landscape of HCC. Our data suggested that the CCP-derived gene signature acted as a reliable prognostic predictor independent of genetic mutation. However, several limitations of this study should be pointed out. Although the CCP-derived gene signature has been verified externally, the predictive performance should be observed in a prospective cohort. Moreover, each gene in the CCP-derived gene signature will be validated through in vivo or in vitro experiments.

## 5. Conclusion

Collectively, our study proposed the CCP-derived gene signature for HCC prognosis. Following verification, this signature acted as a robust prognostic predictor for HCC. Also, a reliable nomogram including the CCP-derived gene signature and stage was developed as a promising clinical tool. Further analysis uncovered that the CCP-derived gene signature was in relation to TME reshaping and response to doxorubicin and gemcitabine. Based on the CCP-derived gene signature, fluvastatin was confirmed as a therapeutic agent against HCC. Despite this, the therapeutic effects of fluvastatin required to be verified in more experiments. Moreover, the predictive efficacy of the CCP-derived gene signature in HCC clinical outcomes will be verified in prospective cohorts.

## Figures and Tables

**Table 1:** Identification of small molecular compounds against HCC by CMap database.

CMap name	Mean	*n*	Enrichment	*p*	Specificity	Percent nonnull
DL-thiorphan	-0.847	2	-0.933	0.00926	0.0227	100
Sanguinarine	-0.864	2	-0.929	0.01036	0.0188	100
Apigenin	-0.833	4	-0.884	0.00042	0.0163	100
Quinostatin	-0.768	2	-0.882	0.02827	0.1985	100
Menadione	-0.788	2	-0.871	0.03314	0.046	100
Blebbistatin	-0.753	2	-0.862	0.03825	0.0608	100
8-Azaguanine	-0.907	4	-0.853	0.00092	0.014	100
Luteolin	-0.773	4	-0.849	0.00095	0.0114	100
Alsterpaullone	-0.771	3	-0.843	0.00773	0.1143	100
Thioguanosine	-0.785	4	-0.837	0.00125	0.0141	100
0297417-0002B	-0.759	3	-0.816	0.0124	0.0738	100
Bisacodyl	-0.736	4	-0.814	0.00221	0.0242	100
Lasalocid	0.663	4	0.801	0.00298	0.0245	100
Cefamandole	0.617	4	0.805	0.00269	0.0104	100
Ikarugamycin	0.65	3	0.817	0.0121	0.0389	100
Gly-His-Lys	0.738	3	0.822	0.0112	0.0449	100
Prestwick-691	0.711	3	0.831	0.00957	0.025	100
Heptaminol	0.745	5	0.842	0.00022	0.0121	100
Atracurium besilate	0.712	3	0.845	0.00723	0.0173	100
3-Acetamidocoumarin	0.728	4	0.853	0.0007	0	100
Thiamphenicol	0.746	5	0.855	0.00014	0.0124	100
Penbutolol	0.736	3	0.863	0.00481	0.017	100
Thapsigargin	0.674	3	0.871	0.00397	0.1401	100
Nadolol	0.737	4	0.872	0.00036	0.0055	100
Felbinac	0.733	4	0.875	0.00034	0.0117	100
Prestwick-1103	0.693	4	0.875	0.00034	0	100
Gentamicin	0.678	4	0.878	0.0003	0.0063	100
Biperiden	0.736	5	0.882	0.00006	0.0061	100
Prestwick-1082	0.713	3	0.883	0.00322	0.0158	100
Prestwick-692	0.769	4	0.913	0.00004	0.0061	100
Isoflupredone	0.874	3	0.94	0.0003	0.0052	100
Viomycin	0.861	4	0.948	0	0	100
Adiphenine	0.905	5	0.956	0	0	100

## Data Availability

The data used to support the findings of this study are included within the supplementary information files.

## Abbreviations

HCC: Hepatocellular carcinoma
TCGA: The Cancer Genome Atlas
ICGC: International Cancer Genome Consortium
CCP: Cell cycle progression
EMT: Epithelial-mesenchymal transition
ssGSEA: Single sample gene set enrichment analysis
GSVA: Gene set variation analysis
GO: Gene Ontology
KEGG: Kyoto Encyclopedia of Genes and Genomes
LASSO: Least absolute shrinkage and selection operator
RS: Risk score
ROC: Receiver-operating characteristic
TME: Tumor microenvironment
ESTIMATE: Estimation of STromal and Immune cells in MAlignant Tumours using Expression data
HLA: Human leukocyte antigen
AUC: Area under the curve
CCLE: Cancer Cell Line Encyclopedia
CMap: Connectivity map
MoA: Mode-of-action
GDSC: Genomics of Drug Sensitivity in Cancer
IC50: Half-maximal inhibitory concentration.

## Supplementary Materials

Supplementary Materials Supplementary table 1. Clinical information of HCC patients in TCGA-LIHC cohort. Supplementary table 2. The gene sets of hallmarks of cancer. Supplementary table 3. 549 CCP-relevant genes in TCGA-LIHC cohort. Supplementary table 4. Prognostic CCP-relevant genes in TCGA-LIHC cohort.

## References

[1] Craig A. J., von Felden J., Garcia-Lezana T., Sarcognato S., Villanueva A. (2020). Tumour evolution in hepatocellular carcinoma. *Nature Reviews Gastroenterology & Hepatology*.

[2] Tang B., Zhu J., Li J. (2020). The ferroptosis and iron-metabolism signature robustly predicts clinical diagnosis, prognosis and immune microenvironment for hepatocellular carcinoma. *Cell Communication and Signaling*.

[3] Hong W., Liang L., Gu Y. (2020). Immune-related lncRNA to construct novel signature and predict the immune landscape of human hepatocellular carcinoma. *Molecular Therapy - Nucleic Acids*.

[4] Liang J. Y., Wang D. S., Lin H. C. (2020). A novel ferroptosis-related gene signature for overall survival prediction in patients with hepatocellular carcinoma. *International Journal of Biological Sciences*.

[5] Liu Y., Zhang X., Zhang J., Tan J., Li J., Song Z. (2020). Development and validation of a combined ferroptosis and immune prognostic classifier for hepatocellular carcinoma. *Frontiers in Cell and Developmental Biology*.

[6] Xiao H., Wang B., Xiong H. X. (2021). A novel prognostic index of hepatocellular carcinoma based on immunogenomic landscape analysis. *Journal of Cellular Physiology*.

[7] Hanahan D., Weinberg R. A. (2011). Hallmarks of cancer: the next generation. *Cell*.

[8] Evan G. I., Vousden K. H. (2001). Proliferation, cell cycle and apoptosis in cancer. *Nature*.

[9] Cooperberg M. R., Simko J. P., Cowan J. E. (2013). Validation of a cell-cycle progression gene panel to improve risk stratification in a contemporary prostatectomy cohort. *Journal of Clinical Oncology*.

[10] Mosley J. D., Keri R. A. (2008). Cell cycle correlated genes dictate the prognostic power of breast cancer gene lists. *BMC Medical Genomics*.

[11] Cuzick J., Swanson G. P., Fisher G. (2011). Prognostic value of an RNA expression signature derived from cell cycle proliferation genes in patients with prostate cancer: a retrospective study. *Lancet Oncology*.

[12] West J. M., Ma D., Mott S. L., Brown J. A. (2020). Cell cycle progression score has potential prognostic value for stage T1 renal cell carcinomas. *Urologic Oncology*.

[13] Wang Z., Jensen M. A., Zenklusen J. C. (2016). A practical guide to The Cancer Genome Atlas (TCGA). *Methods in Molecular Biology*.

[14] Zhang J., Bajari R., Andric D. (2019). The international cancer genome consortium data portal. *Nature Biotechnology*.

[15] Mayakonda A., Lin D. C., Assenov Y., Plass C., Koeffler H. P. (2018). Maftools: efficient and comprehensive analysis of somatic variants in cancer. *Genome Research*.

[16] Liberzon A., Birger C., Thorvaldsdóttir H., Ghandi M., Mesirov J. P., Tamayo P. (2015). The Molecular Signatures Database Hallmark Gene Set Collection. *Cell Systems*.

[17] Hänzelmann S., Castelo R., Guinney J. (2013). GSVA: gene set variation analysis for microarray and RNA-seq data. *BMC Bioinformatics*.

[18] Newman A. M., Liu C. L., Green M. R. (2015). Robust enumeration of cell subsets from tissue expression profiles. *Nature Methods*.

[19] Yu G., Wang L. G., Han Y., He Q. Y. (2012). clusterProfiler: an R package for comparing biological themes among gene clusters. *OMICS: A Journal of Integrative Biology*.

[20] Engebretsen S., Bohlin J. (2019). Statistical predictions with glmnet. *Clinical Epigenetics*.

[21] Yoshihara K., Shahmoradgoli M., Martínez E. (2013). Inferring tumour purity and stromal and immune cell admixture from expression data. *Nature Communications*.

[22] Barbie D. A., Tamayo P., Boehm J. S. (2009). Systematic RNA interference reveals that oncogenic _KRAS_ -driven cancers require TBK1. *Nature*.

[23] Charoentong P., Finotello F., Angelova M. (2017). Pan-cancer immunogenomic analyses reveal genotype-immunophenotype relationships and predictors of response to checkpoint blockade. *Cell Reports*.

[24] Şenbabaoğlu Y., Gejman R. S., Winer A. G. (2016). Tumor immune microenvironment characterization in clear cell renal cell carcinoma identifies prognostic and immunotherapeutically relevant messenger RNA signatures. *Genome Biol*.

[25] Rosenberg J. E., Hoffman-Censits J., Powles T. (2016). Atezolizumab in patients with locally advanced and metastatic urothelial carcinoma who have progressed following treatment with platinum-based chemotherapy: a single-arm, multicentre, phase 2 trial. *Lancet*.

[26] Mariathasan S., Turley S. J., Nickles D. (2018). TGF*β* attenuates tumour response to PD-L1 blockade by contributing to exclusion of T cells. *Nature*.

[27] Ghandi M., Huang F. W., Jané-Valbuena J. (2019). Next-generation characterization of the Cancer Cell Line Encyclopedia. *Nature*.

[28] Lamb J., Crawford E. D., Peck D. (2006). The connectivity map: using gene-expression signatures to connect small molecules, genes, and disease. *Science*.

[29] Yang W., Soares J., Greninger P. (2012). Genomics of Drug Sensitivity in Cancer (GDSC): a resource for therapeutic biomarker discovery in cancer cells. *Nucleic Acids Research*.

[30] Geeleher P., Cox N., Huang R. S. (2014). pRRophetic: an R package for prediction of clinical chemotherapeutic response from tumor gene expression levels. *PLoS One*.

[31] Lu M., Huang X., Chen Y. (2018). AberrantKIF20Aexpression might independently predict poor overall survival and recurrence-free survival of hepatocellular carcinoma. *IUBMB Life*.

[32] Li X., Huang W., Huang W. (2020). Kinesin family members KIF2C/4A/10/11/14/18B/20A/23 predict poor prognosis and promote cell proliferation in hepatocellular carcinoma. *American Journal of Translational Research*.

[33] Li D., Ji Y., Guo J., Guo Q. (2021). Upregulated expression ofMTFR2as a novel biomarker predicts poor prognosis in hepatocellular carcinoma by bioinformatics analysis. *Future Oncology*.

[34] Jeon T., Ko M. J., Seo Y. R. (2021). Silencing CDCA8 suppresses hepatocellular carcinoma growth and stemness via restoration of ATF3 tumor suppressor and inactivation of AKT/*β*-catenin signaling. *Cancers*.

[35] Guo X., Wang Z., Zhang J. (2019). Upregulated KPNA2 promotes hepatocellular carcinoma progression and indicates prognostic significance across human cancer types. *Acta Biochimica et Biophysica Sinica*.

[36] Zan Y., Wang B., Liang L. (2019). MicroRNA-139 inhibits hepatocellular carcinoma cell growth through down-regulating karyopherin alpha 2. *Journal of Experimental & Clinical Cancer Research*.

[37] Jiang P., Tang Y., He L. (2014). Aberrant expression of nuclear KPNA2 is correlated with early recurrence and poor prognosis in patients with small hepatocellular carcinoma after hepatectomy. *Medical Oncology*.

[38] Lu M., Lu L., Dong Q. (2018). Elevated G6PD expression contributes to migration and invasion of hepatocellular carcinoma cells by inducing epithelial-mesenchymal transition. *Acta Biochimica et Biophysica Sinica*.

[39] Cao F., Luo A., Yang C. (2021). G6PD inhibits ferroptosis in hepatocellular carcinoma by targeting cytochrome P450 oxidoreductase. *Cell Signal*.

[40] Guo D. D., Xie K. F., Luo X. J. (2020). Hypoxia-induced elevated NDRG1 mediates apoptosis through reprograming mitochondrial fission in HCC. *Gene*.

[41] Cheng J., Xie H. Y., Xu X. (2011). NDRG1 as a biomarker for metastasis, recurrence and of poor prognosis in hepatocellular carcinoma. *Cancer Letters*.

[42] ZENG C. X., TANG L. Y., XIE C. Y. (2018). Overexpression of EPS8L3 promotes cell proliferation by inhibiting the transactivity of FOXO1 in HCC. *Neoplasma*.

[43] Xuan Z., Zhao L., Li Z. (2020). EPS8L3 promotes hepatocellular carcinoma proliferation and metastasis by modulating EGFR dimerization and internalization. *American journal of cancer research*.

[44] Liu J., Mei J., Li S., Wu Z., Zhang Y. (2020). Establishment of a novel cell cycle-related prognostic signature predicting prognosis in patients with endometrial cancer. *Cancer Cell International*.

[45] Zongyi Y., Xiaowu L. (2020). Immunotherapy for hepatocellular carcinoma. *Cancer Letters*.

[46] Ruf B., Heinrich B., Greten T. F. (2021). Immunobiology and immunotherapy of HCC: spotlight on innate and innate-like immune cells. *Cellular & Molecular Immunology*.

[47] al-Wahaibi L. H., al-Saleem M. S. M., Ahmed O. A. A. (2020). Optimized conjugation of fluvastatin to HIV-1 TAT displays enhanced pro-apoptotic activity in HepG2 cells. *International Journal of Molecular Sciences*.

