# Supplementary material for: A Cell Cycle Progression-Derived Gene Signature to Predict Prognosis and Therapeutic Response in Hepatocellular Carcinoma
Source: Dis Markers. 2021 Oct 21;2021:1986159. doi: 10.1155/2021/1986159 (PMC8553501; doi:10.1155/2021/1986159)
Supplement: Supplementary Materials — Supplementary table 1. Clinical information of HCC patients in TCGA-LIHC cohort. Supplementary table 2. The gene sets of hallmarks of cancer. Supplementary table 3. 549 CCP-relevant genes in TCGA-LIHC cohort. Supplementary table 4. Prognostic CCP-relevant genes in TCGA-LIHC cohort. [file 1986159.f1.zip › 1986159.f1/Supplementary table 1 (1).pdf]

Supplementary table 1. Clinical information of HCC patients in TCGA-LIHC cohort.

ID	futime	fustat	Age	Gender	Grade	Stage
TCGA-FV-A495-01A	1	0	51	Female	G2	Stage II
TCGA-ED-A7PZ-01A	6	0	61	Male	G2	Stage II
TCGA-ED-A7PX-01A	6	0	48	Female	G3	Stage II
TCGA-ED-A97K-01A	6	0	54	Male	G2	Stage III
TCGA-BC-A3KF-01A	8	0	66	Female	G2	Stage I
TCGA-DD-AACK-01A	9	0	70	Male	G2	Stage I
TCGA-DD-A4NR-01A	9	1	85	Female	G3	Stage I
TCGA-RC-A6M6-01A	9	0	75	Male	G3	Stage II
TCGA-FV-A496-01A	10	0	84	Female	G2	Stage I
TCGA-DD-A3A8-01A	11	1	75	Male	G2	Stage II
TCGA-DD-AADM-01A	12	1	58	Male	G3	Stage II
TCGA-FV-A4ZQ-01A	12	0	52	Male	G2	Stage I
TCGA-DD-A11B-01A	14	1	73	Male	G2	Stage I
TCGA-RC-A6M5-01A	15	0	20	Female	G2	Stage IV
TCGA-DD-AAC8-01A	16	1	72	Male	G3	Stage I
TCGA-EP-A2KC-01A	19	1	62	Male	G3	Stage I
TCGA-5C-AAPD-01A	20	0	61	Male	G1	Stage II
TCGA-BW-A5NO-01A	20	0	50	Male	G2	Stage III
TCGA-ZP-A9D1-01A	21	0	56	Female	G2	Stage III
TCGA-RC-A6M4-01A	22	0	74	Female	G2	Stage III
TCGA-T1-A6J8-01A	23	0	68	Male	G2	Stage II
TCGA-G3-AAV4-01A	27	1	83	Female	G1	Stage I
TCGA-WQ-A9G7-01A	30	0	71	Female	G3	Stage I
TCGA-GJ-A6C0-01A	31	1	75	Female	G2	Stage II
TCGA-BC-A10Z-01A	34	1	62	Female	G2	Stage I
TCGA-2Y-A9HA-01A	36	1	70	Male	G2	Stage II
TCGA-DD-A73E-01A	44	0	66	Male	G1	Stage I
TCGA-5R-AAAM-01A	46	1	65	Female	G2	Stage II
TCGA-UB-A7MD-01A	52	1	67	Male	G3	Stage I
TCGA-ED-A8O6-01A	56	1	50	Female	G3	Stage III
TCGA-G3-A7M9-01A	56	1	70	Male	G2	Stage III
TCGA-KR-A7K0-01A	65	1	65	Male	G1	Stage I
TCGA-G3-AAV6-01A	65	1	53	Female	G3	Stage III
TCGA-GJ-A9DB-01A	67	1	68	Male	G2	Stage I
TCGA-DD-A11A-01A	79	0	67	Male	G3	Stage I
TCGA-CC-5260-01A	87	1	61	Female	G1	Stage III
TCGA-HP-A5MZ-01A	91	1	78	Male	G2	Stage I
TCGA-BC-A10W-01A	91	1	50	Male	G3	Stage III
TCGA-QA-A7B7-01A	94	0	48	Male	G2	Stage II
TCGA-CC-5261-01A	97	1	44	Male	G2	Stage II
TCGA-CC-A1HT-01A	101	1	50	Male	G3	Stage III
TCGA-CC-5264-01A	102	1	71	Male	G2	Stage III
TCGA-CC-5262-01A	103	1	67	Male	G1	Stage III
TCGA-DD-AACL-01A	107	1	66	Female	G3	Stage I
TCGA-DD-AADF-01A	115	1	64	Female	G4	Stage I
TCGA-CC-5258-01A	129	1	48	Male	G2	Stage II
TCGA-CC-5263-01A	129	1	35	Male	G1	Stage III
TCGA-DD-AAD0-01A	137	0	73	Female	G2	Stage I
TCGA-CC-A8HT-01A	140	1	74	Male	G2	Stage III
TCGA-DD-AAE6-01A	141	0	59	Female	G2	Stage I
TCGA-BC-A112-01A	153	1	80	Male	G2	Stage I

TCGA-DD-AACX-01A	170	0	66	Male	G3	Stage II
TCGA-DD-A39Y-01A	171	1	67	Male	G3	Stage I
TCGA-DD-AACZ-01A	171	1	63	Female	G4	Stage I
TCGA-G3-A3CI-01A	180	0	71	Male	G2	Stage I
TCGA-DD-A1EI-01A	183	0	46	Male	G2	Stage I
TCGA-FV-A3R2-01A	194	1	75	Male	G2	Stage I
TCGA-DD-AACH-01A	195	1	69	Male	G3	Stage II
TCGA-CC-A9FS-01A	211	0	55	Male	G2	Stage II
TCGA-UB-A7MF-01A	214	1	56	Male	G2	Stage III
TCGA-CC-A7IE-01A	217	1	57	Male	G2	Stage III
TCGA-CC-A123-01A	219	0	24	Female	G1	Stage III
TCGA-DD-A119-01A	223	1	40	Male	G3	Stage IV
TCGA-MR-A520-01A	229	0	58	Male	G1	Stage I
TCGA-DD-A3A1-01A	233	1	65	Male	G2	Stage III
TCGA-FV-A3I1-01A	247	1	81	Female	G2	Stage II
TCGA-CC-A9FW-01A	248	0	68	Male	G2	Stage III
TCGA-CC-5259-01A	250	0	60	Female	G2	Stage III
TCGA-2Y-A9HB-01A	260	0	66	Male	G2	Stage I
TCGA-4R-AA8I-01A	262	1	66	Male	G2	Stage II
TCGA-CC-A7IK-01A	262	1	59	Male	G3	Stage III
TCGA-CC-A5UE-01A	272	1	48	Male	G2	Stage III
TCGA-CC-A7IL-01A	278	1	61	Male	G1	Stage III
TCGA-CC-A8HV-01A	279	1	51	Female	G2	Stage II
TCGA-DD-A73B-01A	283	1	72	Female	G2	Stage I
TCGA-MI-A75C-01A	291	0	64	Male	G3	Stage I
TCGA-ED-A66Y-01A	296	1	51	Female	G3	Stage III
TCGA-CC-A7IG-01A	299	1	47	Male	G2	Stage II
TCGA-CC-A3M9-01A	300	1	45	Male	G3	Stage III
TCGA-CC-A8HS-01A	300	1	18	Male	G1	Stage III
TCGA-EP-A3JL-01A	303	0	76	Male	G2	Stage I
TCGA-CC-A3MA-01A	303	1	61	Male	G2	Stage III
TCGA-CC-A5UD-01A	304	1	45	Male	G2	Stage III
TCGA-BC-A10R-01A	308	1	66	Female	G2	Stage III
TCGA-UB-AA0V-01A	314	0	69	Female	G1	Stage I
TCGA-CC-A3MB-01A	315	1	36	Male	G1	Stage III
TCGA-5C-A9VH-01A	322	0	70	Male	G2	Stage I
TCGA-UB-AA0U-01A	327	0	60	Male	G2	Stage II
TCGA-5C-A9VG-01A	328	0	58	Male	G2	Stage II
TCGA-MR-A8JO-01A	330	0	34	Male	G3	Stage I
TCGA-ZS-A9CG-01A	341	0	55	Male	G2	Stage II
TCGA-CC-A8HU-01A	344	1	39	Female	G3	Stage III
TCGA-WJ-A86L-01A	345	0	68	Female	G2	Stage I
TCGA-DD-AAC9-01A	347	0	51	Male	G2	Stage I
TCGA-CC-A5UC-01A	347	1	63	Male	G3	Stage III
TCGA-DD-A1EE-01A	349	1	73	Male	G3	Stage III
TCGA-G3-AAV5-01A	354	0	67	Male	G2	Stage II
TCGA-2Y-A9H6-01A	357	0	68	Female	G2	Stage I
TCGA-G3-AAV1-01A	359	1	51	Male	G3	Stage III
TCGA-K7-AAU7-01A	359	0	61	Male	G2	Stage II
TCGA-G3-A7M7-01A	361	0	65	Male	G1	Stage I
TCGA-G3-AAV7-01A	361	0	38	Male	G2	Stage II
TCGA-CC-A3MC-01A	363	0	54	Male	G2	Stage III
TCGA-EP-A3RK-01A	363	0	73	Male	G2	Stage III

TCGA-DD-AACF-01A	365	1	68	Male	G3	Stage I
TCGA-CC-A7IH-01A	365	0	58	Male	G1	Stage III
TCGA-LG-A9QD-01A	366	0	68	Male	G2	Stage III
TCGA-FV-A3R3-01A	366	1	38	Female	G2	Stage I
TCGA-G3-AAV2-01A	372	0	50	Male	G1	Stage I
TCGA-DD-A4NQ-01A	373	1	60	Male	G3	Stage II
TCGA-DD-AACD-01A	381	1	48	Male	G4	Stage I
TCGA-CC-A7IJ-01A	382	0	56	Male	G3	Stage II
TCGA-BC-A69I-01A	387	0	69	Male	G1	Stage I
TCGA-LG-A6GG-01A	387	0	79	Female	G2	Stage II
TCGA-ED-A7PY-01A	390	0	20	Female	G3	Stage II
TCGA-DD-A1EF-01A	394	1	57	Female	G3	Stage I
TCGA-WQ-AB4B-01A	395	0	62	Male	G2	Stage II
TCGA-ZP-A9D4-01A	395	0	64	Female	G1	Stage III
TCGA-3K-AAZ8-01A	396	0	65	Male	G1	Stage III
TCGA-CC-A7II-01A	399	0	54	Male	G3	Stage III
TCGA-ED-A7XP-01A	400	0	53	Female	G3	Stage II
TCGA-ED-A66X-01A	406	0	35	Male	G3	Stage III
TCGA-ED-A8O5-01A	406	0	59	Female	G3	Stage III
TCGA-ED-A82E-01A	408	0	60	Female	G2	Stage III
TCGA-BD-A3EP-01A	409	0	75	Female	G2	Stage I
TCGA-DD-AA3A-01A	410	1	81	Female	G4	Stage I
TCGA-G3-AAV3-01A	412	0	58	Female	G2	Stage II
TCGA-YA-A8S7-01A	412	1	68	Male	G3	Stage III
TCGA-DD-A1EL-01A	415	1	23	Male	G3	Stage II
TCGA-DD-AACP-01A	415	0	64	Male	G3	Stage I
TCGA-G3-A25S-01A	416	1	64	Male	G2	Stage I
TCGA-DD-A3A7-01A	419	1	67	Male	G3	Stage III
TCGA-ED-A627-01A	423	0	74	Male	G2	Stage I
TCGA-LG-A9QC-01A	425	0	48	Male	G2	Stage I
TCGA-DD-AADC-01A	425	1	53	Male	G3	Stage I
TCGA-ED-A7XO-01A	427	0	29	Male	G2	Stage III
TCGA-G3-A7M8-01A	430	0	31	Male	G1	Stage I
TCGA-DD-AACQ-01A	432	1	50	Male	G3	Stage II
TCGA-DD-AADQ-01A	436	0	59	Male	G3	Stage II
TCGA-ES-A2HT-01A	438	1	54	Male	G2	Stage I
TCGA-BC-A69H-01A	444	0	64	Male	G3	Stage II
TCGA-G3-A7M5-01A	447	0	76	Male	G2	Stage I
TCGA-5R-AA1D-01A	449	0	17	Female	G3	Stage III
TCGA-G3-A25Y-01A	452	1	52	Female	G3	Stage I
TCGA-DD-AADO-01A	453	0	55	Male	G3	Stage I
TCGA-DD-AADP-01A	458	0	45	Male	G3	Stage I
TCGA-RC-A7SH-01A	468	0	42	Male	G3	Stage II
TCGA-DD-AACG-01A	469	1	52	Male	G4	Stage II
TCGA-RC-A7SK-01A	472	0	59	Male	G3	Stage I
TCGA-DD-AADS-01A	474	0	63	Male	G2	Stage I
TCGA-G3-AAV0-01A	476	0	58	Male	G2	Stage I
TCGA-DD-AAEB-01A	478	0	60	Male	G2	Stage I
TCGA-G3-AAUZ-01A	480	0	48	Male	G2	Stage I
TCGA-UB-A7ME-01A	486	0	51	Male	G2	Stage I
TCGA-UB-A7MC-01A	500	0	59	Male	G3	Stage III
TCGA-MI-A75E-01A	507	0	61	Male	G2	Stage III
TCGA-K7-A6G5-01A	512	0	66	Male	G2	Stage I

TCGA-K7-A5RG-01A	519	0	66	Male	G1	Stage I
TCGA-G3-A5SM-01A	520	0	58	Male	G3	Stage II
TCGA-5R-AA1C-01A	520	0	57	Male	G2	Stage II
TCGA-DD-A3A3-01A	535	1	45	Male	G2	Stage I
TCGA-O8-A75V-01A	538	0	54	Male	G2	Stage I
TCGA-BC-A5W4-01A	547	1	69	Male	G3	Stage III
TCGA-DD-AAE1-01A	552	0	52	Male	G3	Stage I
TCGA-DD-AADU-01A	554	0	60	Male	G3	Stage II
TCGA-DD-AADY-01A	555	0	55	Female	G2	Stage I
TCGA-2Y-A9H5-01A	555	1	59	Female	G3	Stage I
TCGA-DD-AAE0-01A	555	0	45	Female	G4	Stage III
TCGA-WX-AA47-01A	556	1	33	Female	G2	Stage III
TCGA-DD-A1EK-01A	558	1	64	Female	G2	Stage IV
TCGA-BC-A8YO-01A	562	0	66	Female	G3	Stage III
TCGA-DD-AAD1-01A	564	0	51	Female	G4	Stage I
TCGA-DD-AAE3-01A	566	0	50	Male	G2	Stage I
TCGA-EP-A12J-01A	570	0	62	Male	G1	Stage I
TCGA-DD-AADV-01A	574	0	50	Male	G3	Stage I
TCGA-DD-AAEA-01A	575	0	65	Male	G3	Stage I
TCGA-RC-A7SF-01A	579	0	66	Male	G2	Stage I
TCGA-FV-A2QR-01A	581	1	75	Male	G1	Stage I
TCGA-G3-A3CK-01A	585	0	61	Male	G2	Stage I
TCGA-DD-AADW-01A	587	0	48	Male	G3	Stage I
TCGA-RC-A7SB-01A	588	0	53	Male	G2	Stage II
TCGA-G3-A3CJ-01A	594	0	52	Male	G2	Stage II
TCGA-EP-A2KB-01A	596	1	46	Female	G2	Stage I
TCGA-DD-A39Z-01A	601	1	43	Female	G2	Stage II
TCGA-UB-A7MB-01A	601	0	24	Male	G3	Stage II
TCGA-DD-A1EC-01A	602	0	20	Female	G3	Stage I
TCGA-DD-AAE4-01A	608	0	49	Female	G1	Stage I
TCGA-EP-A26S-01A	608	0	70	Male	G2	Stage I
TCGA-DD-A3A4-01A	612	1	37	Male	G3	Stage III
TCGA-WX-AA44-01A	615	0	64	Female	G3	Stage I
TCGA-G3-A5SL-01A	621	0	70	Male	G2	Stage II
TCGA-EP-A2KA-01A	627	1	52	Female	G3	Stage III
TCGA-MI-A75I-01A	630	0	61	Male	G1	Stage II
TCGA-K7-A5RF-01A	631	0	64	Male	G1	Stage I
TCGA-G3-A7M6-01A	632	0	60	Female	G3	Stage I
TCGA-2Y-A9H8-01A	633	1	85	Female	G2	Stage I
TCGA-DD-AADL-01A	636	0	58	Male	G4	Stage I
TCGA-DD-AAE2-01A	638	0	51	Male	G3	Stage I
TCGA-PD-A5DF-01A	639	1	58	Female	G2	Stage III
TCGA-RC-A7S9-01A	640	0	47	Female	G3	Stage I
TCGA-DD-A39V-01A	643	1	77	Male	G3	Stage II
TCGA-DD-AAE7-01A	644	0	72	Male	G2	Stage I
TCGA-CC-A7IF-01A	649	1	59	Male	G1	Stage III
TCGA-G3-A25Z-01A	655	0	58	Male	G2	Stage I
TCGA-DD-AAD2-01A	658	0	66	Male	G2	Stage I
TCGA-DD-A4NE-01A	660	1	75	Female	G3	Stage III
TCGA-DD-A11C-01A	662	0	69	Male	G3	Stage I
TCGA-G3-A6UC-01A	671	0	65	Male	G2	Stage III
TCGA-DD-AAD6-01A	672	0	66	Male	G3	Stage III
TCGA-G3-A3CG-01A	673	0	80	Male	G2	Stage I

TCGA-BC-A3KG-01A	680	0	68	Female	G3	Stage II
TCGA-ES-A2HS-01A	688	1	80	Male	G2	Stage I
TCGA-DD-A73D-01A	693	0	68	Female	G1	Stage II
TCGA-XR-A8TF-01A	693	1	74	Male	G1	Stage I
TCGA-2Y-A9H9-01A	697	0	70	Male	G2	Stage I
TCGA-MI-A75G-01A	698	0	63	Male	G2	Stage II
TCGA-G3-A5SJ-01A	698	0	59	Male	G2	Stage I
TCGA-DD-A73C-01A	701	0	65	Female	G1	Stage III
TCGA-ZP-A9CZ-01A	706	0	72	Male	G1	Stage III
TCGA-BC-A10Y-01A	711	1	76	Male	G3	Stage III
TCGA-DD-AAEG-01A	719	0	59	Female	G3	Stage I
TCGA-DD-AAE9-01A	722	0	69	Male	G3	Stage I
TCGA-2Y-A9GS-01A	724	1	58	Male	G2	Stage II
TCGA-DD-A73A-01A	728	0	71	Male	G2	Stage I
TCGA-FV-A2QQ-01A	729	0	80	Male	G2	Stage I
TCGA-G3-A5SK-01A	744	0	58	Male	G1	Stage I
TCGA-MI-A75H-01A	747	0	77	Male	G2	Stage II
TCGA-WX-AA46-01A	756	0	61	Male	G1	Stage II
TCGA-2Y-A9GY-01A	757	1	64	Female	G3	Stage II
TCGA-DD-AAED-01A	763	0	51	Male	G3	Stage I
TCGA-ZP-A9D2-01A	765	1	51	Male	G2	Stage III
TCGA-G3-A5SI-01A	768	1	44	Male	G2	Stage II
TCGA-BC-A10X-01A	770	1	52	Female	G2	Stage III
TCGA-G3-A3CH-01A	780	0	53	Male	G2	Stage III
TCGA-ZP-A9CY-01A	782	0	66	Female	G1	Stage III
TCGA-DD-AAEH-01A	784	0	73	Male	G2	Stage I
TCGA-NI-A8LF-01A	799	0	74	Male	G3	Stage I
TCGA-DD-A4NG-01A	802	1	77	Male	G2	Stage III
TCGA-DD-AAEE-01A	810	0	55	Male	G4	Stage I
TCGA-DD-A4NI-01A	816	0	67	Male	G2	Stage II
TCGA-ED-A4XI-01A	819	0	58	Male	G3	Stage II
TCGA-DD-A39W-01A	827	1	29	Female	G2	Stage III
TCGA-KR-A7K2-01A	829	0	64	Male	G1	Stage I
TCGA-BC-A10T-01A	837	1	76	Male	G1	Stage III
TCGA-BC-A10U-01A	837	1	69	Male	G2	Stage III
TCGA-2Y-A9GZ-01A	848	1	82	Female	G2	Stage II
TCGA-FV-A3I0-01A	848	0	76	Female	G2	Stage II
TCGA-UB-A7MA-01A	848	0	62	Female	G2	Stage II
TCGA-BC-4073-01B	849	0	73	Male	G3	Stage III
TCGA-ED-A5KG-01A	854	0	60	Female	G2	Stage II
TCGA-G3-A25V-01A	860	0	68	Male	G2	Stage I
TCGA-GJ-A3OU-01A	879	0	59	Male	G2	Stage I
TCGA-DD-AADN-01A	898	0	59	Male	G4	Stage I
TCGA-XR-A8TG-01A	898	0	58	Male	G2	Stage I
TCGA-DD-A4NN-01A	899	1	56	Female	G3	Stage I
TCGA-KR-A7K8-01A	906	0	57	Male	G1	Stage I
TCGA-ED-A459-01A	910	0	47	Male	G2	Stage II
TCGA-DD-A4NH-01A	917	0	65	Female	G3	Stage III
TCGA-XR-A8TE-01A	925	0	16	Male	G1	Stage III
TCGA-DD-A4NJ-01A	928	0	54	Female	G2	Stage II
TCGA-DD-A3A9-01A	931	1	64	Female	G2	Stage IV
TCGA-DD-A4NF-01A	942	0	72	Male	G2	Stage I
TCGA-KR-A7K7-01A	951	0	61	Female	G1	Stage II

TCGA-DD-A4NB-01A	989	0	25	Male	G2	Stage I
TCGA-DD-A1EJ-01A	1005	1	71	Female	G2	Stage III
TCGA-DD-A4NA-01A	1008	0	67	Female	G3	Stage III
TCGA-XR-A8TD-01A	1030	0	49	Female	G3	Stage III
TCGA-DD-AADK-01A	1049	0	68	Female	G3	Stage II
TCGA-DD-AADJ-01A	1066	0	70	Female	G3	Stage I
TCGA-DD-AAEK-01A	1067	0	51	Male	G3	Stage II
TCGA-DD-A73F-01A	1085	0	77	Female	G1	Stage I
TCGA-DD-AADI-01A	1085	0	43	Female	G3	Stage I
TCGA-ZP-A9CV-01A	1088	1	59	Male	G1	Stage III
TCGA-ZP-A9D0-01A	1091	0	67	Female	G1	Stage III
TCGA-RG-A7D4-01A	1098	0	69	Male	G2	Stage II
TCGA-BD-A3ER-01A	1115	0	62	Male	G2	Stage II
TCGA-BC-A10Q-01A	1135	1	72	Female	G3	Stage III
TCGA-DD-AADG-01A	1145	0	70	Male	G3	Stage III
TCGA-HP-A5N0-01A	1147	1	90	Female	G2	Stage I
TCGA-DD-A114-01A	1149	1	42	Male	G3	Stage II
TCGA-2Y-A9H7-01A	1168	0	81	Female	G2	Stage I
TCGA-DD-A4NK-01A	1210	1	80	Female	G2	Stage III
TCGA-DD-AAD8-01A	1219	0	73	Female	G2	Stage I
TCGA-2Y-A9H1-01A	1229	1	58	Male	G2	Stage I
TCGA-DD-AADD-01A	1231	0	51	Male	G4	Stage I
TCGA-DD-AADA-01A	1233	0	66	Female	G3	Stage I
TCGA-ZS-A9CE-01A	1241	0	79	Female	G1	Stage II
TCGA-DD-AADB-01A	1242	0	51	Male	G4	Stage I
TCGA-2Y-A9GW-01A	1271	1	64	Male	G2	Stage I
TCGA-DD-AAD3-01A	1295	0	43	Male	G2	Stage I
TCGA-DD-AACN-01A	1302	0	32	Male	G3	Stage I
TCGA-XR-A8TC-01A	1339	0	43	Female	G2	Stage I
TCGA-DD-AAD5-01A	1345	0	54	Male	G3	Stage I
TCGA-BC-A216-01A	1351	0	62	Female	G2	Stage III
TCGA-BD-A2L6-01A	1363	0	69	Male	G2	Stage III
TCGA-DD-A1EG-01A	1372	1	76	Male	G3	Stage I
TCGA-ZS-A9CD-01A	1386	1	73	Male	G2	Stage II
TCGA-BC-A217-01A	1397	1	75	Female	G3	Stage II
TCGA-BC-A10S-01A	1423	1	81	Male	G1	Stage III
TCGA-DD-AACW-01A	1424	0	43	Male	G3	Stage I
TCGA-DD-AACY-01A	1450	0	61	Male	G3	Stage I
TCGA-2Y-A9H4-01A	1452	0	68	Male	G2	Stage I
TCGA-BC-4072-01B	1490	1	74	Female	G3	Stage III
TCGA-DD-A1EH-01A	1495	0	23	Male	G3	Stage III
TCGA-2Y-A9H3-01A	1516	0	45	Male	G1	Stage II
TCGA-DD-AAEI-01A	1531	0	72	Male	G2	Stage I
TCGA-DD-AACV-01A	1531	0	53	Male	G3	Stage I
TCGA-G3-A25T-01A	1553	0	45	Female	G2	Stage III
TCGA-DD-A11D-01A	1560	1	57	Female	G2	Stage I
TCGA-DD-AACT-01A	1562	0	69	Female	G2	Stage I
TCGA-DD-AACU-01A	1567	0	59	Male	G3	Stage I
TCGA-DD-AACI-01A	1618	0	69	Male	G3	Stage II
TCGA-DD-A116-01A	1622	1	68	Male	G3	Stage III
TCGA-2Y-A9GT-01A	1624	1	51	Male	G2	Stage I
TCGA-DD-AAW3-01A	1633	0	69	Male	G2	Stage I
TCGA-G3-A25U-01A	1636	0	63	Female	G3	Stage I

TCGA-DD-AACC-01A	1685	1	61	Male	G2	Stage I
TCGA-DD-A39X-01A	1694	1	78	Female	G2	Stage I
TCGA-DD-A4NL-01A	1711	0	46	Male	G1	Stage I
TCGA-DD-AAVX-01A	1718	0	38	Male	G2	Stage II
TCGA-2Y-A9H2-01A	1731	0	64	Female	G3	Stage I
TCGA-G3-A25X-01A	1779	0	73	Male	G3	Stage II
TCGA-NI-A4U2-01A	1791	1	71	Male	G1	Stage III
TCGA-DD-AACS-01A	1804	0	39	Male	G3	Stage I
TCGA-DD-AAVS-01A	1823	0	56	Male	G2	Stage I
TCGA-FV-A23B-01A	1852	1	70	Female	G2	Stage II
TCGA-DD-AAW2-01A	1855	0	69	Male	G2	Stage I
TCGA-DD-AACO-01A	1876	0	40	Male	G3	Stage I
TCGA-DD-AAVZ-01A	1900	0	38	Male	G2	Stage I
TCGA-2Y-A9GU-01A	1939	0	55	Female	G2	Stage I
TCGA-DD-AAVY-01A	1970	0	56	Male	G2	Stage III
TCGA-DD-AAW1-01A	1989	0	55	Male	G2	Stage III
TCGA-DD-AAW0-01A	2015	0	54	Male	G2	Stage I
TCGA-DD-A1EB-01A	2017	0	72	Female	G2	Stage I
TCGA-DD-AADR-01A	2028	0	58	Male	G3	Stage I
TCGA-DD-AACJ-01A	2102	0	75	Male	G2	Stage II
TCGA-BC-A110-01A	2116	1	51	Female	G1	Stage I
TCGA-DD-A3A2-01A	2131	1	76	Female	G1	Stage I
TCGA-DD-AACE-01A	2184	0	62	Male	G3	Stage I
TCGA-DD-AAVU-01A	2202	0	46	Male	G2	Stage II
TCGA-DD-A4NO-01A	2245	0	65	Male	G1	Stage I
TCGA-DD-A1ED-01A	2301	0	68	Male	G1	Stage I
TCGA-DD-AACA-02B	2301	0	65	Male	G3	Stage I
TCGA-DD-AACA-01A	2301	0	65	Male	G3	Stage I
TCGA-DD-AACA-02A	2301	0	65	Male	G3	Stage I
TCGA-DD-AAVW-01A	2317	0	35	Male	G2	Stage I
TCGA-DD-AACB-01A	2324	0	74	Female	G3	Stage I
TCGA-DD-A4NV-01A	2398	0	61	Male	G1	Stage III
TCGA-ZS-A9CF-01A	2412	0	64	Male	G2	Stage II
TCGA-ZS-A9CF-02A	2412	0	64	Male	G2	Stage II
TCGA-DD-A1EA-01A	2415	0	68	Male	G2	Stage II
TCGA-DD-A113-01A	2425	0	55	Female	G3	Stage II
TCGA-2Y-A9GX-01A	2442	0	68	Male	G2	Stage I
TCGA-DD-AAVV-01A	2455	0	56	Male	G3	Stage II
TCGA-DD-A4NS-01A	2456	1	61	Female	G2	Stage I
TCGA-FV-A4ZP-01A	2486	1	78	Male	G2	Stage III
TCGA-DD-AAVR-01A	2513	0	44	Male	G2	Stage I
TCGA-2Y-A9GV-01A	2532	1	54	Female	G1	Stage I
TCGA-DD-A115-01A	2542	1	53	Male	G2	Stage III
TCGA-DD-AAVQ-01A	2728	0	38	Male	G2	Stage I
TCGA-DD-A4ND-01A	2746	0	56	Female	G3	Stage I
TCGA-DD-AAVP-01A	2752	0	48	Male	G1	Stage I
TCGA-DD-A3A5-01A	3125	1	66	Female	G2	Stage III
TCGA-DD-A3A6-01A	3258	1	72	Female	G2	Stage II
TCGA-DD-A4NP-01A	3308	0	32	Male	G3	Stage I
TCGA-DD-A118-01A	3437	0	77	Female	G2	Stage II
TCGA-DD-A73G-01A	3478	0	73	Female	G3	Stage I
TCGA-2Y-A9H0-01A	3675	0	49	Male	G1	Stage III
