# Supplementary material for: A Cell Cycle Progression-Derived Gene Signature to Predict Prognosis and Therapeutic Response in Hepatocellular Carcinoma
Source: Dis Markers. 2021 Oct 21;2021:1986159. doi: 10.1155/2021/1986159 (PMC8553501; doi:10.1155/2021/1986159)
Supplement: Supplementary Materials — Supplementary table 1. Clinical information of HCC patients in TCGA-LIHC cohort. Supplementary table 2. The gene sets of hallmarks of cancer. Supplementary table 3. 549 CCP-relevant genes in TCGA-LIHC cohort. Supplementary table 4. Prognostic CCP-relevant genes in TCGA-LIHC cohort. [file 1986159.f1.zip › 1986159.f1/Supplementary table 2 (1).pdf]

Supplementary table 2. The gene sets of hallmarks of cancer.

glycolysis	CCP	angiogenesis	apoptosis	DNA repair	EMT
ABCB6	ABL1	APOH	ADD1	AAAS	ABI3BP
ADORA2B	ACVR1	APP	AIFM3	ADA	ACTA2
AGL	ACVR1B	CCND2	ANKH	ADCY6	ADAM12
AGRN	AKAP8	COL3A1	ANXA1	ADRM1	ANPEP
AK3	ANAPC10	COL5A2	APP	AGO4	APLP1
AK4	ANAPC11	CXCL6	ATF3	AK1	AREG
AKR1A1	ANAPC4	FGFR1	AVPR1A	AK3	BASP1
ALDH7A1	ANAPC5	FSTL1	BAX	ALYREF	BDNF
ALDH9A1	ANLN	ITGAV	BCAP31	APRT	BGN
ALDOA	APBB1	JAG1	BCL10	ARL6IP1	BMP1
ALDOB	APBB2	JAG2	BCL2L1	BCAM	CADM1
ALG1	ATM	KCNJ8	BCL2L10	BCAP31	CALD1
ANG	AURKA	LPL	BCL2L11	BOLA2	CALU
ANGPTL4	BCAT1	LRPAP1	BCL2L2	BRF2	CAP2
ANKZF1	BIRC5	LUM	BGN	CANT1	CAPG
ARPP19	BOLL	MSX1	BID	CCNO	CCN1
ARTN	BRSK1	NRP1	BIK	CDA	CCN2
AURKA	BUB1	OLR1	BIRC3	CETN2	CD44
B3GALT6	BUB1B	PDGFA	BMF	CLP1	CD59
B3GAT1	CCNA1	PF4	BMP2	CMPK2	CDH11
B3GAT3	CCNA2	PGLYRP1	BNIP3L	COX17	CDH2
B3GNT3	CD28	POSTN	BRCA1	CSTF3	CDH6
B4GALT1	CDC16	PRG2	BTG2	DAD1	COL11A1
B4GALT2	CDC23	PTK2	BTG3	DCTN4	COL12A1
B4GALT4	CDC25B	S100A4	CASP1	DDB1	COL16A1
B4GALT7	CDC25C	SERPINA5	CASP2	DDB2	COL1A1
BIK	CDC27	SLCO2A1	CASP3	DGCR8	COL1A2
BPNT1	CDC6	SPP1	CASP4	DGUOK	COL3A1
CACNA1H	CDC7	STC1	CASP6	DUT	COL4A1
CAPN5	CDCA5	THBD	CASP7	EDF1	COL4A2
CASP6	CDK10	TIMP1	CASP8	EIF1B	COL5A1
CD44	CDK13	TNFRSF21	CASP9	ELL	COL5A2
CDK1	CDK2	VAV2	CAV1	ELOA	COL5A3
CENPA	CDK2AP1	VCAN	CCNA1	ERCC1	COL6A2
CHPF	CDK4	VEGFA	CCND1	ERCC2	COL6A3
CHPF2	CDK6	VTN	CCND2	ERCC3	COL7A1
CHST1	CDKN1A		CD14	ERCC4	COL8A2
CHST12	CDKN1B		CD2	ERCC5	COLGALT1
CHST2	CDKN1C		CD38	ERCC8	COMP
CHST4	CDKN2A		CD44	FEN1	COPA
CHST6	CDKN2B		CD69	GMPR2	CRLF1
CITED2	CDKN2C		CDC25B	GPX4	CTHRC1
CLDN3	CDKN2D		CDK2	GSDME	CXCL1
CLDN9	CDKN3		CDKN1A	GTF2A2	CXCL12
CLN6	CENPE		CDKN1B	GTF2B	CXCL6
COG2	CENPF		CFLAR	GTF2F1	CXCL8
COL5A1	CETN1		CLU	GTF2H1	DAB2
COPB2	CHEK1		CREBBP	GTF2H3	DCN
CTH	CHFR		CTH	GTF2H5	DKK1
CXCR4	CHMP1A		CTNNB1	GTF3C5	DPYSL3
CYB5A	CIT		CYLD	GUK1	DST

DCN	CLIP1	DAP	HCLS1	ECM1
DDIT4	CUL1	DAP3	HPRT1	ECM2
DEPDC1	CUL2	DCN	IMPDH2	EDIL3
DLD	CUL3	DDIT3	ITPA	EFEMP2
DPYSL4	CUL4A	DFFA	LIG1	ELN
DSC2	CUL5	DIABLO	MPC2	EMP3
ECD	DBF4	DNAJA1	MPG	ENO2
EFNA3	DCTN2	DNAJC3	MRPL40	FAP
EGFR	DCTN3	DNM1L	NCBP2	FAS
EGLN3	DDX11	DPYD	NELFB	FBLN1
ELF3	DLGAP5	EBP	NELFCD	FBLN2
ENO1	DMC1	EGR3	NELFE	FBLN5
ENO2	DUSP13	EMP1	NFX1	FBN1
ERO1A	E2F1	ENO2	NME1	FBN2
EXT1	EGF	ERBB2	NME3	FERMT2
EXT2	EPGN	ERBB3	NME4	FGF2
FAM162A	EREG	EREG	NPR2	FLNA
FBP2	ESPL1	ETF1	NT5C	FMOD
FKBP4	FOXN3	F2	NT5C3A	FN1
FUT8	FOXO4	F2R	NUDT21	FOXC2
G6PD	GFI1	FAS	NUDT9	FSTL1
GAL3ST1	GFI1B	FASLG	PCNA	FSTL3
GALE	GML	FDXR	PDE4B	FUCA1
GALK1	GSPT1	FEZ1	PDE6G	FZD8
GALK2	HSPA2	GADD45A	PNP	GADD45A
GAPDHS	INHBA	GADD45B	POLA1	GADD45B
GCLC	KATNA1	GCH1	POLA2	GAS1
GFPT1	KHDRBS1	GNA15	POLB	GEM
GFUS	KIF11	GPX1	POLD1	GJA1
GLCE	KIF15	GPX3	POLD3	GLIPR1
GLRX	KIF22	GPX4	POLD4	GPC1
GMPPA	KIF25	GSN	POLE4	GPX7
GMPPB	KIF2C	GSR	POLH	GREM1
GNE	KNTC1	GSTM1	POLL	HTRA1
GNPDA1	KPNA2	GUCY2D	POLR1C	ID2
GOT1	KRT7	H1-0	POLR1D	IGFBP2
GOT2	LATS1	HGF	POLR1H	IGFBP3
GPC1	LATS2	HMGB2	POLR2A	IGFBP4
GPC3	LIG3	HMOX1	POLR2C	IL15
GPC4	MAD2L1	HSPB1	POLR2D	IL32
GPR87	MAD2L2	IER3	POLR2E	IL6
GUSB	MAP3K11	IFITM3	POLR2F	INHBA
GYS1	MPHOSPH6	IFNB1	POLR2G	ITGA2
GYS2	MPHOSPH9	IFNGR1	POLR2H	ITGA5
HAX1	MRE11	IGF2R	POLR2I	ITGAV
HDLBP	MSH4	IGFBP6	POLR2J	ITGB1
HK2	MSH5	IL18	POLR2K	ITGB3
HMMR	MYO16	IL1A	POLR3C	ITGB5
HOMER1	NBN	IL1B	POLR3GL	JUN
HS2ST1	NCAPH	IL6	POM121	LAMA1
HS6ST2	NDC80	IRF1	PRIM1	LAMA2
HSPA5	NEK2	ISG20	RAD51	LAMA3
IDH1	NEK6	JUN	RAD52	LAMC1

IDUA	NOLC1	KRT18	RAE1	LAMC2
IER3	NPM2	LEF1	RALA	LGALS1
IGFBP3	NUMA1	LGALS3	RBX1	LOX
IL13RA1	NUSAP1	LMNA	REV3L	LOXL1
IRS2	P3H4	LUM	RFC2	LOXL2
ISG20	PAM	MADD	RFC3	LRP1
KDELR3	PBRM1	MCL1	RFC4	LRRC15
KIF20A	PCBP4	MGMT	RFC5	LUM
KIF2A	PDS5B	MMP2	RNMT	MAGEE1
LCT	PIM2	NEDD9	RPA2	MATN2
LDHA	PIN1	NEFH	RPA3	MATN3
LDHC	PKMYT1	PAK1	RRM2B	MCM7
LHPP	PLK1	PDCD4	SAC3D1	MEST
LHX9	PML	PDGFRB	SDCBP	MFAP5
MDH1	POLA1	PEA15	SEC61A1	MGP
MDH2	POLD1	PLAT	SF3A3	MMP1
ME1	POLE	PLCB2	SMAD5	MMP14
ME2	PPP5C	PLPPR4	SNAPC4	MMP2
MED24	PPP6C	PMAIP1	SNAPC5	MMP3
MERTK	PRMT5	PPP2R5B	SRSF6	MSX1
MET	PRUNE2	PPP3R1	SSRP1	MXRA5
MIF	PTPRC	PPT1	STX3	MYL9
MIOX	RAD1	PRF1	SUPT4H1	MYLK
MPI	RAD17	PSEN1	SUPT5H	NID2
MXI1	RAD21	PSEN2	SURF1	NNMT
NANP	RAD50	PTK2	TAF10	NOTCH2
NASP	RAD51	RARA	TAF12	NT5E
NDST3	RAD51B	RELA	TAF13	NTM
NDUFV3	RAD51D	RETSAT	TAF1C	OXTR
NOL3	RAD52	RHOB	TAF6	P3H1
NSDHL	RAD54B	RHOT2	TAF9	PCOLCE
NT5E	RAD54L	RNASEL	TARBP2	PCOLCE2
P4HA1	RAN	ROCK1	TK2	PDGFRB
P4HA2	RB1	SAT1	TMED2	PDLIM4
PAM	RCC1	SATB1	TP53	PFN2
PAXIP1	REC8	SC5D	TSG101	PLAUR
PC	RINT1	SLC20A1	TYMS	PLOD1
PDK3	SKP2	SMAD7	UMPS	PLOD2
PFKFB1	SMC1A	SOD1	UPF3B	PLOD3
PFKP	SMC3	SOD2	USP11	PMEPA1
PGAM1	SMC4	SPTAN1	VPS28	PMP22
PGAM2	SPDYA	SQSTM1	VPS37B	POSTN
PGK1	SPO11	TAP1	VPS37D	PPIB
PGLS	STAG3	TGFB2	XPC	PRRX1
PGM2	SUGT1	TGFBR3	ZNF707	PRSS2
PHKA2	SYCP1	TIMP1	ZWINT	PTHLH
PKM	TAF1	TIMP2		PTX3
PKP2	TAF1L	TIMP3		PVR
PLOD1	TARDBP	TNF		QSOX1
PLOD2	TBRG4	TNFRSF12A		RGS4
PMM2	TGFA	TNFSF10		RHOB
POLR3K	TGFB1	TOP2A		SAT1
PPFIA4	TIMELESS	TSPO		SCG2

PPIA	TIPIN	TXNIP	SDC1
PPP2CB	TOP3A	VDAC2	SDC4
PRPS1	TPD52L1	WEE1	SERPINE1
PSMC4	TPX2	XIAP	SERPINE2
PYGB	TRIAP1		SERPINH1
PYGL	TTK		SFRP1
QSOX1	TTN		SFRP4
RARS1	UBE2C		SGCB
RBCK1	USH1C		SGCD
RPE	XRCC2		SGCG
RRAGD	ZNRD2		SLC6A8
SAP30	ZW10		SLIT2
SDC1	ZWINT		SLIT3
SDC2			SNAI2
SDC3			SNTB1
SDHC			SPARC
SLC16A3			SPOCK1
SLC25A10			SPP1
SLC25A13			TAGLN
SLC35A3			TFPI2
SLC37A4			TGFB1
SOD1			TGFBI
SOX9			TGFBR3
SPAG4			TGM2
SRD5A3			THBS1
STC1			THBS2
STC2			THY1
STMN1			TIMP1
TALDO1			TIMP3
TFF3			TNC
TGFA			TNFAIP3
TGFBI			TNFRSF11B
TKTL1			TNFRSF12A
TPBG			TPM1
TPI1			TPM2
TPST1			TPM4
TXN			VCAM1
UGP2			VCAN
VCAN			VEGFA
VEGFA			VEGFC
VLDLR			VIM
XYLT2			WIPF1
ZNF292			WNT5A

hypoxia	inflammation	stemness
ACKR3	ABCA1	DNMT3B
ADM	ABI1	MCM6
ADORA2B	ACVR1B	CDC25A
AK4	ACVR2A	PFAS
AKAP12	ADGRE1	MCM4
ALDOA	ADM	XRCC5
ALDOB	ADORA2B	HAUS6
ALDOC	ADRM1	TET1
AMPD3	AHR	IGF2BP1
ANGPTL4	APLNR	PLAA
ANKZF1	AQP9	DEPDC1B
ANXA2	ATP2A2	TEX10
ATF3	ATP2B1	CCDC99
ATP7A	ATP2C1	MSH2
B3GALT6	AXL	BUB1B
B4GALNT2	BDKRB1	MSH6
BCAN	BEST1	DLGAP5
BCL2	BST2	SKIV2L2
BGN	BTG2	CENPE
BHLHE40	C3AR1	CHEK2
BNIP3L	C5AR1	SOHLH2
BRS3	CALCRL	CCNB1
BTG1	CCL17	RRAS2
CA12	CCL2	PRIM1
CASP6	CCL20	PAICS
CAV1	CCL22	CCNA2
CAVIN1	CCL24	CPSF3
CAVIN3	CCL5	NUSAP1
CCN1	CCL7	LIN28B
CCN2	CCR7	IPO5
CCN5	CCRL2	KIF11
CCNG2	CD14	BMPR1A
CDKN1A	CD40	NDC80
CDKN1B	CD48	BCAT1
CDKN1C	CD55	CCNG1
CHST2	CD69	ZNF788
CHST3	CD70	ASCC3
CITED2	CD82	FANCB
COL5A1	CDKN1A	MCM10
CP	CHST2	HMGA2
CSRP2	CLEC5A	SKP2
CXCR4	CMKLR1	TRIM24
DCN	CSF1	ORC1
DDIT3	CSF3	HDAC2
DDIT4	CSF3R	HESX1
DPYSL4	CX3CL1	C1orf135
DTNA	CXCL10	INHBE
DUSP1	CXCL11	MIS18A
EDN2	CXCL6	DCUN1D5
EFNA1	CXCL8	POLE2
EFNA3	CXCL9	MRPL3

EGFR	CXCR6	CENPH
ENO1	CYBB	MYCN
ENO2	DCBLD2	HAUS1
ENO3	EBI3	GDF3
ERO1A	EDN1	TBCE
ERRFI1	EIF2AK2	RIOK2
ETS1	EMP3	BCKDHB
EXT1	EREG	RAD1
F3	F3	NREP
FAM162A	FFAR2	ADH5
FBP1	FPR1	PLRG1
FOS	FZD5	ROR1
FOSL2	GABBR1	RAB3B
FOXO3	GCH1	LOC285431
GAA	GNA15	DBC1
GALK1	GNAI3	KIF23
GAPDH	GP1BA	DIAPH3
GAPDHS	GPC3	GNL2
GBE1	GPR132	FGF2
GCK	GPR183	TARDBP
GCNT2	HAS2	NMNAT2
GLRX	HBEGF	ZNF167
GPC1	HIF1A	KIF20A
GPC3	HPN	CENPI
GPC4	HRH1	DDX1
GPI	ICAM1	XXYLT1
GRHPR	ICAM4	GPR176
GYS1	ICOSLG	FBXO22
HAS1	IFITM1	BBS9
HDLBP	IFNAR1	C14orf166
HEXA	IFNGR2	BOD1
HK1	IL10	CDC123
HK2	IL10RA	SNRPD3
HMOX1	IL12B	FAM118B
HOXB9	IL15	DPH3
HS3ST1	IL15RA	EIF2B3
HSPA5	IL18	KDELC1
IDS	IL18R1	RPF2
IER3	IL18RAP	APLP1
IGFBP1	IL1A	DACT1
IGFBP3	IL1B	PDHB
IL6	IL1R1	C14orf119
ILVBL	IL2RB	DTD1
INHA	IL4R	SAMM50
IRS2	IL6	CCL26
ISG20	IL7R	C4orf52
JMJD6	INHBA	CCDC90B
JUN	IRAK2	MED20
KDELR3	IRF1	UTP6
KDM3A	IRF7	RARS2
KIF5A	ITGA5	KIAA0020
KLF6	ITGB3	ARMCX2
KLF7	ITGB8	RARS

KLHL24	KCNA3	MTHFD2
LALBA	KCNJ2	DHX15
LARGE1	KCNMB2	HTR7
LDHA	KIF1B	HIST1H4C
LDHC	KLF6	MTHFD1L
LOX	LAMP3	ARMC9
LXN	LCK	XPOT
MAFF	LCP2	IARS
MAP3K1	LDLR	HDX
MIF	LIF	ACTRT3
MT1E	LPAR1	ERCC2
MT2A	LTA	TBC1D16
MXI1	LY6E	GARS
MYH9	LYN	KIF7
NAGK	MARCO	UBE2K
NCAN	MEFV	SLC25A3
NDRG1	MEP1A	ICMT
NDST1	MET	UGGT2
NDST2	MMP14	ATP11C
NEDD4L	MSR1	SLC24A1
NFIL3	MXD1	EIF2AK4
NOCT	MYC	GPX8
NR3C1	NAMPT	ALX1
P4HA1	NDP	OSTC
P4HA2	NFKB1	TRPC4
PAM	NFKBIA	HAS2
PCK1	NLRP3	FZD2
PDGFB	NMI	TRNT1
PDK1	NMUR1	MMADHC
PDK3	NOD2	SNX8
PFKFB3	NPFFR2	CDH6
PFKL	OLR1	HAT1
PFKP	OPRK1	SEC11A
PGAM2	OSM	DIMT1
PGF	OSMR	TM2D2
PGK1	P2RX4	FST
PGM1	P2RX7	GBE1
PGM2	P2RY2	NA
PHKG1	PCDH7	MLL3
PIM1	PDE4B	MXI1
PKLR	PDPN	FKSG49
PKP1	PIK3R5	FAM185BP
PLAC8	PLAUR	ARRB2
PLAUR	PROK2	SMARCC2
PLIN2	PSEN1	WASH3P
PNRC1	PTAFR	PILRB
PPARGC1A	PTGER2	CTSH
PPFIA4	PTGER4	SAT1
PPP1R15A	PTGIR	JUNB
PPP1R3C	PTPRE	CD53
PRDX5	PVR	PECAM1
PRKCA	RAF1	IL10RA
PYGM	RASGRP1	RCSD1

RBPJ	RELA	ARHGDIB
RORA	RGS1	GIMAP5
RRAGD	RGS16	GIMAP6
S100A4	RHOG	HLA-DMB
SAP30	RIPK2	PTPRC
SCARB1	RNF144B	C10orf128
SDC2	ROS1	CMBL
SDC3	RTP4	HLA-DRB5
SDC4	SCARF1	HLA-DPA1
SELENBP1	SCN1B	ABCG1
SERPINE1	SELE	GIMAP7
SIAH2	SELENOS	HLA-DQA1
SLC25A1	SELL	TSHZ2
SLC2A1	SEMA4D	RGCC
SLC2A3	SERPINE1	CCR1
SLC2A5	SGMS2	NPR3
SLC37A4	SLAMF1	RSAD2
SLC6A6	SLC11A2	GIMAP1
SRPX	SLC1A2	TNFSF10
STBD1	SLC28A2	AFTPH
STC1	SLC31A1	NA
STC2	SLC31A2	MALAT1
SULT2B1	SLC4A4	UBXN2A
TES	SLC7A1	PDE4C
TGFB3	SLC7A2	GIMAP8
TGFBI	SPHK1	FYB
TGM2	SRI	MS4A7
TIPARP	STAB1	C5orf56
TKTL1	TACR1	LOC400931
TMEM45A	TACR3	MLLT6
TNFAIP3	TAPBP	CTSS
TPBG	TIMP1	ZBTB20
TPD52	TLR1	
TPI1	TLR2	
TPST2	TLR3	
UGP2	TNFAIP6	
VEGFA	TNFRSF1B	
VHL	TNFRSF9	
VLDLR	TNFSF10	
WSB1	TNFSF15	
XPNPEP1	TNFSF9	
ZFP36	TPBG	
ZNF292	VIP	
