# Supplementary material for: A Cell Cycle Progression-Derived Gene Signature to Predict Prognosis and Therapeutic Response in Hepatocellular Carcinoma
Source: Dis Markers. 2021 Oct 21;2021:1986159. doi: 10.1155/2021/1986159 (PMC8553501; doi:10.1155/2021/1986159)
Supplement: Supplementary Materials — Supplementary table 1. Clinical information of HCC patients in TCGA-LIHC cohort. Supplementary table 2. The gene sets of hallmarks of cancer. Supplementary table 3. 549 CCP-relevant genes in TCGA-LIHC cohort. Supplementary table 4. Prognostic CCP-relevant genes in TCGA-LIHC cohort. [file 1986159.f1.zip › 1986159.f1/Supplementary table 3 (1).pdf]

Supplementary table 3. 549 CCP-relevant genes in TCGA-LIHC cohort.

ID	logFC	AveExpr	t	P.Value
MYBL2	-2.686224097	3.314349388	-22.51861555	4.04E-71
AFP	-2.518734315	3.965579311	-7.646101808	1.84E-13
TOP2A	-2.516826103	3.481298415	-25.85391432	1.24E-84
CDC20	-2.41512101	3.792740772	-21.35546661	2.55E-66
UBE2C	-2.364886718	4.414770272	-19.62452789	3.99E-59
BIRC5	-2.232578652	3.673032281	-20.99391794	8.05E-65
FOXM1	-2.23240965	2.996869528	-24.09902091	1.41E-77
KIFC1	-2.208389087	3.194365632	-24.42405543	6.82E-79
CDK1	-2.203677399	3.592864492	-24.14169007	9.49E-78
TPX2	-2.201911138	3.705496134	-25.20186758	5.02E-82
CCNB1	-2.106474154	3.972018628	-23.36939847	1.32E-74
NEK2	-2.091284921	2.732869906	-24.87206704	1.06E-80
RRM2	-2.065357635	3.681003745	-23.20035959	6.47E-74
PTTG1	-2.05685817	4.456004786	-18.03060914	1.75E-52
NUF2	-2.051958958	2.523576938	-23.61583318	1.30E-75
PEG10	-2.050843107	2.991025734	-7.064408198	8.17E-12
CCNB2	-2.037240963	2.812667005	-23.9157643	7.84E-77
KIF2C	-2.036548481	2.529992127	-22.98849525	4.76E-73
CDKN3	-2.018016566	3.679742784	-20.27497618	7.82E-62
CENPM	-2.017306232	3.368038998	-19.14392121	4.02E-57
MCM2	-2.016975529	3.88220618	-21.45541463	9.84E-67
NUSAP1	-2.010996802	3.962394174	-23.78496757	2.67E-76
TROAP	-2.009897571	2.511835204	-22.63346281	1.36E-71
AURKB	-2.006385903	3.308269287	-19.46410732	1.86E-58
KIF20A	-2.005701673	2.534955899	-23.63106488	1.13E-75
PLK1	-1.997996739	2.351159517	-24.13523556	1.01E-77
CCNA2	-1.983595182	2.824986052	-21.02792019	5.82E-65
KIF4A	-1.983471506	2.278998649	-25.61389819	1.13E-83
PRC1	-1.974918814	3.011664805	-24.17418479	7.01E-78
ANLN	-1.95716973	2.229237421	-22.77929026	3.43E-72
ZWINT	-1.954197437	3.85487314	-23.24197109	4.37E-74
ASF1B	-1.951394973	2.934433701	-21.8823941	1.69E-68
GINS1	-1.948331376	2.386834436	-26.25501496	3.17E-86
DEPDC1B	-1.940390525	1.991771756	-21.17905303	1.37E-65
LMNB1	-1.938216187	3.548588139	-23.5564581	2.27E-75
MELK	-1.926748514	2.408163123	-24.17596091	6.89E-78
CDCA8	-1.899142343	2.65646171	-23.52965882	2.93E-75
E2F1	-1.890753769	3.612948895	-16.89118366	9.44E-48
HJURP	-1.889348408	2.281740715	-24.11155009	1.26E-77
CDCA5	-1.879514994	3.080772894	-21.96798835	7.49E-69
BUB1	-1.874478126	2.190236496	-25.64290378	8.63E-84
CENPF	-1.871761556	2.19396315	-23.02742051	3.30E-73
AC099850.3	-1.866273353	1.867989009	-21.62940481	1.88E-67
CDT1	-1.864452259	2.73448044	-21.12603982	2.28E-65
MKI67	-1.857121215	2.259606202	-22.78945176	3.12E-72
NCAPG	-1.8438558	2.092232502	-25.77479768	2.57E-84
PRR11	-1.838958065	2.321911261	-22.83561161	2.02E-72
CENPA	-1.838289234	2.075595272	-22.13644899	1.51E-69
TCF19	-1.816191862	3.238584987	-19.83093519	5.52E-60
TYMS	-1.8136739	4.255410612	-18.25311631	2.08E-53
BUB1B	-1.810983803	1.864470574	-24.93361145	6.01E-81

ECT2	-1.805359988	2.505046028	-21.16628794	1.55E-65
KIF23	-1.801430378	1.89681609	-23.12416687	1.33E-73
NDC80	-1.80034504	2.721363995	-22.22606819	6.46E-70
KIF18B	-1.798690172	1.730787679	-24.14855158	8.90E-78
DLGAP5	-1.789071122	2.044805651	-22.87268316	1.42E-72
CDC6	-1.785093723	2.381291439	-21.29641572	4.48E-66
PCLAF	-1.779713679	2.889100248	-21.53203718	4.74E-67
PBK	-1.768658421	2.368915345	-20.40216682	2.31E-62
GTSE1	-1.760645513	1.843768298	-22.74753368	4.63E-72
CDC45	-1.753705043	2.476051994	-21.82366734	2.95E-68
TRIP13	-1.729732527	2.037036241	-21.24645534	7.22E-66
TACC3	-1.729400908	4.080633495	-19.92858019	2.16E-60
DTL	-1.728863159	2.269804175	-21.6199094	2.05E-67
CA9	-1.717029041	1.553522513	-8.011693449	1.52E-14
CD24	-1.713675574	5.32102803	-6.789093383	4.56E-11
HMMR	-1.704942712	2.458328593	-20.75438847	7.94E-64
UBE2T	-1.699236182	4.030334177	-19.17625287	2.95E-57
NCAPH	-1.691943292	2.060442217	-21.89217002	1.54E-68
STMN1	-1.69182879	5.409993309	-19.42168261	2.80E-58
SKA1	-1.689447546	1.894114365	-21.18085559	1.35E-65
SPP1	-1.686912414	6.860794583	-4.83607258	1.95E-06
TTK	-1.676378886	1.666096708	-23.97944089	4.32E-77
C12orf75	-1.670933122	3.297794989	-9.642094157	9.33E-20
KRT19	-1.669715751	2.428019219	-6.533220053	2.16E-10
PKM	-1.669129104	6.023934423	-11.18575685	3.45E-25
GAL3ST1	-1.65948424	2.853787558	-8.245800598	2.97E-15
UHRF1	-1.657587612	1.672942833	-20.55427028	5.39E-63
CDCA3	-1.653073894	2.334383972	-20.88402214	2.30E-64
CDC25C	-1.652368982	2.124670956	-20.95552632	1.16E-64
ASPM	-1.651585017	1.961493807	-21.34006869	2.96E-66
TK1	-1.650740043	5.022943245	-16.1004867	1.73E-44
SPC25	-1.649277658	2.270589376	-22.90248365	1.07E-72
PKMYT1	-1.646786339	2.376374444	-20.41474945	2.05E-62
UPK3A	-1.646180437	1.852123194	-7.276387977	2.10E-12
CENPW	-1.645822647	3.700890824	-17.43580488	5.21E-50
GPC3	-1.63953795	7.310053749	-5.51975907	6.43E-08
RACGAP1	-1.639495506	3.144255789	-21.28423375	5.03E-66
CEP55	-1.638807369	1.719688836	-20.22216298	1.30E-61
RMI2	-1.634490882	2.702329016	-17.91224961	5.45E-52
FANCI	-1.633490985	2.463004938	-23.22532235	5.11E-74
MCM4	-1.608961111	4.300772109	-18.95274949	2.52E-56
G6PD	-1.597819746	4.320217322	-11.55856297	1.47E-26
RAD51AP1	-1.595192745	1.981563661	-22.21077634	7.47E-70
SPHK1	-1.590913503	2.72957643	-8.542201163	3.58E-16
H19	-1.590839412	5.270164666	-5.022918833	7.97E-07
SPAG5	-1.578251119	3.646009797	-18.84552117	7.05E-56
DUSP9	-1.576517478	2.879044398	-8.241049232	3.07E-15
CENPK	-1.57449313	1.838563492	-22.70180837	7.14E-72
SKA3	-1.56663535	1.755500593	-23.65407565	9.10E-76
MCM6	-1.565505493	3.911841688	-19.71158889	1.73E-59
TUBA1B	-1.564190435	5.588661234	-14.74919502	5.59E-39
HMGB2	-1.559662364	5.349141896	-19.42653672	2.67E-58
ORC6	-1.558816553	1.910564916	-20.80408007	4.94E-64

KIF11	-1.553906319	1.956978513	-22.96070246	6.19E-73
DSG2	-1.543919432	3.206209669	-10.72283642	1.62E-23
SPC24	-1.531889209	3.091760466	-15.74296326	5.06E-43
EPCAM	-1.531637047	2.799380499	-5.054760548	6.82E-07
SMC4	-1.527574009	2.634427855	-14.92757217	1.06E-39
CENPU	-1.526720862	3.126037934	-16.7384124	4.04E-47
KPNA2	-1.525102779	5.385844507	-20.34034638	4.18E-62
EXO1	-1.518014068	1.711341459	-22.90770363	1.02E-72
AURKA	-1.51583896	3.833241667	-16.55277781	2.37E-46
OIP5	-1.509398701	1.961219378	-21.60767515	2.31E-67
CCNE1	-1.506322759	2.057007406	-12.32159132	1.99E-29
CKAP2L	-1.503820293	1.497578883	-23.95929981	5.22E-77
HELLS	-1.503205163	1.799692159	-20.3929414	2.53E-62
CLGN	-1.494344669	2.455669265	-9.43382347	4.66E-19
SGO1	-1.491103359	1.549976022	-24.28212499	2.56E-78
DDR1	-1.485095638	3.430078504	-8.918159755	2.28E-17
PYCR1	-1.479458932	3.3227288	-7.101738249	6.44E-12
NCAPD2	-1.471750256	3.339702905	-18.93512293	2.99E-56
ARHGAP11A	-1.471171865	1.761845846	-22.40663714	1.17E-70
MCM3	-1.470919185	5.275544922	-17.69592924	4.32E-51
IGHG1	-1.470773658	5.902326198	-4.125186233	4.59E-05
DNMT1	-1.469583979	3.749950834	-18.18730464	3.90E-53
CLDN4	-1.467314127	2.625798342	-6.37112755	5.63E-10
EZH2	-1.463408332	2.743409149	-20.65037847	2.15E-63
SHCBP1	-1.46148165	1.485091238	-19.38188099	4.10E-58
VIL1	-1.458918695	3.280690424	-7.530759319	3.97E-13
E2F8	-1.453079903	1.373933493	-21.47051142	8.52E-67
FANCD2	-1.450405023	1.805301633	-22.78486658	3.26E-72
CCL20	-1.450192382	4.945224441	-5.922716384	7.29E-09
KNTC1	-1.445748751	2.00610618	-19.77554935	9.38E-60
RFC4	-1.440201741	4.289854009	-19.38692718	3.91E-58
FAM111B	-1.434048298	2.126996498	-16.2263817	5.24E-45
IGF2BP2	-1.429264278	2.551878945	-8.779798398	6.34E-17
DEPDC1	-1.428284659	1.351352728	-20.52920774	6.85E-63
TEDC2	-1.424408392	2.195141293	-17.50155862	2.78E-50
CKS2	-1.42377179	5.551140622	-16.6101132	1.37E-46
FAM83D	-1.422069344	3.16284228	-14.13269369	1.67E-36
ORC1	-1.421832722	1.616172327	-20.72596013	1.04E-63
CCNF	-1.421504491	2.161686587	-22.0037653	5.33E-69
CHAF1B	-1.404555278	1.71440828	-17.33819046	1.32E-49
MCM5	-1.40350153	4.519832098	-18.02907993	1.78E-52
PAFAH1B3	-1.396379203	4.4822117	-10.95560765	2.37E-24
RAD51	-1.393701474	1.713281535	-21.69955468	9.62E-68
LRRC1	-1.385214549	2.499225242	-11.0829681	8.18E-25
MCM10	-1.38510192	1.256764603	-22.12864041	1.63E-69
NRSN2	-1.383815536	3.033021522	-9.013838802	1.12E-17
NRM	-1.37931665	3.705510015	-14.25800347	5.28E-37
BEX2	-1.379167679	2.440118319	-6.50531428	2.55E-10
MMP9	-1.377914058	2.863672792	-7.768558519	8.05E-14
WDR76	-1.377256539	1.897388523	-18.30817189	1.22E-53
SSX1	-1.376631416	1.777105167	-5.654707329	3.15E-08
RECQL4	-1.376355004	3.190672256	-14.57506289	2.81E-38
H2AFX	-1.374711611	4.844368142	-16.06955508	2.31E-44

HMGA1	-1.368772547	5.886239597	-11.78916394	2.04E-27
MAD2L1	-1.367753592	1.835481483	-20.52180548	7.35E-63
MDK	-1.36679499	7.008034598	-7.475242994	5.73E-13
RAD54L	-1.359788543	1.198121961	-19.96403371	1.54E-60
PARPBP	-1.359044037	1.563673021	-23.17238779	8.42E-74
WDR62	-1.350411951	1.567434731	-21.33002099	3.25E-66
FEN1	-1.350071821	4.539838057	-18.03707031	1.65E-52
ESPL1	-1.348288113	1.870737218	-15.79462046	3.11E-43
UBD	-1.346424233	6.308402505	-7.095670147	6.70E-12
SPINT1	-1.344795827	2.55866848	-5.960395548	5.91E-09
PIF1	-1.344699719	1.45559353	-19.86341121	4.04E-60
KIF15	-1.341926525	1.187861259	-21.06024659	4.27E-65
CAPG	-1.338537183	4.797904301	-8.473793189	5.85E-16
CDCA7	-1.338451295	1.230018232	-10.90455401	3.62E-24
IQGAP3	-1.337817937	2.481470243	-13.24284425	5.51E-33
GAS2L3	-1.33733641	1.777932613	-18.55064703	1.20E-54
MCM7	-1.334975974	5.572717908	-16.80321881	2.18E-47
RNASEH2A	-1.33469384	4.584939017	-16.89189181	9.37E-48
CENPH	-1.332964646	2.697262679	-19.97319817	1.41E-60
GINS2	-1.332603291	2.621424396	-16.67902242	7.12E-47
ELOVL7	-1.3311401	1.662400992	-8.921516586	2.23E-17
KIF18A	-1.330622094	1.187884198	-22.22473162	6.54E-70
NT5DC2	-1.330424932	4.062359629	-9.468588746	3.57E-19
TRIM31	-1.313768981	2.547294827	-8.389983825	1.07E-15
EME1	-1.310905821	1.447276047	-21.00327924	7.36E-65
SFN	-1.309615614	4.121098636	-5.501842521	7.07E-08
NDRG1	-1.308310638	6.273590479	-10.061046	3.45E-21
EGLN3	-1.307877048	2.135669901	-8.711892025	1.04E-16
PAQR4	-1.306356284	2.316893444	-13.18059404	9.65E-33
PLEKHB1	-1.304810916	1.347881495	-8.448989605	6.99E-16
TRNP1	-1.303973912	3.305257399	-6.806103306	4.11E-11
SPDL1	-1.302497998	2.46916959	-20.9557487	1.16E-64
CIP2A	-1.300742171	1.270994745	-20.93808982	1.37E-64
SLC1A5	-1.300452061	3.432417186	-8.749114657	7.94E-17
NCAPG2	-1.293020527	2.371747067	-18.56339554	1.06E-54
SGO2	-1.292602834	1.495240815	-21.88209948	1.69E-68
PRIM1	-1.291398832	3.451336633	-15.68175905	9.02E-43
PHF19	-1.291390586	2.568630755	-17.60913341	9.92E-51
IGHV3-23	-1.29035815	2.814491726	-5.047990616	7.05E-07
TRAIP	-1.285248169	2.05452414	-17.53931137	1.94E-50
CKAP2	-1.284196922	2.71760661	-17.42687297	5.67E-50
CDC25A	-1.284105956	1.47023019	-18.73628247	2.01E-55
CHEK1	-1.280413202	2.119682017	-19.61022055	4.58E-59
PITX1	-1.280279168	1.513263954	-7.865276219	4.17E-14
INAVA	-1.276366482	1.432640561	-9.445748398	4.25E-19
MAPK13	-1.265066267	2.012498952	-8.262994169	2.63E-15
SOX4	-1.261357855	3.086865266	-8.334053384	1.59E-15
IGKV3-20	-1.260980378	3.676841695	-4.342501319	1.83E-05
INCENP	-1.260231543	2.358611545	-16.87601609	1.09E-47
CTNND2	-1.254280738	1.193261234	-7.798789254	6.56E-14
KCTD17	-1.252715497	3.216294402	-8.557362303	3.21E-16
CDC25B	-1.251641524	4.658126456	-13.83613427	2.53E-35
H2AFZ	-1.250069565	6.5457964	-17.46527239	3.93E-50

DKK1	-1.246562965	1.686229656	-5.484368012	7.75E-08
CENPL	-1.246296834	2.055679221	-18.40289838	4.93E-54
NEURL3	-1.244147437	2.142084128	-8.763771351	7.13E-17
IGSF3	-1.241905345	1.935578067	-10.28980612	5.53E-22
COX7B2	-1.240933327	2.024892743	-4.727408531	3.25E-06
CDKN2A	-1.2401141	3.989274425	-7.682878642	1.44E-13
IGHV1-18	-1.233460228	2.304926779	-5.072892909	6.24E-07
MELTF	-1.233161973	1.951507726	-8.771775256	6.72E-17
NSD2	-1.232436637	3.040722027	-16.96081837	4.86E-48
MTFR2	-1.23107045	1.322356205	-20.17070135	2.12E-61
CDKN2C	-1.229487409	3.414283607	-12.12724999	1.09E-28
MEP1A	-1.229102013	1.167150967	-7.035676622	9.80E-12
OR2I1P	-1.22880182	5.972087994	-5.809316564	1.36E-08
PLP2	-1.227968824	5.552680069	-8.468063102	6.10E-16
IGHV4-39	-1.225511141	2.421166423	-4.976197454	9.99E-07
PIMREG	-1.225221385	1.123926607	-13.20469204	7.77E-33
RASSF3	-1.222313001	3.687080281	-11.79154016	1.99E-27
NREP	-1.220699718	4.090635013	-10.33400408	3.87E-22
IGHG4	-1.220641196	3.246769527	-4.533183641	7.88E-06
LAPTM4B	-1.220039457	5.70720802	-8.506377569	4.63E-16
ARID3A	-1.21911212	2.11472115	-9.261803618	1.73E-18
ATAD2	-1.216149778	3.883921244	-11.99513738	3.43E-28
SPINDOC	-1.21597399	3.044183699	-14.4837621	6.56E-38
RIBC2	-1.209919594	1.16060736	-13.80722781	3.30E-35
HKDC1	-1.209576267	3.758828105	-6.754850662	5.63E-11
FANCG	-1.205599973	2.995803433	-18.11349814	7.91E-53
CTHRC1	-1.205483643	2.644274528	-6.912698794	2.12E-11
CDC7	-1.204274035	1.695116895	-16.96707276	4.58E-48
UBE2S	-1.201300524	3.446349836	-13.75760385	5.19E-35
SLC16A3	-1.20041741	2.688401271	-8.795871282	5.63E-17
POLD1	-1.199740351	3.578223896	-17.98010677	2.84E-52
FADS1	-1.199578285	4.97475737	-7.371215944	1.13E-12
LMNB2	-1.199471329	3.421251088	-14.29281174	3.83E-37
MMD	-1.196685381	3.487628206	-11.76684025	2.47E-27
SGCE	-1.19591028	3.691242717	-6.782865956	4.74E-11
C17orf53	-1.195644136	1.584751919	-19.07262295	7.98E-57
PSRC1	-1.195288415	2.457170685	-13.54051129	3.74E-34
PSPH	-1.188106026	4.193883014	-12.3198186	2.02E-29
SLC38A1	-1.187239958	2.797455076	-7.945579973	2.40E-14
ANXA13	-1.186763681	3.508264044	-5.497114406	7.25E-08
NEIL3	-1.186504907	1.158621695	-16.70982821	5.31E-47
PCNA	-1.184900847	6.466202382	-16.28466196	3.02E-45
CHAF1A	-1.184851227	3.360156546	-17.44518755	4.76E-50
SH3BP1	-1.181220777	2.255873051	-11.77124514	2.37E-27
SLC29A4	-1.181204436	2.131060257	-6.837526925	3.38E-11
ARHGEF2	-1.180419556	3.511280397	-11.94200992	5.44E-28
CDCA2	-1.1802884	1.077043095	-19.47070539	1.75E-58
IGKV3-11	-1.178827744	3.03409214	-4.416406726	1.32E-05
CDCA4	-1.178080626	2.484734997	-17.19543719	5.18E-49
CDK2	-1.177157058	3.812208576	-15.71230174	6.76E-43
GTSF1	-1.174980579	1.265605121	-6.048106528	3.62E-09
STIL	-1.17292681	1.358450013	-19.44584814	2.22E-58
SKP2	-1.172904294	3.320362827	-13.41816356	1.13E-33

CDCA7L	-1.172111729	2.070482127	-11.56930898	1.34E-26
LIG1	-1.170341577	3.84961117	-17.60799333	1.00E-50
SPATS2	-1.169544594	3.374976534	-16.86427671	1.22E-47
SCGN	-1.168393371	2.852939856	-5.640294721	3.40E-08
KIF12	-1.167381958	3.743191069	-7.727563484	1.06E-13
NEMP1	-1.162024855	2.390596139	-16.38505581	1.16E-45
RGS1	-1.161712917	3.107892998	-7.051347026	8.87E-12
CTAG2	-1.160079174	1.275557428	-5.322508295	1.79E-07
IGLV1-44	-1.157062949	2.466584343	-4.778358062	2.56E-06
IGHV3-21	-1.156133465	1.899926931	-5.438900589	9.82E-08
STK26	-1.155167407	2.038910126	-10.80937666	7.96E-24
PSMC3IP	-1.154077815	1.753402942	-17.56530204	1.51E-50
AGR2	-1.153231915	1.377302357	-5.031110775	7.65E-07
CENPO	-1.152487445	1.864899164	-17.77446533	2.04E-51
CBX1	-1.147407021	4.635543785	-16.66966769	7.78E-47
ASRGL1	-1.146509168	2.362155682	-9.829367976	2.16E-20
NUP210	-1.146225465	3.920862923	-14.52416578	4.51E-38
RHNO1	-1.145406819	3.531373473	-17.14955181	8.03E-49
CCDC34	-1.144476549	3.335270581	-12.9972245	5.00E-32
ITPKA	-1.144403491	2.817745165	-8.441285127	7.39E-16
EPS8L3	-1.14400914	2.962484875	-5.71124133	2.32E-08
TIMELESS	-1.141033259	3.792354936	-16.29569127	2.72E-45
TTLL4	-1.14035712	3.005514302	-11.01117613	1.49E-24
MXD3	-1.140260333	3.109559608	-14.72999554	6.68E-39
FBXO5	-1.13811857	1.828061389	-16.36343887	1.43E-45
BMF	-1.137530783	2.536847047	-9.667613918	7.65E-20
CENPI	-1.137248537	1.118176099	-20.24692063	1.02E-61
EPB41L2	-1.136978482	3.540751733	-11.77143954	2.37E-27
MARCKS	-1.136607186	5.052885687	-10.52148674	8.46E-23
CENPE	-1.134975633	1.081646756	-20.21351112	1.41E-61
GINS4	-1.1321969	1.206707755	-17.24209098	3.32E-49
PLCB1	-1.131992957	2.142632535	-11.42328604	4.64E-26
BRCA1	-1.13177831	1.60443813	-19.23491792	1.68E-57
IGKV1-5	-1.131391047	2.921905937	-4.234611027	2.90E-05
AL390728.4	-1.130831617	3.125738716	-9.810828261	2.50E-20
IGKV4-1	-1.129173679	3.184460947	-4.05179388	6.21E-05
ZNF83	-1.128559382	2.302926649	-8.367154197	1.26E-15
TUBA1C	-1.128149483	4.529498187	-12.20816164	5.37E-29
NCK2	-1.127666407	3.565758953	-8.337625409	1.55E-15
MSH2	-1.127583004	3.250316445	-16.14568196	1.13E-44
IGKC	-1.12551522	6.258162851	-3.345607113	0.000905926
LINC01419	-1.124821214	1.762506658	-4.833487543	1.98E-06
LINC00665	-1.123444162	2.182175449	-7.169302574	4.19E-12
PRAME	-1.122325707	1.169906297	-5.732941012	2.06E-08
ZNF468	-1.122021917	1.939915566	-10.49149769	1.08E-22
PLBD1	-1.120435097	2.264321795	-7.959456612	2.18E-14
POLE2	-1.119531035	2.391452722	-13.93500827	1.03E-35
C15orf48	-1.118926326	2.768348827	-5.819281962	1.29E-08
GNAZ	-1.118794698	2.408070771	-7.912691585	3.01E-14
CDK4	-1.117760091	5.210475283	-13.20241739	7.93E-33
PLK4	-1.117399927	1.205057077	-20.0265673	8.45E-61
RFC3	-1.116577365	2.864898514	-14.92334578	1.10E-39
IL4I1	-1.115483766	1.617328951	-9.635518507	9.82E-20

PRKCD	-1.113394281	3.749562642	-13.56663961	2.95E-34
VEGFB	-1.111668669	5.384208437	-7.021599776	1.07E-11
IGF2BP3	-1.109717619	0.97070074	-9.435651736	4.59E-19
MIS18A	-1.109282715	3.274705269	-16.71935404	4.85E-47
ZBTB12	-1.10854036	1.972980743	-13.0436198	3.30E-32
MND1	-1.108330581	2.430529785	-13.12759878	1.55E-32
SRC	-1.107831315	3.452937083	-8.433522605	7.81E-16
PRR15L	-1.10648015	2.205706226	-5.736872472	2.02E-08
KIF14	-1.106043338	1.094334523	-18.55704284	1.12E-54
DSN1	-1.105912238	3.800636974	-16.50873576	3.59E-46
SAPCD2	-1.104687813	1.136078437	-12.79926639	2.93E-31
TREM2	-1.103348616	2.881408087	-7.982119733	1.87E-14
MAGEA6	-1.102811182	1.318406076	-4.981121019	9.76E-07
TOPBP1	-1.102788612	3.100194624	-15.81505284	2.57E-43
TCEAL8	-1.102532586	5.019356927	-7.431135528	7.66E-13
IGLV2-23	-1.099775269	2.601345347	-4.344030076	1.81E-05
MAGEA3	-1.098522856	1.458013978	-4.757439665	2.82E-06
CTSV	-1.098295606	0.946232501	-9.946779507	8.55E-21
AP1M2	-1.097041629	2.566978345	-4.531439057	7.94E-06
TMCO3	-1.096520695	4.301149635	-10.36751808	2.95E-22
PM20D2	-1.09458402	2.369439834	-13.08451921	2.29E-32
MMP7	-1.091679249	2.336620041	-4.493249687	9.42E-06
POSTN	-1.089659861	2.515766431	-5.647865339	3.26E-08
ARHGEF39	-1.087765481	1.609540691	-17.85146281	9.75E-52
SPINK1	-1.087451293	6.214029976	-2.853530378	0.004569113
C21orf58	-1.086262399	1.742949842	-16.00262281	4.36E-44
PLA2G7	-1.085885033	2.507440377	-9.035476064	9.53E-18
CHML	-1.085528706	2.069628446	-11.75978098	2.62E-27
CENPQ	-1.084947849	2.465317936	-15.46402638	7.00E-42
SMC2	-1.083774703	2.313283443	-15.28798024	3.65E-41
MYO19	-1.083624725	3.360707862	-15.92412506	9.16E-44
IGLV3-1	-1.083429838	1.919958264	-4.820360467	2.10E-06
TUBG1	-1.083041355	5.386703385	-16.30143008	2.57E-45
RCC2	-1.082257801	4.420024838	-13.20515602	7.74E-33
SNX7	-1.081468441	3.580459468	-8.560533653	3.13E-16
ASNS	-1.079839714	2.552330163	-7.225695715	2.91E-12
ITPR3	-1.079816517	1.274665304	-8.750953943	7.83E-17
VCAM1	-1.076480556	3.452680618	-6.396317077	4.86E-10
S100A14	-1.074843272	3.827146451	-4.628502454	5.12E-06
MMP14	-1.073957535	5.042326188	-8.420674281	8.57E-16
CIT	-1.072694666	1.370682711	-17.88030492	7.40E-52
DDX11	-1.070356671	2.536543092	-13.09813584	2.02E-32
DONSON	-1.069916703	3.5464411	-15.86616661	1.58E-43
CHTF18	-1.0685272	2.353186631	-15.15075659	1.32E-40
MCM8	-1.06822161	1.60348284	-17.03063235	2.50E-48
PAGE1	-1.067827737	0.75952345	-5.744185162	1.94E-08
ITM2C	-1.067656472	5.295558911	-7.926056854	2.75E-14
CKAP4	-1.066993224	6.066435698	-11.72401216	3.56E-27
MMP12	-1.066707735	0.878343357	-8.102264072	8.12E-15
ZNF367	-1.0662966	2.354092003	-12.20775677	5.39E-29
PLOD2	-1.063451018	5.190445897	-10.03318604	4.31E-21
NUDT1	-1.06269471	4.332545207	-11.81246188	1.67E-27
DNAJC9	-1.060899462	2.793888492	-14.87574803	1.72E-39

RBBP8	-1.060749822	3.099002022	-13.08655268	2.25E-32
DEK	-1.060499288	5.425855944	-12.51758677	3.55E-30
NXPH4	-1.059813437	1.563435755	-6.572387356	1.70E-10
MMP11	-1.05971505	2.631723099	-7.029522855	1.02E-11
AC026401.3	-1.057430126	2.609614747	-13.56082314	3.11E-34
SEL1L3	-1.05615407	3.19086094	-6.226357282	1.31E-09
MISP	-1.055000978	1.167055078	-5.860159423	1.03E-08
COLCA2	-1.051972563	1.864817412	-8.587331614	2.58E-16
TMEM106C	-1.050729278	6.239861141	-12.19145186	6.22E-29
TMPO	-1.048196221	5.120525275	-13.82122882	2.90E-35
IGHG2	-1.048129824	4.226437289	-3.581556151	0.000387598
TFDP1	-1.047920087	4.541804669	-11.21674534	2.66E-25
PKIB	-1.047860467	2.204462174	-6.483625894	2.90E-10
IKBKE	-1.04730397	2.12533559	-10.34506583	3.54E-22
H2AFY2	-1.044269773	3.332261298	-6.228050421	1.30E-09
SPINT2	-1.04407588	2.651822056	-5.081086928	5.99E-07
TMED3	-1.04272752	1.933920715	-8.412230084	9.10E-16
RGS2	-1.042578521	3.91415764	-6.6207666	1.27E-10
LGALS3BP	-1.041657161	7.640598326	-5.654324244	3.15E-08
PFN2	-1.041413476	2.887772524	-6.618267392	1.29E-10
SOX9	-1.041225908	3.435118682	-5.821954439	1.27E-08
ZNF28	-1.040712263	1.578406029	-11.12125661	5.93E-25
RBL1	-1.039169948	1.502590472	-17.7966349	1.65E-51
WDHD1	-1.038996847	1.222505799	-17.97916242	2.87E-52
LINC02506	-1.038942662	1.325505873	-6.005866932	4.59E-09
C6orf223	-1.038845859	0.945972701	-6.800110123	4.26E-11
CSAG1	-1.038754136	1.272106826	-4.917138826	1.33E-06
WNK2	-1.037488072	1.215198485	-6.570129045	1.73E-10
DBN1	-1.036151995	3.602725377	-7.150066054	4.73E-12
ZIC2	-1.035786737	1.778829559	-7.754048029	8.88E-14
RRM1	-1.035518068	4.612312982	-14.680257	1.06E-38
ACTL6A	-1.035447552	4.578045798	-13.95854436	8.26E-36
APOBEC3B	-1.034552811	1.941993189	-8.500948924	4.82E-16
LPCAT1	-1.034293842	3.529989387	-8.939565126	1.95E-17
DBF4B	-1.03423978	1.970462549	-16.84260803	1.50E-47
ABHD3	-1.033343786	5.001018131	-11.4008358	5.62E-26
BLM	-1.032456012	1.091594302	-18.25377462	2.06E-53
IGLV3-21	-1.032315638	2.396810369	-4.150356164	4.13E-05
MAL2	-1.031326187	5.177591488	-6.525614062	2.26E-10
TMEM132A	-1.030530915	2.223451861	-7.171905758	4.12E-12
USP1	-1.029581708	3.476771801	-14.00846845	5.23E-36
MARCKSL1	-1.029207069	5.917716132	-8.34713784	1.45E-15
NASP	-1.027627168	5.018662052	-15.70618759	7.16E-43
FBLN1	-1.027400282	4.020350932	-5.558481211	5.25E-08
LRIG3	-1.02642403	2.759254193	-10.26255136	6.89E-22
BAK1	-1.025946876	3.710123056	-11.68296838	5.07E-27
IGKV3-15	-1.025233804	2.25781751	-4.419048038	1.31E-05
TPGS2	-1.025205043	3.949953737	-13.27292985	4.20E-33
LARGE2	-1.024658743	2.074011052	-6.830808982	3.53E-11
KNSTRN	-1.02413664	3.591945738	-16.02657347	3.48E-44
GMNN	-1.022676892	5.679167178	-9.52741904	2.27E-19
BLMH	-1.022325542	4.025235442	-7.761909291	8.42E-14
SERPINI1	-1.021799539	2.50499869	-9.265622421	1.68E-18

IGLV3-25	-1.019993072	2.294451606	-4.383782898	1.53E-05
ADAM9	-1.019495204	3.822664779	-8.192352402	4.32E-15
JPT1	-1.019458663	5.150757461	-11.75832486	2.65E-27
IGSF1	-1.019377024	1.252666028	-8.126221634	6.87E-15
IGLV1-47	-1.018747407	2.36283364	-4.171548213	3.78E-05
ENO2	-1.018316411	1.676053429	-8.237553496	3.14E-15
XRCC2	-1.017841221	0.97380878	-19.63883517	3.48E-59
IGLV3-19	-1.017280967	2.768608504	-3.719283183	0.00023106
POLA1	-1.016501964	2.036084734	-16.48855361	4.35E-46
LINC01980	-1.016429772	1.449930141	-5.444521665	9.53E-08
PAQR5	-1.015763307	1.793738039	-6.766161889	5.25E-11
CLIC1	-1.015410403	7.621842497	-10.57399053	5.51E-23
PSIP1	-1.014530902	4.15387621	-12.08118483	1.63E-28
IGF2BP1	-1.01442038	1.43873985	-6.852560323	3.08E-11
ESCO2	-1.013609955	1.023044085	-17.08778574	1.45E-48
IGHV4-59	-1.013569907	2.202887324	-4.257945048	2.62E-05
AP1G2	-1.0135274	2.782389401	-9.673138564	7.33E-20
RHEX	-1.012638703	1.535506094	-6.563003935	1.80E-10
COL5A2	-1.012445403	3.467120615	-8.257336413	2.73E-15
GRB7	-1.012276408	3.427809651	-8.494596329	5.04E-16
SALL2	-1.011185441	1.191593847	-9.73713834	4.45E-20
TCEAL9	-1.010509799	4.84878001	-6.687570886	8.49E-11
S100P	-1.009250428	3.31270775	-3.29267044	0.001088928
TMEM51	-1.009037264	2.585073763	-7.734662527	1.01E-13
NFE2L3	-1.008408704	2.066592675	-9.772302705	3.38E-20
MAB21L4	-1.008076156	3.470551606	-4.949977683	1.13E-06
REEP4	-1.007359586	3.983590186	-13.97708118	6.97E-36
LYPD1	-1.006521999	1.960150913	-6.068541346	3.22E-09
PRKDC	-1.005551271	3.831099603	-12.22146788	4.78E-29
IGHV1-46	-1.004841815	1.51883095	-5.397971727	1.21E-07
PLAUR	-1.004628611	2.715068156	-8.363365238	1.29E-15
CEP131	-1.004535119	3.038362644	-14.64425725	1.48E-38
HMGN4	-1.003447715	4.783244976	-10.17594425	1.38E-21
TUSC3	-1.001434772	1.984981571	-6.487054163	2.84E-10
CASC9	-1.000916684	1.606043731	-5.4506889	9.23E-08
PLXNA1	-1.000599013	2.304033237	-11.14832552	4.73E-25
FKBP10	-1.000341753	3.514389505	-6.221460553	1.35E-09
FANCE	-1.000152446	1.862896549	-13.83428569	2.58E-35
TONSL	-1.000149293	2.390903803	-14.0559981	3.38E-36
HAO2	1.005584187	4.069870111	3.891187307	0.000118475
SULT2A1	1.005622324	7.948334583	4.229807672	2.96E-05
SLC28A1	1.006004206	3.765527918	5.0640948	6.51E-07
CYP39A1	1.006761543	2.71391477	5.559103893	5.23E-08
FMO3	1.007508413	7.429406555	4.812670328	2.18E-06
RBP4	1.009636187	12.32332702	6.569354233	1.74E-10
LDHD	1.009751534	5.849588458	8.360355816	1.32E-15
LINC01018	1.010872269	3.310836477	4.189593111	3.51E-05
AZGP1	1.018859565	8.877498371	6.027527267	4.06E-09
CYP2A7	1.019900378	2.793876451	3.608717894	0.000350449
C7	1.021228161	3.446545766	4.250988838	2.70E-05
DCXR	1.021432988	9.1679832	7.000238888	1.22E-11
AGXT	1.028031404	9.003154367	4.954814838	1.11E-06
CDO1	1.031056091	6.876989234	5.732649269	2.07E-08

UGT2B15	1.031530403	6.92848812	4.259114869	2.61E-05
ANG	1.047489845	8.680754183	7.035711716	9.79E-12
MBL2	1.047502892	4.512217835	5.299738247	2.01E-07
CLEC3B	1.053820335	3.677521096	8.955227984	1.73E-17
HAO1	1.05388752	6.978405183	6.058456791	3.41E-09
OGDHL	1.056680219	4.77689891	5.852631245	1.07E-08
IGSF23	1.05753327	4.087372157	5.918036253	7.49E-09
ALDOB	1.078092048	10.3307401	4.383976249	1.52E-05
CFHR5	1.079084954	5.381394872	4.233274522	2.91E-05
SEC14L2	1.081600796	5.288932136	6.642906036	1.11E-10
CES2	1.082206776	7.557210919	7.187417035	3.73E-12
F12	1.084990773	8.230957037	5.978479948	5.35E-09
FTCD	1.086002614	7.506534926	5.509217145	6.80E-08
NAT2	1.103205453	2.426411375	6.368789187	5.71E-10
APCS	1.105089166	9.785361065	5.039098274	7.36E-07
ANXA10	1.114904142	3.485735675	6.274476133	9.90E-10
UGT2B10	1.120065459	6.459234048	4.964269064	1.06E-06
FABP4	1.122593212	3.28654217	6.131517227	2.25E-09
CYP4A22	1.125627064	5.071960921	6.01115749	4.45E-09
AP001783.1	1.130040642	2.573808598	4.808610482	2.22E-06
CPS1	1.13623893	7.449581233	3.84110558	0.000144295
MFSD2A	1.140789332	3.339296827	4.795635251	2.36E-06
FAM83A-AS1	1.143466634	1.657146565	5.436070314	9.96E-08
HSD17B6	1.14474458	8.007567686	5.473056169	8.22E-08
CHI3L1	1.154695729	6.587188581	3.409392904	0.000723496
SPP2	1.160514899	5.838453584	4.443585118	1.17E-05
THRSP	1.164421942	3.886178043	3.77930884	0.000183505
NNMT	1.166046504	7.118897459	4.469506603	1.05E-05
SAA4	1.168012643	6.892673358	5.410736448	1.14E-07
AL354872.2	1.16870436	3.472177798	8.256642232	2.75E-15
ABCB4	1.172353285	5.179179694	6.780264408	4.81E-11
DNASE1L3	1.176485057	3.21294876	8.567549577	2.98E-16
CYP4F2	1.177733428	5.43751915	5.882864464	9.10E-09
RAMP1	1.177940443	7.015124952	6.79946212	4.28E-11
HRG	1.179230799	8.566524733	4.166331987	3.86E-05
RTP3	1.180172119	4.409523849	5.49320535	7.40E-08
LAMA5-AS1	1.18960665	2.90308431	6.841488686	3.30E-11
C3P1	1.195352729	4.187803987	6.038915353	3.81E-09
MOGAT2	1.217523004	2.975653397	6.269864601	1.02E-09
CYP4A11	1.218190147	6.936980057	5.955303305	6.09E-09
ACSM2B	1.22474338	6.756821764	6.870816896	2.75E-11
CCL16	1.23157147	5.736921821	5.594404542	4.34E-08
APOF	1.236417331	4.902273404	5.606398231	4.07E-08
C8A	1.241437242	6.790805011	7.524378788	4.14E-13
UGT1A4	1.24445295	3.945759809	4.806116147	2.25E-06
RDH16	1.251316309	5.387353328	5.641721076	3.37E-08
AFM	1.256391488	6.204682374	6.171980548	1.79E-09
MT1X	1.259784874	5.673133431	5.533713765	5.98E-08
C4BPA	1.262187903	9.097493916	6.424613643	4.11E-10
CYP1A1	1.270054416	2.55821886	4.791508186	2.41E-06
APOC3	1.276339604	12.69567882	5.890263244	8.73E-09
ALDH1L1	1.278300215	6.710658386	5.61426889	3.90E-08
HJV	1.281089507	6.805222228	6.382221267	5.28E-10

LRCOL1	1.288284765	2.34700947	7.157091412	4.53E-12
ACSM5	1.293331185	4.938330005	6.519080016	2.35E-10
AOX1	1.293376028	7.066677795	5.993987722	4.90E-09
HPX	1.309942768	10.63077352	7.077805717	7.50E-12
SDS	1.318553413	6.671188808	3.933679258	0.000100062
GYS2	1.319483215	3.666173729	6.620739814	1.27E-10
SERPINC1	1.389870108	10.86263121	5.971781031	5.55E-09
CYP2C8	1.389965923	6.671250464	5.652307685	3.19E-08
CYP2C9	1.392735077	7.426196983	5.885040317	8.99E-09
ETNPPL	1.393514909	4.837092924	6.879508067	2.61E-11
GLYATL1	1.401400685	5.281783451	7.756934288	8.71E-14
PON1	1.431186778	6.938199826	6.985259387	1.35E-11
SLC13A5	1.443461998	5.598790743	6.857170281	2.99E-11
ACSM2A	1.451308082	6.272984572	7.768244201	8.07E-14
APOA5	1.465496277	6.878836227	7.28755111	1.95E-12
C6	1.466780998	6.169033909	7.919857131	2.87E-14
PCK1	1.46693039	6.60163466	5.715109138	2.27E-08
LINC01702	1.506164669	3.512630671	7.578177207	2.89E-13
CYP1A2	1.522638593	2.512232267	5.464879072	8.57E-08
MASP2	1.528427476	6.437433447	7.848579077	4.67E-14
ASPDH	1.532874279	5.341900158	8.06119662	1.08E-14
SAA2-SAA4	1.559907873	3.913000962	5.580305165	4.68E-08
SLC25A47	1.564779029	4.751856241	5.476173902	8.08E-08
HGFAC	1.566607341	4.191359114	5.577701023	4.74E-08
AKR7A3	1.567238113	5.243109328	8.049372796	1.17E-14
CFHR3	1.575600145	5.245843117	6.880439355	2.59E-11
LINC00844	1.581838449	3.328278886	6.084654978	2.94E-09
SLC27A5	1.587413618	6.395787642	8.556091932	3.24E-16
LINC01554	1.609892926	2.989518196	5.886689579	8.91E-09
F9	1.629345801	5.883233753	6.988748025	1.32E-11
HPR	1.749475326	7.883547146	8.428961459	8.07E-16
CYP2E1	1.75949585	6.737611216	4.785592052	2.48E-06
LINC01485	1.797528259	6.163464935	7.422208864	8.12E-13
TTC36	1.797611944	3.042759807	8.357616496	1.34E-15
SAA2	1.814911084	5.888231433	5.50618674	6.91E-08
AQP9	1.833565768	6.749789277	7.379439385	1.07E-12
SLC22A1	1.835740451	5.538121106	6.37480365	5.51E-10
CYP8B1	1.838762794	5.491184602	6.443530753	3.68E-10
CFHR4	1.88496049	4.037315756	9.98922724	6.11E-21
CYP2A6	1.897346898	6.723183818	5.177467675	3.72E-07
ADH1B	1.912976375	8.227040729	8.190873679	4.37E-15
HSD11B1	1.916614333	6.639767935	6.472173076	3.10E-10
GLYAT	1.926418567	4.696325208	7.731923713	1.03E-13
ADH1C	1.934377363	7.398271162	6.950267379	1.68E-11
SLC10A1	1.948970912	5.470165633	7.495104027	5.02E-13
HP	1.970787623	11.07168942	8.864497442	3.40E-17
TAT	2.066786268	6.879457705	7.195661502	3.54E-12
SAA1	2.127635445	8.213063121	6.211440958	1.43E-09
ADH4	2.269812381	7.207704692	7.591712449	2.64E-13
CYP3A4	2.288936848	6.01694541	5.95276235	6.17E-09
HPD	2.471213308	8.656599238	8.468622447	6.08E-16

adj.P.Val	B
4.85E-68	151.3488054
2.39E-12	19.19885058
3.44E-80	182.3959842
2.11E-63	140.3181329
1.97E-56	123.7882893
5.56E-62	136.874412
4.60E-74	166.1837464
4.19E-75	169.2087672
3.71E-74	166.5814019
4.63E-78	176.4074651
2.70E-71	159.359696
7.34E-77	173.3622527
1.19E-70	157.7724207
6.87E-50	108.5312004
3.00E-72	161.6695848
8.64E-11	15.46617021
2.17E-73	164.4740551
7.74E-70	155.7798635
4.50E-59	130.0107732
1.81E-54	119.1866357
8.37E-64	141.2690178
7.02E-73	163.2519989
1.67E-68	152.4332989
8.95E-56	122.2525191
2.71E-72	161.8121906
3.71E-74	166.5212595
4.12E-62	137.1985342
1.25E-79	180.1966984
3.23E-74	166.8841276
4.52E-69	153.8089922
8.63E-71	158.1633627
1.64E-65	145.325193
1.75E-81	186.0577795
1.03E-62	138.6385675
5.03E-72	161.1135052
3.23E-74	166.9006714
6.22E-72	160.8624198
2.96E-45	97.66613893
4.34E-74	166.300529
7.67E-66	146.1370616
1.19E-79	180.462796
5.53E-70	156.1462084
1.73E-64	142.9230844
1.66E-62	138.1335615
4.31E-69	153.9047958
4.74E-80	181.6716942
2.86E-69	154.3399004
1.64E-66	147.7336239
2.85E-57	125.7637925
8.37E-51	110.6590764
4.74E-77	173.9313303

1.14E-62	138.5169785
2.29E-70	157.0562359
7.38E-67	148.5822531
3.71E-74	166.6453326
2.07E-69	154.6892251
3.54E-63	139.7560998
4.16E-64	141.9976408
1.37E-59	131.2263328
5.96E-69	153.50954
2.81E-65	144.7679145
5.54E-63	139.2804554
1.15E-57	126.6980854
1.86E-64	142.8328566
2.28E-13	21.65411324
4.36E-10	13.77760771
5.11E-61	134.5897612
1.34E-54	119.4962411
1.55E-65	145.4179405
1.31E-55	121.8463261
1.02E-62	138.6557365
9.64E-06	3.390762285
1.33E-73	165.0684597
2.89E-18	33.52423815
1.88E-09	12.2551778
2.15E-23	45.93328106
4.87E-14	23.26854222
3.35E-60	132.6793219
1.51E-61	135.8265075
7.83E-62	136.5083889
2.40E-63	140.1715955
4.57E-42	90.17619251
1.60E-69	154.9699611
1.23E-59	131.3465584
2.38E-11	16.80096393
1.80E-47	102.8513915
4.00E-07	6.693426406
3.92E-63	139.6401333
7.31E-59	129.505901
2.08E-49	107.3999038
9.75E-71	158.0069633
1.11E-53	117.3559958
1.08E-24	49.07051992
8.26E-67	148.4374866
6.66E-15	25.35802485
4.19E-06	4.255794915
3.07E-53	116.3292398
5.03E-14	23.23545697
8.97E-69	153.0782403
2.29E-72	162.0275907
8.71E-57	124.6216224
1.17E-36	77.531886
1.26E-55	121.8928023
3.21E-61	135.0639133

9.78E-70	155.5182217
8.25E-22	42.1088916
1.25E-40	86.80879031
3.63E-06	4.406064203
2.30E-37	79.18678613
1.23E-44	96.2150587
2.43E-59	130.6355753
1.57E-69	155.0191293
6.77E-44	94.45418966
2.06E-64	142.7165964
2.05E-27	55.63574079
1.52E-73	164.8804862
1.48E-59	131.1381819
1.31E-17	31.93080941
1.41E-74	167.8890323
5.04E-16	28.07854831
6.90E-11	15.69906687
1.31E-53	117.1872101
1.37E-67	150.2905276
1.58E-48	105.3335654
0.00017825	0.367426006
1.56E-50	110.0295695
4.65E-09	11.31469607
1.35E-60	133.5970052
1.89E-55	121.4652341
4.92E-12	18.44140397
7.36E-64	141.4126012
4.39E-69	153.8615673
5.18E-08	8.812674411
4.80E-57	125.2337677
1.81E-55	121.513551
1.42E-42	91.365084
1.31E-15	27.06842639
4.21E-60	132.439961
9.84E-48	103.478535
4.01E-44	94.99776521
3.03E-34	71.85306762
6.62E-61	134.3184582
5.56E-66	146.4762785
4.49E-47	101.9207697
6.92E-50	108.5165811
1.35E-22	44.02169572
9.01E-65	143.589513
4.91E-23	45.07708873
1.73E-66	147.659658
2.58E-16	28.78299769
9.82E-35	73.00187913
2.20E-09	12.09192756
1.10E-12	20.01212316
5.01E-51	111.185778
2.04E-07	7.389227845
5.64E-36	75.92125795
6.09E-42	89.8843276

1.66E-25	51.03502291
4.47E-60	132.3692614
6.96E-12	18.07983379
8.26E-58	127.0372668
1.50E-70	157.5095481
2.61E-63	140.0759682
6.50E-50	108.5929699
7.74E-41	87.29447505
7.16E-11	15.66114551
4.25E-08	9.017169872
2.11E-57	126.0745519
3.07E-62	137.5066348
1.06E-14	24.87136254
2.02E-22	43.60036111
7.81E-31	63.78702353
5.00E-52	113.5062058
6.66E-45	96.83040967
2.96E-45	97.67286909
7.64E-58	127.1249382
2.13E-44	95.65141322
4.93E-16	28.10318183
7.38E-67	148.5696006
1.02E-17	32.19532924
1.86E-14	24.27872394
5.15E-62	136.9636524
4.37E-07	6.602115418
1.29E-19	36.79127325
2.08E-15	26.57643375
1.33E-30	63.22935927
1.25E-14	24.69555602
3.95E-10	13.88043211
7.83E-62	136.5105092
9.14E-62	136.3421298
1.61E-15	26.84580829
4.50E-52	113.6282341
1.64E-65	145.3223978
2.19E-40	86.23373956
3.61E-48	104.5049979
3.74E-06	4.374045988
6.90E-48	103.8387054
1.95E-47	102.7662176
8.62E-53	115.2833149
2.24E-56	123.6513318
5.91E-13	20.66099466
1.20E-17	32.02147642
4.35E-14	23.38837579
2.69E-14	23.88544535
7.65E-05	1.245876458
3.41E-45	97.52199726
9.06E-13	20.21432264
6.00E-15	25.46623693
4.17E-33	69.14634963
1.37E-47	103.1324195

4.76E-07	6.513299613
2.03E-51	112.0921551
1.46E-15	26.95208273
2.30E-20	38.60844232
1.55E-05	2.900957828
1.89E-12	19.44211742
3.34E-06	4.492004459
1.38E-15	27.0101674
1.54E-45	98.32809895
1.17E-58	129.0138766
1.02E-26	53.94632628
1.03E-10	15.28755367
9.31E-08	8.203696686
1.10E-14	24.8307172
5.18E-06	4.036803071
1.09E-30	63.44512928
1.63E-25	51.05535766
1.65E-20	38.96213998
3.52E-05	2.050109066
8.51E-15	25.10284477
4.44E-17	30.63073782
3.06E-26	52.80440828
1.29E-35	75.07879182
5.38E-33	68.8834631
5.31E-10	13.57121799
3.15E-50	109.3237235
2.12E-10	14.52932419
1.46E-45	98.38757028
8.38E-33	68.4325677
1.17E-15	27.18524194
1.10E-49	108.0484363
1.33E-11	17.40763031
7.19E-35	73.32150938
1.98E-25	50.84407534
4.52E-10	13.74001308
3.55E-54	118.5038863
5.72E-32	66.46617029
2.08E-27	55.62027992
3.51E-13	21.20407761
4.47E-07	6.578060706
1.59E-44	95.94374474
8.21E-43	91.91596645
1.66E-47	102.9408682
1.92E-25	50.88174001
3.30E-10	14.07091103
4.73E-26	52.34673159
8.47E-56	122.3156906
5.68E-05	1.553899816
1.73E-46	100.5607323
1.66E-40	86.52065254
2.68E-08	9.497327475
1.06E-55	122.0776995
1.66E-31	65.36252325

9.92E-25	49.16166908
3.62E-48	104.4941167
3.77E-45	97.41044571
2.19E-07	7.31424103
1.43E-12	19.73882647
3.23E-43	92.86564867
9.33E-11	15.38490373
1.04E-06	5.702072959
1.24E-05	3.129390331
5.94E-07	6.283331332
4.26E-22	42.81756944
5.42E-48	104.0867014
4.04E-06	4.294375837
7.51E-49	106.0835527
2.31E-44	95.56265593
7.29E-19	34.97463732
8.94E-36	75.4514366
2.67E-46	100.1238206
6.44E-30	61.59214837
1.32E-14	24.64101771
1.54E-07	7.68491488
7.43E-43	92.02025422
8.68E-23	44.48142027
1.39E-36	77.35405964
3.95E-43	92.66108541
2.40E-18	33.72091408
5.83E-59	129.7425834
1.92E-25	50.8834024
3.94E-21	40.47164004
7.86E-59	129.4231855
1.12E-46	101.0050862
3.20E-24	47.92647756
7.67E-55	120.058007
0.0001169	0.804643006
8.37E-19	34.83032149
0.000235574	0.080025371
2.16E-14	24.11798017
5.25E-27	54.64832195
2.63E-14	23.91050882
2.99E-42	90.60281734
0.002713457	-2.440934722
9.75E-06	3.378996301
4.59E-11	16.12295162
1.38E-07	7.799063194
4.95E-21	40.22920161
3.20E-13	21.29831839
1.71E-33	70.04683869
8.85E-08	8.256821387
4.34E-13	20.9811858
1.11E-30	63.42475642
4.62E-58	127.6354555
2.38E-37	79.14751911
3.01E-18	33.47361096

4.54E-32	66.70229584
1.12E-10	15.20024403
1.29E-17	31.94470546
1.46E-44	96.03415471
4.31E-30	62.00560068
2.11E-30	62.75534207
1.39E-14	24.58610244
1.35E-07	7.819782817
4.74E-52	113.567426
1.02E-43	94.03682537
3.55E-29	59.83421084
2.77E-13	21.45248011
5.06E-06	4.059796562
6.44E-41	87.48666472
9.17E-12	17.79397057
7.60E-05	1.252200576
1.36E-05	3.035340761
3.02E-19	35.89224815
3.54E-05	2.042610686
1.28E-20	39.23089218
3.04E-30	62.37052182
4.15E-05	1.879116172
2.11E-07	7.35360988
3.67E-49	106.8190789
0.011681053	-3.929207734
1.13E-41	89.25309905
2.21E-16	28.94296939
2.11E-25	50.78372723
1.67E-39	84.19162986
8.44E-39	82.54485986
2.33E-41	88.51338995
1.03E-05	3.319332935
7.07E-43	92.07452204
1.09E-30	63.44928509
5.87E-15	25.4888885
3.26E-11	16.47906515
1.59E-15	26.85913841
4.05E-09	11.45961276
2.36E-05	2.463709534
1.51E-14	24.49528303
2.80E-49	107.0946524
2.71E-30	62.49210681
4.01E-41	87.96765037
3.00E-38	81.26411724
8.07E-46	98.99208715
1.30E-07	7.858353777
3.98E-13	21.07168634
2.82E-25	50.47819925
1.26E-13	22.27486631
5.26E-27	54.6448044
1.58E-19	36.57153773
1.38E-25	51.23447703
3.67E-37	78.70547934

2.99E-30	62.38867607
3.93E-28	57.35072329
1.51E-09	12.48524031
1.06E-10	15.2493694
4.77E-32	66.64971845
1.03E-08	10.49073797
7.15E-08	8.475526235
4.88E-15	25.68051768
5.99E-27	54.50318486
4.74E-33	69.01077214
0.001254054	-1.648449091
1.67E-23	46.19216541
2.48E-09	11.96543201
1.52E-20	39.05079264
1.02E-08	10.50028619
3.21E-06	4.530928573
1.61E-14	24.43564547
1.14E-09	12.77090837
2.05E-07	7.387232632
1.16E-09	12.75611076
8.73E-08	8.271080999
3.60E-23	45.39556146
6.11E-49	106.2953068
1.10E-49	108.0394098
3.35E-08	9.265376053
4.09E-10	13.84418075
6.74E-06	3.762543934
1.53E-09	12.47194586
5.16E-11	16.00195694
1.21E-12	19.91526911
2.18E-36	76.89365466
1.39E-33	70.26148272
8.82E-15	25.06423786
4.34E-16	28.23572999
4.60E-45	97.2045679
3.84E-24	47.73723021
8.37E-51	110.6653738
0.000161866	0.467072959
1.96E-09	12.21062751
4.52E-11	16.13934407
8.95E-34	70.71714363
2.47E-14	23.97728938
1.75E-40	86.46320855
3.30E-07	6.891629813
2.82E-20	38.39075032
3.95E-25	50.12813479
5.62E-05	1.564994834
6.03E-31	64.05687485
3.43E-10	14.03013214
9.06E-42	89.47892162
6.60E-18	32.64429481
1.15E-12	19.96772504
4.33E-17	30.65943892

6.48E-05	1.417353078
6.96E-14	22.89712232
2.13E-25	50.77128116
1.08E-13	22.43987965
0.000149061	0.551399305
5.15E-14	23.21112343
1.73E-56	123.9252435
0.000781962	-1.162606334
1.23E-43	93.84562318
5.78E-07	6.311673877
4.98E-10	13.63930393
2.62E-21	40.89698312
1.49E-26	53.54755008
3.02E-10	14.16227822
4.71E-46	99.53589551
0.000106718	0.899219611
2.30E-18	33.76353209
1.59E-09	12.43002467
4.51E-14	23.34892418
9.21E-15	25.01908106
1.44E-18	34.25828515
7.83E-10	13.16807529
0.003210781	-2.611767503
1.36E-12	19.78607843
1.12E-18	34.53094205
5.82E-06	3.914689028
1.18E-33	70.4306121
2.40E-08	9.610019198
4.71E-27	54.7639566
7.21E-07	6.077711668
2.21E-14	24.09133081
3.01E-36	76.56067363
5.41E-20	37.70112047
2.44E-09	11.98540477
5.61E-07	6.34279889
2.90E-23	45.62104048
1.06E-08	10.46313533
4.22E-33	69.12953281
5.91E-34	71.15140532
0.000425547	-0.532404784
0.000119079	0.785232693
3.47E-06	4.450271484
3.29E-07	6.894826644
1.07E-05	3.284447153
1.53E-09	12.46738545
2.26E-14	24.0701701
0.000139132	0.623510432
2.98E-08	9.384153631
0.001145394	-1.553984172
0.0001097	0.870975688
1.27E-10	15.06801099
5.70E-06	3.937174818
1.38E-07	7.797526122

0.00010625	0.903973478
1.03E-10	15.28777149
1.16E-06	5.58961811
3.89E-16	28.35089698
2.53E-08	9.554367136
7.44E-08	8.435153195
5.30E-08	8.787348689
6.48E-05	1.41815966
0.000117504	0.799240197
1.01E-09	12.9021859
4.12E-11	16.23711267
3.87E-08	9.115698125
4.22E-07	6.639669456
4.70E-09	11.30126655
3.89E-06	4.332046608
7.94E-09	10.76291418
5.46E-06	3.981178764
1.72E-08	9.959262121
3.25E-08	9.294355224
1.09E-05	3.266049739
0.000509577	-0.718717991
1.15E-05	3.207343516
6.02E-07	6.269069965
5.03E-07	6.455933902
0.002214649	-2.231688206
5.09E-05	1.668349375
0.000633279	-0.945542293
4.58E-05	1.77809311
6.79E-07	6.14169752
4.53E-14	23.34408492
4.59E-10	13.72431561
5.60E-15	25.53902005
6.35E-08	8.597551852
4.10E-10	13.84026261
0.000152049	0.530606912
4.56E-07	6.558186265
3.22E-10	14.09497403
2.81E-08	9.446742378
8.14E-09	10.7367566
4.36E-08	8.989470403
2.72E-10	14.27344299
2.76E-07	7.076552821
2.60E-07	7.138516415
5.11E-12	18.39974874
1.10E-05	3.254753306
2.18E-07	7.321654994
1.38E-08	10.18520579
3.73E-07	6.764719989
3.45E-09	11.62294897
1.17E-05	3.188699996
6.12E-08	8.637400079
2.50E-07	7.179239696
4.37E-09	11.37846247

4.95E-11	16.04611691
2.03E-09	12.17238868
3.56E-08	9.200383676
7.97E-11	15.54964817
0.000364791	-0.372585322
1.14E-09	12.77074975
4.00E-08	9.079172153
2.07E-07	7.37673168
6.29E-08	8.609266225
2.58E-10	14.32644394
1.18E-12	19.93452385
1.39E-10	14.97546505
2.94E-10	14.19032682
1.10E-12	20.01002379
2.22E-11	16.87207654
4.14E-13	21.02969259
1.51E-07	7.70523447
3.66E-12	18.75177904
5.24E-07	6.414528021
6.57E-13	20.54856609
1.65E-13	21.99279412
2.96E-07	7.003853507
4.96E-07	6.471734817
3.00E-07	6.990442927
1.78E-13	21.91176759
2.57E-10	14.33212623
2.21E-08	9.699099909
6.05E-15	25.45716479
6.24E-08	8.618147925
1.36E-10	14.99700492
1.43E-14	24.55385092
1.20E-05	3.161999265
9.68E-12	17.73626807
2.30E-14	24.05091311
4.28E-07	6.624232474
1.26E-11	17.46051508
4.55E-09	11.33581637
3.10E-09	11.73246474
2.20E-19	36.22553379
2.06E-06	4.992850471
7.03E-14	22.88687011
2.64E-09	11.8987698
1.39E-12	19.7678449
1.71E-10	14.75986972
6.15E-12	18.20896049
7.28E-16	27.68556781
3.91E-11	16.2891428
1.12E-08	10.4067101
3.36E-12	18.84063492
4.42E-08	8.975656061
1.09E-14	24.834684
