# Supplementary material for: A Cell Cycle Progression-Derived Gene Signature to Predict Prognosis and Therapeutic Response in Hepatocellular Carcinoma
Source: Dis Markers. 2021 Oct 21;2021:1986159. doi: 10.1155/2021/1986159 (PMC8553501; doi:10.1155/2021/1986159)
Supplement: Supplementary Materials — Supplementary table 1. Clinical information of HCC patients in TCGA-LIHC cohort. Supplementary table 2. The gene sets of hallmarks of cancer. Supplementary table 3. 549 CCP-relevant genes in TCGA-LIHC cohort. Supplementary table 4. Prognostic CCP-relevant genes in TCGA-LIHC cohort. [file 1986159.f1.zip › 1986159.f1/Supplementary table 4.pdf]

Supplementary table 4. Prognostic CCP-relevant genes in TCGA-LIHC cohort.

ID	HR	HR.95L	HR.95H	pvalue
GINS1	1.515083074	1.314644918	1.746081158	9.55E-09
TOP2A	1.350406392	1.176698194	1.549757987	1.90E-05
NCAPG	1.483639297	1.307758122	1.683174837	8.92E-10
BUB1	1.426419304	1.245425514	1.633716355	2.89E-07
KIF4A	1.320255312	1.169270357	1.490736577	7.34E-06
TPX2	1.531059751	1.337779759	1.752264486	6.15E-10
BUB1B	1.359272813	1.20147064	1.537800857	1.09E-06
NEK2	1.322676151	1.154910399	1.51481206	5.32E-05
KIFC1	1.397747517	1.221186167	1.599836433	1.17E-06
MELK	1.437889648	1.24593282	1.659420641	6.78E-07
PRC1	1.304696185	1.12390321	1.514571825	0.000474433
KIF18B	1.3778294	1.214644222	1.562938201	6.25E-07
CDK1	1.325069361	1.175784764	1.493308014	3.93E-06
PLK1	1.456420042	1.285536699	1.650018502	3.54E-09
HJURP	1.547588844	1.346903761	1.778175472	7.16E-10
FOXM1	1.337700185	1.1863141	1.508404717	2.05E-06
TTK	1.545317296	1.347180417	1.772595202	5.08E-10
CKAP2L	1.366440076	1.184779993	1.575953757	1.79E-05
CCNB2	1.363165316	1.177463999	1.578154135	3.38E-05
NUSAP1	1.263236444	1.092428929	1.460750691	0.001617871
SKA3	1.518648525	1.304838301	1.767493596	6.79E-08
KIF20A	1.563151031	1.373516691	1.778967202	1.29E-11
NUF2	1.3487911	1.192473245	1.525600209	1.93E-06
LMNB1	1.422435552	1.230144237	1.644785091	1.98E-06
CDCA8	1.575700321	1.378518545	1.801086761	2.63E-11
CCNB1	1.507471808	1.322052054	1.718896956	8.84E-10
ZWINT	1.432801419	1.243337395	1.651136621	6.71E-07
FANCI	1.315863327	1.138173949	1.521293206	0.00020842
RRM2	1.347776286	1.201246477	1.512180018	3.73E-07
KIF23	1.328036557	1.192891682	1.478492241	2.21E-07
CENPF	1.353013315	1.181060328	1.55000129	1.30E-05
KIF2C	1.512602271	1.33438393	1.71462319	9.80E-11
KIF11	1.276322528	1.129024505	1.442837766	9.64E-05
EXO1	1.413400887	1.233944983	1.618955542	5.90E-07
SPC25	1.379954905	1.229538864	1.548772141	4.52E-08
DLGAP5	1.381829647	1.237565179	1.5429112	8.99E-09
PRR11	1.44890274	1.284609126	1.634208496	1.55E-09
MKI67	1.308440102	1.16809793	1.46564381	3.42E-06
FANCD2	1.356475922	1.166590288	1.577269198	7.41E-05
ANLN	1.447418302	1.277146443	1.640391164	7.00E-09
GTSE1	1.35516169	1.202240893	1.527533473	6.52E-07
CENPK	1.248997563	1.090057454	1.43111256	0.001366376
TROAP	1.393419807	1.202606576	1.614508681	1.01E-05
MYBL2	1.382160562	1.216903747	1.569859427	6.31E-07
ARHGAP11A	1.274266535	1.110759665	1.461842066	0.000541814
NDC80	1.508027818	1.317666865	1.725889875	2.42E-09
KIF18A	1.383335876	1.229515628	1.556400019	6.83E-08
RAD51AP1	1.37342722	1.188741999	1.586805489	1.66E-05
CENPA	1.427007966	1.275857713	1.596064916	4.83E-10
MCM10	1.536020327	1.341390312	1.758890327	5.34E-10
CCNF	1.498375583	1.289557498	1.741007587	1.29E-07

CDCA5	1.351830493	1.182194014	1.545808607	1.05E-05
NCAPH	1.354464644	1.202054921	1.526198545	6.31E-07
ASF1B	1.309069542	1.143294695	1.498881323	9.68E-05
CDC45	1.331828235	1.169968828	1.516080092	1.46E-05
RAD51	1.38911914	1.199134022	1.6092046	1.19E-05
DTL	1.201818773	1.051896584	1.373108711	0.006846742
OIP5	1.269757982	1.102848246	1.461928546	0.000895545
E2F8	1.221401519	1.059684587	1.407797839	0.005780833
MCM2	1.449364885	1.255240978	1.673510192	4.23E-07
CDC20	1.440062268	1.280385253	1.619652625	1.19E-09
ASPM	1.23552799	1.082438244	1.41026929	0.001726337
WDR62	1.204838423	1.059633125	1.369941721	0.004455522
CDC6	1.366818413	1.186032772	1.575161006	1.58E-05
RACGAP1	1.391342808	1.221123437	1.585290028	7.04E-07
TRIP13	1.408159155	1.253111049	1.582391448	8.88E-09
SKA1	1.282413043	1.145133828	1.436149358	1.66E-05
DEPDC1B	1.243600749	1.090987643	1.417562182	0.001100113
ECT2	1.436104975	1.23927166	1.664201292	1.49E-06
CDT1	1.27259115	1.11703863	1.44980504	0.00029022
KIF15	1.360538436	1.181468113	1.566749721	1.90E-05
CCNA2	1.131825529	1.005761459	1.273690711	0.039848475
EME1	1.304083613	1.133375327	1.500503875	0.000208094
BIRC5	1.244069715	1.124947865	1.375805495	2.11E-05
CDC25C	1.27238015	1.112253168	1.455560023	0.000447678
CDCA3	1.493584436	1.298733471	1.71766919	1.86E-08
ORC6	1.353983587	1.193654433	1.535847817	2.44E-06
HMMR	1.433988872	1.259562884	1.632569609	5.11E-08
ORC1	1.461565642	1.274799716	1.675693915	5.31E-08
EZH2	1.538401942	1.340114543	1.766028543	9.46E-10
UHRF1	1.284340497	1.13001471	1.459742513	0.000127431
DEPDC1	1.377041732	1.200915133	1.578999114	4.61E-06
MAD2L1	1.327538099	1.153974913	1.527205993	7.39E-05
PKMYT1	1.328061483	1.145717645	1.539425801	0.000166405
PBK	1.458706124	1.275554248	1.668156067	3.48E-08
HELLS	1.130987682	1.003029773	1.27526936	0.044500311
KPNA2	1.48991009	1.314974898	1.688117453	3.93E-10
CDKN3	1.297959787	1.131008589	1.489555098	0.000205252
CENPI	1.351745333	1.193428235	1.531064367	2.11E-06
CEP55	1.276423008	1.154050302	1.411771821	2.07E-06
CENPE	1.421833226	1.238437774	1.632387	5.88E-07
PLK4	1.239465614	1.080443697	1.421892703	0.002181208
CENPH	1.337747005	1.161863294	1.54025612	5.21E-05
RAD54L	1.368554496	1.192374608	1.570765928	8.11E-06
TACC3	1.363781108	1.207206444	1.540663505	6.15E-07
PIF1	1.311788006	1.127509643	1.526184528	0.000441761
TCF19	1.2118054	1.054814604	1.392161543	0.006651404
KNTC1	1.253668667	1.101301276	1.427116415	0.000627496
MCM6	1.465011948	1.275158124	1.683132443	6.95E-08
XRCC2	1.412369426	1.212630092	1.645008984	9.07E-06
UBE2C	1.350890076	1.205722835	1.513535238	2.16E-07
CHEK1	1.444938674	1.248123106	1.672789937	8.36E-07
CDCA2	1.43463924	1.261733584	1.631239569	3.63E-08
AURKB	1.231178488	1.122935295	1.349855577	9.45E-06

STIL	1.343888417	1.16691078	1.547707082	4.09E-05
HMGB2	1.286403932	1.116002705	1.482823535	0.000513139
STMN1	1.344036313	1.185948048	1.523197929	3.64E-06
RFC4	1.455560519	1.248100046	1.697505285	1.71E-06
SHCBP1	1.344802085	1.204493132	1.501455341	1.37E-07
BRCA1	1.329950815	1.148378209	1.540232265	0.000140533
UBE2T	1.431795246	1.234090562	1.661172761	2.20E-06
CENPM	1.269547166	1.106103232	1.45714248	0.000688532
C17orf53	1.280380221	1.10499474	1.483602998	0.00100799
MCM4	1.498786444	1.282840333	1.751083706	3.44E-07
NCAPD2	1.514272619	1.322517485	1.733830812	1.90E-09
SPAG5	1.183557023	1.035217887	1.353152071	0.013642177
CDC25A	1.370487825	1.19273839	1.574726607	8.72E-06
NCAPG2	1.404216383	1.215000049	1.622900057	4.28E-06
KIF14	1.303695217	1.131524754	1.50206278	0.000242676
GAS2L3	1.377561618	1.215277428	1.561516711	5.48E-07
CENPL	1.39328281	1.220379414	1.590683166	9.30E-07
WDR76	1.263683369	1.092121538	1.46219592	0.001668302
BLM	1.274014559	1.099395453	1.476368754	0.001282454
TYMS	1.304604848	1.125851026	1.511739804	0.000405334
DNMT1	1.29055016	1.135941336	1.466202226	8.94E-05
FANCG	1.363295216	1.177598171	1.578275078	3.35E-05
FEN1	1.301911867	1.127032292	1.503927192	0.00033723
PTTG1	1.490747951	1.291441471	1.720813141	4.96E-08
MCM5	1.364562263	1.173966093	1.586102172	5.13E-05
POLD1	1.417296493	1.222133789	1.643624754	3.95E-06
WDHD1	1.407618036	1.236374835	1.602579153	2.39E-07
RMI2	1.184506528	1.041481969	1.347172354	0.009907887
CIT	1.315794613	1.149217423	1.506516895	7.07E-05
RBL1	1.424954065	1.232739477	1.647139665	1.67E-06
CENPO	1.467528162	1.286354518	1.674218792	1.16E-08
MCM3	1.381994251	1.183147355	1.614260558	4.47E-05
PHF19	1.335027797	1.165815916	1.528799867	2.93E-05
LIG1	1.233908296	1.053492181	1.445221626	0.009158628
PSMC3IP	1.324708896	1.185944259	1.479710067	6.34E-07
TRAIP	1.409700282	1.220426709	1.62832792	3.04E-06
H2AFZ	1.370771431	1.18627662	1.583959662	1.90E-05
CHAF1A	1.277893327	1.102799021	1.480787816	0.001108528
CENPW	1.314025914	1.158731839	1.490132613	2.08E-05
CKAP2	1.295088925	1.124588871	1.491438663	0.000330361
CHAF1B	1.350593222	1.165319385	1.5653237	6.54E-05
GINS4	1.215181266	1.055392181	1.399162828	0.006739015
CDCA4	1.418754342	1.262494412	1.594354686	4.23E-09
ESCO2	1.313079157	1.164009376	1.481239677	9.42E-06
MCM8	1.366319348	1.183822852	1.576949251	1.98E-05
CDC7	1.390631826	1.205949558	1.60359682	5.74E-06
RNASEH2A	1.301210048	1.133985028	1.493095189	0.000175758
E2F1	1.212795736	1.077774119	1.36473262	0.001356791
INCENP	1.419543487	1.223897444	1.646464514	3.65E-06
SPATS2	1.522255615	1.303129327	1.778228846	1.17E-07
DBF4B	1.352598516	1.190830266	1.536342162	3.36E-06
MCM7	1.154839968	1.046763019	1.274075726	0.004084161
MIS18A	1.320983468	1.123825316	1.552730036	0.000736846

NEIL3	1.457934485	1.287197458	1.65131849	2.98E-09
GINS2	1.242547526	1.072517282	1.439533311	0.003823004
CBX1	1.374142571	1.190788802	1.585728553	1.36E-05
CKS2	1.449705952	1.266680052	1.659177742	6.93E-08
AURKA	1.156679338	1.032930014	1.295254348	0.01169679
DSN1	1.293997959	1.115325715	1.501293026	0.000674721
POLA1	1.460548433	1.266981411	1.683688259	1.77E-07
FBXO5	1.406505772	1.242497134	1.59216342	6.96E-08
TUBG1	1.353628632	1.220761084	1.500957474	9.24E-09
TIMELESS	1.21394763	1.051469836	1.401532214	0.008179704
PCNA	1.441906247	1.238770456	1.678352607	2.32E-06
FAM111B	1.256366982	1.09526205	1.441169257	0.001115852
MSH2	1.538736965	1.32582382	1.785841687	1.41E-08
TK1	1.2454024	1.078149862	1.438600693	0.002857867
H2AFX	1.328159658	1.143804599	1.542228523	0.000197481
MYO19	1.29293325	1.114814432	1.499510898	0.000680969
DONSON	1.256509807	1.069741843	1.475885893	0.005417719
TOPBP1	1.399489159	1.200640209	1.631271293	1.72E-05
ESPL1	1.367161136	1.190807944	1.569631426	9.07E-06
CDK2	1.387864036	1.197584628	1.608376173	1.32E-05
NASP	1.419384619	1.226628699	1.642430752	2.56E-06
PRIM1	1.405973275	1.211704783	1.631388171	7.09E-06
CENPQ	1.349863722	1.165542332	1.563334095	6.20E-05
SMC2	1.286123806	1.125144855	1.470134655	0.000225838
CHTF18	1.174212266	1.024908736	1.345265581	0.020637345
SMC4	1.304620702	1.143979224	1.487820006	7.30E-05
DNAJC9	1.276983303	1.102052779	1.479680817	0.001142998
TUBA1B	1.357586851	1.194360797	1.543120021	2.90E-06
MXD3	1.340543063	1.144734242	1.56984533	0.000274983
RRM1	1.37915184	1.209939408	1.572028968	1.48E-06
RECQL4	1.138050796	1.020476958	1.269170855	0.020109902
NUP210	1.278074164	1.116886839	1.462523787	0.000360873
LMNB2	1.266898983	1.142393206	1.404974247	7.39E-06
NRM	1.217477529	1.063306999	1.394001482	0.004392094
FAM83D	1.445116692	1.241153272	1.682598194	2.11E-06
TONSL	1.399451082	1.239755676	1.579717172	5.44E-08
USP1	1.409393095	1.224147988	1.622670554	1.82E-06
REEP4	1.166165826	1.055993054	1.28783303	0.002397673
ACTL6A	1.358715635	1.195060748	1.544781869	2.85E-06
POLE2	1.175144869	1.020454142	1.353285175	0.025017615
CDC25B	1.310675356	1.161155355	1.479448792	1.20E-05
FANCE	1.318044408	1.167790491	1.487630766	7.76E-06
RIBC2	1.387348236	1.206591231	1.595184084	4.29E-06
UBE2S	1.329327967	1.178745125	1.49914753	3.47E-06
PRKCD	1.449225941	1.25018246	1.679959442	8.56E-07
PSRC1	1.508909873	1.340953533	1.697902984	8.33E-12
SKP2	1.339932113	1.175764512	1.527021821	1.14E-05
IQGAP3	1.453638863	1.258392358	1.679178939	3.71E-07
RCC2	1.417152308	1.226625294	1.637273155	2.21E-06
CDK4	1.412789142	1.225292119	1.628977392	1.97E-06
PAQR4	1.311682092	1.14619919	1.501056644	8.04E-05
MND1	1.205136843	1.06175438	1.3678821	0.003887582
DDX11	1.116248691	1.000259491	1.245687896	0.049460669

RBBP8	1.298794834	1.118545969	1.508090026	0.000604475
PM20D2	1.328018571	1.147365614	1.537115373	0.000143169
ZBTB12	1.277340512	1.107135508	1.473711909	0.000794021
CCDC34	1.212504301	1.072071969	1.371332078	0.002154568
DEK	1.235391129	1.049678642	1.45396046	0.010981186
PSPH	1.260209054	1.100235529	1.443442625	0.000840463
PRKDC	1.49469413	1.274252247	1.753271809	7.93E-07
TUBA1C	1.309278332	1.183497821	1.448426621	1.70E-07
TMEM106C	1.373267338	1.206563964	1.563003072	1.56E-06
CDKN2C	1.199136791	1.044043307	1.377269537	0.01017287
PSIP1	1.168249416	1.012850969	1.347490145	0.032736526
ARHGEF2	1.325937808	1.157100404	1.519410991	4.91E-05
NUDT1	1.117208404	1.020631118	1.222924322	0.016276417
RASSF3	1.26928746	1.09016487	1.477841287	0.002124837
HMGA1	1.306106676	1.146103954	1.488446702	6.20E-05
EPB41L2	1.239336553	1.077474198	1.425514499	0.002656306
SH3BP1	1.202660011	1.044614073	1.384617669	0.010253371
MMD	1.232435923	1.075591162	1.412152086	0.002619424
CHML	1.308096907	1.142189775	1.498102641	0.000103935
CKAP4	1.186039087	1.03366939	1.360869084	0.015016548
BAK1	1.302051142	1.123122082	1.509486104	0.000466263
CDCA7L	1.316355581	1.189715724	1.456475677	1.00E-07
G6PD	1.361286073	1.237878646	1.496996316	2.00E-10
PLCB1	1.278221157	1.100631405	1.484465479	0.001298558
ABHD3	1.179439693	1.014478785	1.371224328	0.031796067
TFDP1	1.377926811	1.218590972	1.558096474	3.17E-07
PLXNA1	1.363301167	1.19258573	1.558454059	5.62E-06
ZNF28	1.254884245	1.079019803	1.459412017	0.003206784
LRRC1	1.270967121	1.0816731	1.493387811	0.003567003
TTLL4	1.217999722	1.075948565	1.378805058	0.001827331
CDCA7	1.251826896	1.091374848	1.435868327	0.00133026
DSG2	1.116578945	1.004227025	1.241500686	0.041557923
CLIC1	1.286053389	1.114121568	1.484517818	0.000590716
MARCKS	1.379835012	1.218703146	1.562271064	3.74E-07
ZNF468	1.270368319	1.093572494	1.475746395	0.001748842
TMCO3	1.330955246	1.155786483	1.532672249	7.16E-05
IKBKE	1.266757399	1.102394102	1.455626718	0.00085369
IGSF3	1.28754746	1.148614868	1.443284872	1.44E-05
LRIG3	1.223026577	1.073481896	1.393404037	0.002481739
HMGN4	1.207143522	1.037660406	1.404308648	0.014732136
NDRG1	1.432968537	1.273345952	1.612600898	2.37E-09
PLOD2	1.385379147	1.227653105	1.563369469	1.25E-07
CFHR4	0.729702544	0.567405918	0.938421306	0.01408289
ASRGL1	1.161508203	1.037549981	1.300275968	0.00931857
NFE2L3	1.209872737	1.065062961	1.374371368	0.003399663
SALL2	1.224710608	1.043991514	1.436712898	0.012829083
BMF	1.260512669	1.089825001	1.457933327	0.001816814
IL4I1	1.140587964	1.017097548	1.279071911	0.024453459
NT5DC2	1.282845254	1.141810233	1.441300749	2.77E-05
IGF2BP3	1.359806391	1.183952652	1.561779871	1.36E-05
ARID3A	1.31077004	1.128244904	1.522823715	0.00040453
PLA2G7	1.231333973	1.106136412	1.370701964	0.000142478
NRSN2	1.287178827	1.114013444	1.487261524	0.000615708

LPCAT1	1.365315327	1.205787918	1.545948434	9.02E-07
SLC16A3	1.338254553	1.157501174	1.547234068	8.30E-05
IGF2BP2	1.164933549	1.020587062	1.329695645	0.023704398
NEURL3	1.134220489	1.029995023	1.248992557	0.010440295
ITPR3	1.221763679	1.067369641	1.398490673	0.003662827
SLC1A5	1.216920309	1.09970495	1.346629419	0.000145174
EGLN3	1.143942548	1.017270915	1.286387464	0.024707832
DNASE1L3	0.728465268	0.581315813	0.912862913	0.005925699
SNX7	1.395036331	1.217432934	1.598549136	1.65E-06
KCTD17	1.253684756	1.10817649	1.418298874	0.000328423
SLC27A5	0.782188082	0.644237511	0.949678007	0.013082132
LAPTM4B	1.263153549	1.115317293	1.430585628	0.000234625
CAPG	1.161572563	1.008871492	1.33738621	0.037270538
PLP2	1.248905634	1.089268118	1.431938802	0.001445685
SRC	1.349409111	1.149109652	1.584622447	0.000256851
MMP14	1.130405847	1.002971449	1.27403166	0.044581383
ZNF83	1.236373926	1.073811833	1.42354595	0.003176649
PLAUR	1.132467195	1.02196141	1.254922089	0.017565646
LDHD	0.745685078	0.61660523	0.901786439	0.002478548
TTC36	0.805197848	0.657580866	0.985952615	0.036004501
MARCKSL1	1.314735534	1.153430236	1.498599111	4.18E-05
NCK2	1.239878174	1.073347932	1.432245631	0.003479728
SOX4	1.328505369	1.150143996	1.534526565	0.000112583
GAL3ST1	1.161036724	1.011316405	1.332922384	0.034030157
DUSP9	1.257348608	1.106739185	1.428453555	0.000434938
ENO2	1.157467803	1.029359767	1.301519409	0.014546299
ADAM9	1.337763942	1.187909067	1.506523029	1.58E-06
ADH1B	0.801854504	0.643451353	0.999252923	0.049227072
ASPDH	0.790382047	0.634229739	0.984980272	0.036195508
TREM2	1.189543672	1.054668341	1.341667416	0.004701114
PLBD1	1.2299486	1.100585596	1.374516952	0.000261928
SLC38A1	1.286468202	1.11214144	1.488120463	0.0006974
C6	0.783742585	0.649605193	0.945578093	0.010951687
GNAZ	1.263624072	1.096324328	1.456453858	0.001241786
PITX1	1.252048957	1.076902918	1.45568051	0.003460097
MASP2	0.81262335	0.676854452	0.975625864	0.026113331
MMP9	1.122118839	1.00669873	1.250772105	0.037477813
ACSM2A	0.777384285	0.641385897	0.942219543	0.010271196
BLMH	1.251695114	1.095624105	1.429998346	0.000953142
GLYATL1	0.785853295	0.647175768	0.954246793	0.014984975
ZIC2	1.308160191	1.136302927	1.506009572	0.000185373
TMEM51	1.163418039	1.020562543	1.32627005	0.023545093
GLYAT	0.804015839	0.660566293	0.978617098	0.029590067
CDKN2A	1.246920664	1.083748042	1.434661085	0.002043202
AFP	1.182247071	1.043802953	1.339053634	0.008423303
ADH4	0.684979398	0.548853342	0.854867302	0.000816489
VIL1	1.177089176	1.014849221	1.365265795	0.031180179
C8A	0.777088222	0.650723828	0.927991384	0.005347593
MDK	1.204654396	1.034196283	1.403207725	0.016760418
ASNS	1.217308116	1.059489397	1.398635091	0.005509314
TAT	0.784607289	0.627616169	0.98086797	0.033208564
DBN1	1.271518729	1.101499118	1.467781364	0.001038176
PYCR1	1.298641091	1.140180925	1.479123747	8.29E-05

HPX	0.758912172	0.631744342	0.911678421	0.003196858
RGS1	1.194107577	1.035850937	1.376542565	0.01446379
MEP1A	1.137496599	1.030046007	1.25615604	0.010937016
PON1	0.687282922	0.566495553	0.833824401	0.000143035
ADH1C	0.702453495	0.564481738	0.874148585	0.001547952
CTHRC1	1.22460414	1.068786134	1.403138804	0.003522783
CFHR3	0.667986862	0.511827537	0.871790624	0.002979249
ACSM2B	0.807437965	0.667905413	0.976120353	0.027129176
IGF2BP1	1.212693654	1.043194974	1.409732538	0.012056788
TRNP1	1.271625502	1.12632844	1.435665975	0.00010375
CD24	1.288937971	1.128558057	1.47210955	0.000181219
PAQR5	1.155659378	1.023467186	1.304925666	0.019583904
SEC14L2	0.76289032	0.610059532	0.954007944	0.017656203
GYS2	0.762870663	0.625881287	0.929843502	0.007356589
PFN2	1.244927047	1.122174813	1.381106878	3.53E-05
NXPH4	1.166372042	1.013118703	1.342807844	0.032249198
RBP4	0.782164235	0.638733903	0.95780244	0.017449519
MAL2	1.36426683	1.157294131	1.608254924	0.000215454
TUSC3	1.163033224	1.004776435	1.346216166	0.042989122
PKIB	1.267926219	1.106470775	1.45294113	0.000635865
CYP8B1	0.780272441	0.639915152	0.951415324	0.014198357
CLDN4	1.294966945	1.119857988	1.497457184	0.000488235
ANXA10	0.739753302	0.602278892	0.908607217	0.004057752
MOGAT2	0.800912559	0.657534326	0.975555041	0.027392957
H2AFY2	1.180270345	1.003418175	1.388292661	0.0453747
FKBP10	1.224451174	1.066068154	1.406364755	0.004167086
AFM	0.751721437	0.60799599	0.929422445	0.008388934
LYPD1	1.163100392	1.017247748	1.329865339	0.0270975
HAO1	0.755764901	0.621842736	0.918528999	0.004893905
C3P1	0.820844343	0.676260228	0.996340472	0.045819708
SERPINC1	0.81239986	0.66620182	0.990681072	0.040125375
CYP2C9	0.642657647	0.508043954	0.812939212	0.000227002
SOX9	1.193176194	1.039195414	1.369972779	0.012233731
C15orf48	1.187804613	1.048038882	1.346209404	0.00704788
PRAME	1.238679288	1.080485373	1.420034383	0.002137896
CDO1	0.745460965	0.615957775	0.902191793	0.002551696
PCK1	0.787810686	0.640882433	0.968423606	0.02353536
EPS8L3	1.432358143	1.241951265	1.65195681	7.92E-07
LGALS3BP	1.167541197	1.004714938	1.356755429	0.043243926
RDH16	0.76029464	0.602974649	0.958660435	0.020509732
SCGN	1.129949314	1.011668432	1.2620592	0.030341943
HGFAC	0.782343876	0.631247278	0.969607255	0.024970806
FBLN1	1.20269345	1.073663736	1.347229571	0.001435127
FTCD	0.739360859	0.601330415	0.909075054	0.004181536
SFN	1.169075702	1.071005736	1.276125749	0.000474929
HSD17B6	0.819094745	0.673692433	0.995879082	0.045351582
CTAG2	1.256078551	1.073698229	1.469438324	0.004395005
MAGEA6	1.2860037	1.118550104	1.478526095	0.000409394
CSAG1	1.284688646	1.10582178	1.492487258	0.001056867
SPP1	1.162862373	1.056239994	1.280247773	0.00210442
FMO3	0.724273401	0.572148322	0.916846103	0.007325715
MAGEA3	1.330949894	1.140990155	1.552535412	0.000274067
MMP7	1.215915966	1.014446299	1.457397635	0.034417647

ALDOB	0.771617468	0.61953317	0.961035738	0.020623472
UGT2B15	0.819587505	0.673621829	0.997182172	0.046793722
SULT2A1	0.780988138	0.625468918	0.975176309	0.029121946
HRG	0.790932634	0.65114416	0.960731079	0.018093876
CPS1	0.780010171	0.63419452	0.959352136	0.018624209
